# Cross‐Cohort Gut Microbiome Signatures of Irritable Bowel Syndrome Presentation and Treatment

**DOI:** 10.1002/advs.202308313

**Published:** 2024-09-07

**Authors:** Junhui Li, Tarini Shankar Ghosh, Elke Arendt, Fergus Shanahan, Paul W. O'Toole

**Affiliations:** ^1^ APC Microbiome Ireland University College Cork Cork T12 K8AF Ireland; ^2^ School of Microbiology University College Cork Cork T12 K8AF Ireland; ^3^ School of Food and Nutritional Sciences University College Cork Cork T12 K8AF Ireland; ^4^ Department of Medicine University College Cork Cork T12 K8AF Ireland; ^5^ Present address: Indraprastha Institute of Information Technology Delhi New Delhi 110020 India

**Keywords:** diet, gut microbiome, irritable bowel syndrome, oral bacterial translocation, rifaximin

## Abstract

Irritable bowel syndrome (IBS) is a prevalent disorder of gut‐brain interaction without a reliable cure. Evidence suggests that an alteration of the gut microbiome may contribute to IBS pathogenesis, motivating the development of microbiome‐targeted therapies to alleviate IBS symptoms. However, IBS‐specific microbiome signatures are variable across cohorts. A total of 9204 datasets were meta‐analyzed, derived from fourteen IBS microbiome discovery cohorts, three validation cohorts for diet‐microbiome interactions, and five rifaximin therapy cohorts. The consistent bacterial species and functional signatures associated with IBS were identified. Network analysis revealed two distinct IBS‐enriched microbiota clusters; obligate anaerobes that are found commonly in the gut, and facultative anaerobes typically present in the mouth, implying a possible association between oral bacterial translocation to gut and IBS pathogenesis. By analyzing diet‐microbiome interactions, microbiota‐targeted diets that can potentially modulate the altered gut microbiota of IBS subjects toward a healthy status were identified. Furthermore, rifaximin treatment of IBS subjects was linked with a reduction in the abundance of facultatively anaerobic pathobionts. Gut microbiome signatures were identified across IBS cohorts that may inform the development of therapies for microbiome modulation in IBS. The microbiota‐targeted diet patterns described may enable nutritional intervention trials in IBS and for assisting dietary management.

## Graphical abstract

Irritable Bowel Syndrome (IBS) is a prevalent disorder of gut‐brain interaction, affecting ≈11% of the global population. This meta‐analysis identified consistent gut microbiome signatures associated with IBS across studies in distinct geographies and cataloged microbiota‐targeted diet patterns for microbiome modulation in IBS. It also provides insights into the effects of rifaximin on the microbiome in subjects with diarrhea‐predominant IBS.

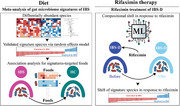

## 1 Introduction

Irritable Bowel Syndrome (IBS) is one of the most common disorders of gut‐brain interaction and one that considerably reduces quality of life,^[^ ^1^ ^]^ affecting roughly 11% of the global population.^[^ ^2^ ^]^ IBS is classified into three major clinical subtypes: IBS with predominant constipation (IBS‐C), IBS predominantly with diarrhea (IBS‐D), and mixed IBS (IBS‐M).^[^ ^3^ ^]^ However, our understanding of its underlying etiology and pathogenesis is poor, and consequently diagnosis and treatment are entirely based on symptoms.

**Figure 1:**
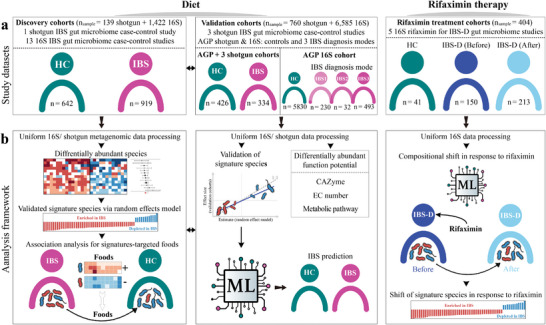
Conceptual framework of study. a) IBS‐related studies examined here, including discovery cohorts (1 shotgun case‐control study and 13 16S case‐control studies) and validation cohorts (3 shotgun case‐control studies and AGP shotgun & 16S study) for diet, as well as rifaximin therapy cohorts (5 16S studies related to rifaximin administration for IBS‐D) in the current analysis. b) Analysis framework of diet and rifaximin therapeutic modulation of the gut microbiome in IBS. AGP indicates American Gut Project; ML indicates machine learning (see the section of 2.6 Random forest classifiers in Method); HC indicates healthy control; IBS‐D indicates irritable bowel syndrome with diarrhea; + indicates positive Spearman association between foods and IBS‐depleted species, – indicates negative Spearman association between foods and IBS‐enriched species. IBS1, self‐diagnosed; IBS2, diagnosed by an alternative medicine practitioner; IBS3, diagnosed by a medical profession.

Recent studies have reported alterations of the gut microbiome^[^ ^4^, ^5^, ^6^, ^7^, ^8^, ^9^, ^10^, ^11^, ^12^, ^13^ ^]^ in subjects with IBS, pointing to a role for the microbiome in the pathogenesis,^[^ ^14^, ^15^, ^16^, ^17^ ^]^ and suggesting that therapeutic modulations of the gut microbiome might alleviate IBS symptoms. Gut microbiome‐targeted regimens/therapies (e.g., diet changes, prebiotics, probiotics, antibiotics, and fecal microbiota transplantation) have been tested for their ability to alleviate IBS symptoms, with varying levels of effectiveness.^[^ ^17^, ^18^ ^]^ Previous meta‐analyses have examined the taxonomic associations of the IBS microbiome at the genus level or a higher taxonomic rank.^[^ ^19^, ^20^ ^]^ However, it is unclear if there are reproducible taxonomic and functional microbiome signatures, particularly at high resolution across cohorts. Uncovering such signatures would be valuable for understanding the pathogenesis of IBS and for developing microbiome‐targeted therapies. Here, we present a meta‐analysis of fourteen discovery cohorts comprising thirteen 16S rRNA gene amplicon sequencing (16S) case‐control cohorts and one shotgun metagenomic cohort to investigate the gut microbiome in IBS subjects (see **Figure** **1** infographic for the study framework). In addition, data from two shotgun metagenomic cohorts and the American Gut Project (AGP) 16S cohort were used as validation cohorts (Figure 1a). We identified consistent microbiome signatures of IBS as well as dietary ingredients that might ameliorate clinical outcomes by modulating the altered microbiome in IBS (Figure 1b).

Rifaximin, a non‐systemic antibiotic, appears to be transiently efficacious in improving symptoms in subjects with IBS‐D, likely due to its influence on certain pathobionts, although no global changes in the overall microbial community composition were previously observed upon rifaximin administration.^[^ ^7^, ^21^, ^22^ ^]^ We also reviewed the effects of rifaximin on the microbiome by examining five 16S cohorts in the current analysis. Collectively, our findings advance understanding of cross‐cohort microbiome alterations in IBS.

## 2 Results

### 2.1 Collating a Large IBS Gut Microbiome Dataset Comprising 2280 Cases and 6836 Controls

To investigate the alteration of the gut microbiome in subjects with IBS, we collected raw sequence data from thirteen 16S and one shotgun metagenomic case‐control studies as discovery cohorts, totaling 1561 gut microbiomes from 642 healthy control (HC) and 919 IBS cases. We also utilized the Mayo Clinic cohort (shotgun metagenome),^[^ ^23^ ^]^ PRJEB19857 (shotgun metagenome),^[^ ^24^ ^]^ PRJNA812699 (shotgun metatranscriptome),^[^ ^25^ ^]^ and the AGP cohort (shotgun metagenome and 16S)^[^ ^26^ ^]^ as validation cohorts, totaling 7345 gut microbiomes from 6256 HC and 1089 IBS cases (Figure 1; Table S1, Supporting Information). In addition, we gathered 16S microbiome data from five cohorts that examined rifaximin therapy for the treatment of IBS‐D, totaling 404 gut microbiomes from 41 HC, 150 IBS‐D patients before rifaximin administration and 213 IBS‐D after rifaximin administration. Among these samples, 104 (36 HC and 68 IBS‐D before rifaximin administration) were also included in the diet discovery cohorts (Figure 1; Table S1, Supporting Information). Altogether, this meta‐analysis thus included a total of 9204 publicly available gut microbiome samples from eleven countries across three continents, encompassing different anthropometrics (e.g., age, sex), and clinical subtypes (IBS‐C, IBS‐D, IBS‐M, IBS‐U without subtype classification, and healthy controls). 16S data were all uniformly reprocessed using SPINGO^[^ ^27^ ^]^ for species‐level taxonomic profiling, while shotgun metagenomic data were annotated with the bioBakery 3 platform,^[^ ^28^ ^]^ employing MetaPhlAn 3 for species‐level taxonomic profiling and HUMAnN 3 for metagenomic functional profiling (see Methods for details).

### 2.2 Reproducible Microbiome Signatures Associated with IBS

**Figure 2:**
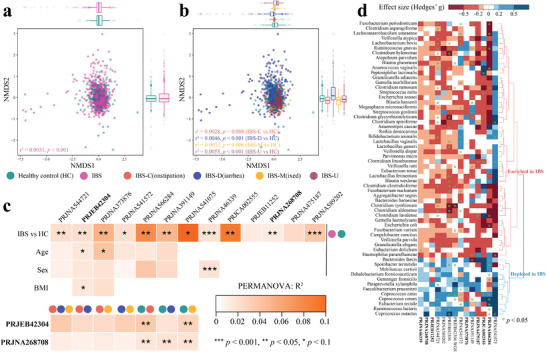
Altered gut microbiome in IBS subjects and microbial species reproducibly associated with IBS across cohorts. a,b) Non‐metric multidimensional scaling (NMDS) of pooled 16S samples (*n* = 1422) based on species‐level Bray‐Curtis dissimilarity. Colored boxplots on the top and the right represent Bray‐Curtis dissimilarity by health status, i.e., (a) healthy control and IBS and (b) healthy control and IBS subtypes, in the first and second ordinations, respectively. IBS‐U indicates IBS without recorded subtype classification except two samples that were already described as unclassified IBS subtype (Table S1, Supporting Information). c) Microbial community compositions of IBS subjects are significantly different from healthy controls. Results of PERMANOVA were based on Bray‐Curtis dissimilarity of relative abundance with covariate adjusted and marginal sums of squares applied: adonis2(relative abundance matrix ∼ Health status + Cohort + Age + Sex + BMI, permutations = 999, method = “bray”, by = “margin”). *** indicates *p* < 0.001; ** indicates *p* < 0.05; * indicates *p* < 0.1. d. Effect size (i.e., Hedges’ G) of 60 retained species in plot e between healthy controls and IBS subjects in each cohort. The color gradient indicates effect size; * indicates *p* < 0.05 (Wilcoxon rank‐sum test).

We first used permutational multivariate analysis of variance (PERMANOVA) with Bray‐Curtis dissimilarity to evaluate gut microbiota differences from controls associated with IBS. After controlling for cohort effects, differences in gut microbiome composition between healthy controls and IBS subjects (across all IBS subtypes) were significant but small based upon the pooled 16S datasets (r^2^ < 0.01, *p* < 0.05, PERMANOVA, **Figure** **2**a,b). Analysis of the combined 16S datasets revealed a significant cohort effect when examining the species level microbiome composition (r^2^ = 0.175, *p* < 0.001, PERMANOVA, Figure S1a, Supporting Information; Figure 2a, Supporting Information). Therefore, we assessed gut microbiota differences between healthy controls and IBS subjects within individual cohorts while controlling for available confounding factors (Figure 2c). Differences in gut microbiota between healthy controls and IBS subjects were significant in 9 out of 13 cohorts (*p* < 0.05, PERMANOVA, Figure 2c), with two additional cohorts marginally significant (*p* < 0.1). Among the investigated cohorts, gut microbiome composition was not significantly associated with age and sex except sex in one dataset (Figure 2c) derived from a study in the United States (PRJNA46339). To test if the gut microbiome composition differs across IBS subtypes, we investigated two representative cohorts (i.e., PRJEB42304 and PRJNA268708) which included at least 20 samples for each IBS subtype (IBS‐C, IBS‐D, and IBS‐M) and HCs (Table S1, Supporting Information). No significant difference in gut microbiota was present across three IBS subtypes, between IBS‐C and IBS‐D, or between IBS‐C and IBS‐M (*p* > 0.05, PERMANOVA, Figure 2c), which is consistent with the previous studies which found that IBS subtypes of IBS did not vary in gut microbial community composition.^[^ ^6^, ^29^ ^]^ Nonetheless, the microbiota of each IBS‐subtype was significantly different from that of healthy controls, except IBS‐D in PRJEB42304 (*p* < 0.05, PERMANOVA, Figure 2c). Consequently, we conducted the downstream analyses with data from combined IBS subtypes. Additionally, to test if the gut microbiome composition differed between subjects diagnosed by the Rome III and IV diagnostic criteria, we investigated 11 16S discovery cohorts with known Rome diagnostic criteria (Table S1, Supporting Information). After controlling for the available confounding factors (i.e., country and sequencing techniques, including 16S rRNA gene region and paired/ single sequencing), differences in gut microbiome composition between Rome III and IV diagnostic criteria were significant but small (r^2^ = 0.011, *p* < 0.001, PERMANOVA, Figure S1b, Supporting Information).

**Figure 3:**
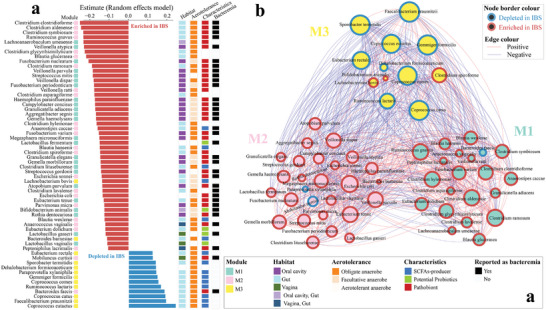
Microbial co‐occurrence network modules and their differential association with IBS. A) The estimated effect size of species retained from random effects meta‐analysis (FDR < 0.1, please see Supplementary Information for random effects models for each species) and their phenotypes, including habitat (oral cavity, gut, vagina, oral cavity & gut, vagina & gut), aerotolerance (obligate anaerobe, facultative anaerobe, aerotolerant anaerobe), characteristics (SCFAs: short‐chain fatty acids producer, potential probiotic, pathobiont), and bacteremia (reported or not reported in cases of bacteremia). B) The co‐occurrence network was constructed using 60 IBS‐related signature species from the microbiomes of the discovery cohorts. Each edge indicates a SparCC correlation with FDR  <  0.01 (Benjamini‐Hochberg correction) with red and blue indicating positive and negative associations, respectively. Color of nodes indicates network module; color of node border indicates the signature species that was depleted (blue) or enriched (red) in IBS; size of nodes is proportional to its number of connections with other nodes (i.e., species). M1, module 1; M2, module 2; M3, module 3.

To identify the species that differentiate IBS from healthy controls, we initially estimated the effect size (Hedges’ G) between healthy control and IBS for each species within individual cohorts. We then selected the putatively informative IBS depleted species level features (i.e., species with Hedges’ G > 0.1 in at least seven studies and < −0.1 in at most three studies; *n* = 25, Figure S2d, Supporting Information), and putatively informative IBS enriched species level features (i.e., species with Hedges’ G < −0.1 in at least seven studies and > 0.1 in at most three studies; *n* = 85, Figure S2d, Supporting Information) across 14 discovery cohorts. We next evaluated the selected features using random‐effects model (REM), identifying 60 statistically significant differentially abundant species (FDR < 0.1, **Figure** **3**a; see Table S2, Supporting Information for species identities), with 54 species (90%) having low heterogeneity (I^2^ value < 25%) and 6 species (10%) having moderate heterogeneity (I^2^ value < 50%, Table S2, Supporting Information).^[^ ^30^ ^]^ Among these 60 signature species identified, 12 (e.g., *Coprococcus* spp., *Eubacterium rectale*, *Faecalibacterium prausnitzii*, *Gemmiger formicilis*, *Paraprevotella xylaniphila*, *Ruminococcus lactaris*, *Sporobacter termitidis*) were depleted in subjects with IBS, while 48 (e.g., *Clostridium* spp., *Fusobacterium* spp., *Gemella* spp., *Streptococcus* spp., *Veillonella* spp., *Aggregatibacter segnis*, *Parvimonas micra*, *Rothia dentocariosa*, and *Ruminococcus gnavus*, which are hallmarks of a disturbed microbiome,^[^ ^31^, ^32^ ^]^ as well as *Blautia* spp., *Escherichia* spp., *Eubacterium* spp., *Granulicatella* spp., *Lactobacillus* spp. including *L. gasseri*, *L. fermentum* that has been reclassified as *Limosilactobacillus fermentum*, and *L. vaginalis* that has been reclassified as *Limosilactobacillus vaginalis*) were enriched in subjects with IBS, respectively (Figure 3a). The taxa depleted in IBS subjects are primarily short‐chain fatty acids (SCFAs) producers (Figure 3a; Table S3, Supporting Information). Although several species enriched in IBS subjects, including *Blautia* sp., *Anaerostipes caccae*, *Bacteroides barnesiae*, and *Lachnobacterium bovis*, are also SCFAs producers, two thirds of the species enriched in IBS subjects are pathobionts (Figure 3a; Table S3, Supporting Information). To validate the identified microbial signatures, we further associated the effect size of the identified microbial signatures between HC and IBS based on centered log‐ratio (CLR)‐transformed read count, considering the compositionality of microbiome data. We found that the effect size of microbial signatures based on relative abundance strongly associates with the effect size based on CLR‐transformed read count across discovery cohorts (on average ρ = 0.792, *p* < 0.0001, Spearman, Figure S3, Supporting Information), suggesting the validity of the employed method in identifying microbial signatures.

Next, we validated the microbiome features by associating their REM estimates with their effect sizes (i.e., Hedges’ G) between healthy controls and IBS in the gut microbiome of two additional cohorts, i.e., Mayo clinic cohort (PRJEB37924; shotgun metagenome: *n* = 22 HC, *n* = 49 IBS baseline samples) and AGP cohort (PRJEB11419; shotgun metagenome: n = 229 healthy controls, n = 21 IBS3 diagnosed by a medical professional; 16S: n = 5830 healthy controls, n = 493 IBS3). We found that the effect sizes in the Mayo clinic cohort subjects were strongly associated with the REM estimates (ρ = 0.65, *p* = 0.0002, Spearman's correlation, Figure S4a, Supporting Information). In contrast, the association in the AGP cohort was not significant, irrespective of the dataset type (shotgun or 16S) (*p* > 0.1, Spearman's correlation, Figure S4b, Supporting Information). Most AGP stool samples were collected on dry swabs and shipped without preservative, leading to the known phenomenon of bacterial bloom in transit,^[^ ^26^ ^]^ which may account for the insignificant associations in the AGP cohort. However, the association in the AGP 16S dataset remained statistically insignificant even after removing the 20 blooming sequences (https://github.com/knightlab‐analyses/bloom‐analyses/blob/master/data/newbloom.all.fna)^[^ ^33^ ^]^ (*p* > 0.1, Spearman's correlation, Figure S4c, Supporting Information). In addition, we validated the microbial signatures in two recently discovered shotgun cohorts used in the functional analysis: PRJEB19857 (shotgun metagenome: n = 56 HC, n = 56 IBS baseline samples) and PRJNA812699 (shotgun metatranscriptome: n = 119 HC, n = 208 IBS). The effect size of microbial signatures in the metagenomic cohort (PRJEB19857) significantly associates with REM estimate (ρ = 0.35, *p* = 0.034, Spearman correlation, Figure S4e, Supporting Information). However, there is no significant association between the effect size of REM species in the metatranscriptomic cohort (PRJNA812699) and REM estimate (*p* > 0.1, Spearman correlation, Figure S4f, Supporting Information). The gut metatranscriptome is temporally more dynamic compared to the gut metagenome.^[^ ^34^ ^]^ This may be related to the lack of significant association.

To test if the gut microbiome data can predict IBS status, we conducted cross‐cohort and leave‐one‐out cross‐validations of 16S microbiome datasets using a RF classifier, and trained the model on species‐level microbiome signatures (Methods). The mean area under the curve (AUC) for the leave‐one‐out cross‐validation was 0.70, representing the optimal predictive accuracy, and for cross‐cohort cross‐validation, 0.68 (Figure S5, Supporting Information). When focusing on within‐cohort predictive accuracy (60% of data was used for training, with the remaining 40% for testing), the average AUC was 0.80 (Figure S5 and Table S5a, Supporting Information).

### 2.3 Co‐Occurrence Network Analysis of Signature Species Reveals Distinct Microbiome Phenotypes

To further characterize these IBS‐related signature species, we generated a co‐occurrence network using SparCC^[^ ^35^ ^]^ to identify microbiome taxa that coalesced into modules (see Methods). This enabled us to cluster the signature species into three modules (Figure 3b). In general, the modules (M) not only represented negative or positive associations between signature species, but the distinct health/disease association across modules also directly translated to distinct microbial phenotypes, despite exceptions. Within‐module species association was predominantly positive, whereas between‐module species association was dependent on the modules themselves, with primarily negative associations between M3 and M1 and between M3 and M2, while primarily positive associations between M1 and M2. Both M1 and M2 were mainly composed of species enriched in IBS, apart from *Bacteroides faecis* (M1) and *Mobiluncus curtisii* and *Paraprevotella xylaniphila* (M2), which had small hub values (i.e., connected nodes within network), whereas M3 was primarily composed of IBS‐depleted species that produce SCFAs (Figure 3; Table S3, Supporting Information). M1 and M2 taxa, however, featured distinct aerotolerance, with M1 primarily composed of obligate anaerobes (e.g., *Clostridium* spp. and *Blautia* spp.), typically inhabiting the human gut and M2 represented by facultative anaerobes (e.g., *Fusobacterium* spp., *Gemella* spp., *Granulicatella* spp.,), aerotolerant anaerobe (e.g., *Lactobacillus* spp., *Streptococcus* spp.), and several obligate anaerobes (e.g., *Veillonella* spp.) found in the human oral cavity (Figure 3a; Table S3, Supporting Information). Oral bacteria can translocate to the gut and disturb its homeostasis, causing microbial dysbiosis.^[^ ^36^ ^]^ Among the M1 and M2 taxa, 72.9% (35/48) were reported in cases of bacteremia, while none of the 12 M3 taxa has been reported in bacteremia to date (Figure 3a; Table S3, Supporting Information). The IBS‐depleted species are primarily commensals in the gut, whereas the IBS‐enriched species predominantly consist of pathobionts inhabiting the oral cavity and/or gut.

### 2.4 Differential Gut Microbiome Functional Potential between Healthy Controls and IBS

**Figure 4:**
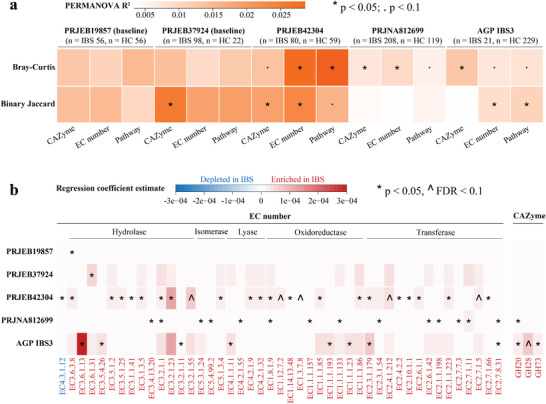
Weak functional signatures in the gut microbiome associated with IBS subjects. a) Microbiome functional compositions were significantly different between IBS and HC, irrespective of weighted (Bray‐Curtis) or unweighted (binary Jaccard) matrix assessed through PERMANOVA with available covariates adjusted and marginal sums of squares. PRJEB19857 (baseline samples): adonis2(relative abundance ∼ Health status + Gender + Age + Smoking + Post infectious, permutations = 999, method = “bray”, by = “margin”); PRJEB37924 (baseline samples): adonis2(relative abundance ∼ Health status + Age + Sex + BMI, permutations = 999, method = “bray”, by = “margin”); PRJEB42304: adonis2(relative abundance ∼ Health status + Age + Sex, permutations = 999, method = “bray”, by = “margin”); PRJNA812699: adonis2(relative abundance ∼ Health status + City + Ethnicity + Gender + Age + BMI + Diet type, permutations = 999, method = “bray”, by = “margin”); and AGP IBS3: adonis2 (relative abundance ∼ Health status + Country + Ethnicity + Gender + Age + BMI + Antibiotic exposure within 6 months, permutations = 999, method = “bray”, by = “margin”). The color gradient of panel a indicates PERMANOVA variation for each cohort; the number on the panel a indicates *p*‐value of PERMANOVA. b) Differential functions between IBS and HC within individual cohorts (linear regression model with available covariates adjusted). The color gradient of panel b indicates the estimate of regression coefficient in each cohort (see Table S4, Supporting Information for details on all functions); *p < 0.05, ^ FDR < 0.1. CAZyme, carbohydrate active enzyme; EC, Enzyme Commission; AGP, American Gut Project; IBS3, IBS diagnosed by a medical professional; PERMANOVA, permutational multivariate analysis of variance.

To assess if the gut microbiome functional potential differed between healthy controls and IBS, we investigated three aspects of function for shotgun metagenome datasets: Pathways, CAZyme, and EC Number assignments. The functional analysis of the AGP cohort was sub‐divided based on diagnosis mode (i.e., IBS1 self‐diagnosed, n = 9; IBS2 diagnosed by an alternative medicine practitioner, n = 0; IBS3 diagnosed by a medical professional, n = 21; **Figure** **4**), and IBS1 and IBS2 were excluded from the subsequent analysis due to small sample size. Significant differences in functional composition between HC and IBS were observed across the PRJEB42304, PRJNA812699, PRJEB37924 (baseline samples), and AGP IBS3 cohorts after adjusting the covariates, albeit the explained variation was small (r^2^ ∼ 0.029, *p* < 0.05, PERMANOVA, Figure 4a). Irrespective of the approach employed (i.e., weighted Bray‐Curtis dissimilarity and unweighted binary Jaccard dissimilarity), these differences were detected in at least one cohort for each functional category, respectively.

We compared the abundance of each functional gene prediction between HC and IBS in the five investigated cohorts to identify IBS‐associated microbiome functions. We thus found many differentially abundant functions between HC and IBS in individual cohorts (linear regression model with covariates adjusted, Table S4, Supporting Information). Differences in a total of 105 EC numbers overlapped in the same direction across five cohorts (Table S4, Supporting Information). Among these 105 EC numbers, 45 were significantly enriched (44) or depleted (1) in at least one cohort (FDR < 0.1 or *p* < 0.05, Figure 4b). The IBS‐enriched EC numbers were mapped to enzymatic pathways related to such microbial functions as hydrolases, isomerases, hydro‐/ carboxy‐lyases, oxidoreductases, and transferases, while the EC 4.3.1.12 (ornithine cyclodeaminase) that was depleted in IBS belongs to the ammonia‐lyases. Seven CAZymes overlapped in the same direction across five cohorts (Table S4, Supporting Information). Three out of seven CAZymes were significantly enriched in IBS in at least one cohort. These CAZymes (GH20, GH28, and GH73) are linked to the degradation of mucin, pectin, and peptidoglycan, respectively (Figure 4b). Likewise, seven metabolic pathways overlapped in the same direction across five cohorts, with three (PWY‐6953: dTDP‐3‐acetamido‐α‐D‐fucose biosynthesis, PPGPPMET‐PWY: ppGpp biosynthesis, ASPASN‐PWY: superpathway of L‐aspartate and L‐asparagine biosynthesis) being significantly enriched in IBS in at least one cohort (Figure 4b).

A recent study reported that bacterial histamine could induce abdominal pain in a mouse model of IBS.^[^ ^37^ ^]^ We compared the microbiome pathways for histamine biosynthesis between IBS and healthy control groups from these five cohorts and found that the genes for eight out of ten related pathways were consistently more abundant in IBS microbiomes than healthy controls, although not reaching statistical significance (FDR > 0.1, Table S4, Supporting Information). Impaired tryptophan metabolism has been observed in subjects with IBS.^[^ ^38^ ^]^ However, we did not find consistent patterns of the gene for the tryptophanase enzyme (EC 4.1.99.1) responsible for indole production across five cohorts when controlling for covariates, despite its significant enrichment in the IBS microbiome in both PRJEB42304 and PRJNA812699 cohorts (*p* < 0.05, Table S4, Supporting Information).

### 2.5 Habitual Dietary Ingredients are Associated with Microbial Signature Species of IBS

**Figure 5:**
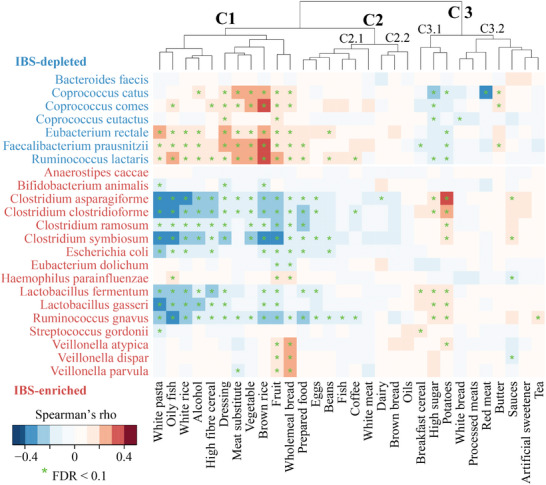
Association between intake levels of food ingredients and microbial signature species for IBS. Hierarchical clustering of the correlation patterns with microbiome was generated with heatmap.2 function using a complete agglomeration method with Euclidean distance. See Figures S7 and S8 (Supporting Information) for associations of food items and micronutrients with microbial signature species, respectively. The color gradient indicates Spearman's correlation coefficient (ρ); * indicates false discovery rate (FDR) < 0.1.

We next sought to identify foods associated with IBS‐enriched and IBS‐depleted features of the gut microbiome by associating the abundance of the identified gut microbiome signatures with dietary ingredients from food frequency questionnaires of healthy subjects in published datasets.^[^ ^6^, ^39^, ^40^ ^]^ Food items that are positively related to IBS‐depleted species and inversely associated with IBS‐enriched species may help to achieve the desired modulation. Using Spearman's rank correlation, we assessed links between food ingredient intake and microbial signature species for IBS. At a broader level, we found that food groups were segregated into three clusters with distinct associations with IBS‐depleted and IBS‐enriched species (**Figure** **5**). Food items in Cluster 1 (including fruit, vegetable, brown & white rice, wholemeal bread, high fiber cereal, white pasta, meat substitute, dressing, oily fish, and alcohol) were negatively related to IBS‐enriched species but positively associated with IBS‐depleted species, despite significant positive associations of wholemeal bread and fruit with *Veillonella* spp. and *Haemophilus parainfluenzae* (IBS‐enriched species). The associations between food items in Cluster 2.1, such as prepared foods (including pizza, lasagne, quiche, vegetable soups tinned, potato salad, and savory pies), eggs, beans, fish, and white meat, and IBS‐associated species were similar to those in Cluster 1, despite many associations being insignificant. In contrast, high sugar, potatoes and breakfast cereal in Cluster 3.1 were negatively related to IBS‐depleted species and positively associated with IBS‐enriched species (Figure 5). When focusing on IBS subjects (n = 78,^[^ ^6^ ^]^), many associations exhibited the same directionality as healthy controls, although the majority were not statistically significant (Figure S6, Supporting Information). We found similar divergence of specific foods in Clusters 1–3 and Cluster 4 (Figure S7, Supporting Information). Clinical trials have shown that low FODMAP (fermentable oligosaccharides, disaccharides and monosaccharides, and polyols) diets may help improve symptoms of IBS.^[^ ^41^, ^42^ ^]^ Among the foods that were linked to a lower abundance of IBS‐enriched species and a higher abundance of IBS‐depleted species, many overlap with the low FODMAP foods based on two sources (^[^ ^43^ ^]^ and www.monashfodmap.com), but several (e.g., mushroom, onions, apples, grapefruit, peaches & plums, pears, dried fruit raisins, muesli, sources tomato & white yellow green pasta, and wholemeal & crisp bread) are formally classified as high FODMAP foods (Figure S7, Supporting Information). Furthermore, several foods were negatively linked with IBS‐depleted species and positively associated with IBS‐enriched species (e.g., parsnips & turnips, mashed potatoes, sauces white cheese, lamb roast, lite butter, and carrots) are low FODMAP foods (Figure S7, Supporting Information). Particularly, mashed potato was strongly associated with *Ruminococcus gnavus* that plays a pathogenic role in IBS.^[^ ^44^ ^]^ Among the micronutrients in Cluster 1, minerals (e.g., selenium, zinc, potassium, magnesium), vitamins (e.g., biotin (vitamin B7), vitamin B12, vitamin C, vitamin K1), and alcohol, were positively correlated with IBS‐depleted species and negatively associated with IBS‐enriched species, although in some cases, positive links were seen with *Veillonella* spp. and *Haemophilus parainfluenzae* (Figure S8, Supporting Information).

We further investigated if there was a difference in the alcohol intake levels between IBS subjects and healthy controls in four cohorts for which habitual dietary intake data was available. Among the two cohorts with raw food frequency profiles, alcohol consumption was significantly lower in subjects with IBS relative to healthy controls in both cohorts (Wilcoxon rank‐sum test, *p* < 0.05, Figure S9a,b, Supporting Information). For the two cohorts with only binary information on alcohol drinking, a higher but not statistically significant alcohol drinking frequency was found in healthy controls than in IBS subjects in both cohorts (Fisher's exact test, *p* > 0.1, Figure S9c,d, Supporting Information), although a lower alcohol drinking frequency was found in healthy controls than in IBS subjects in one of the two cohorts (i.e., PRJEB11419) when considering only IBS subjects without any other diseases (*p* > 0.1, Figure S9e, Supporting Information).

The diets that are associated with a lower abundance of IBS‐enriched features and a higher abundance of IBS‐depleted features include fruit (apples, bananas, dried fruit raisins, fruit squash, grapefruit, grapes, melon, oranges mandarins, peaches plums, pears, strawberry, raspberry, kiwi, sweetcorn), vegetable (broccoli spring greens, cucumber celery, green salad lettuce, mushroom, onions, sweet peppers, tomatoes), rice (brown rice, white rice), bread (crisp bread, wholemeal bread), high fiber cereal (high fiber bran, muesli), pasta (lasagne meat, quiche, sauces tomato pasta, white yellow green pasta), meat substitute (tofu soya meat), dressing (other salad dressing, peanut butter, pickles chutney, sauces curry), oily fish (oily fish, shell fish, white fish), alcohol (beer lager cider, spirits, wine), and other foods (e.g., buns pastries croissants, cream crackers, chocolates, dried lentils beans, eggs, pancakes muffins oatcakes, pizza, probiotic yoghurts, semi skimmed milk). Foods that were negatively correlated with the abundance of taxa that are desirable for increased abundance in subjects with IBS include high sugar (e.g., marmalade honey syrup, milk puddings, plain biscuit), mashed potatoes (usually with added cream, butter, milk), breakfast cereal (e.g., non‐ready to eat porridge), sauces white cheese, lamb roast, and parsnips & turnips (belong to root vegetables). Identifying these microbiota‐targeting food ingredients serves as a foundation for trials seeking dietary modifications in treating IBS.

### 2.6 Rifaximin Therapy for the Modulation of Gut Microbiota in Subjects with IBS‐D

**Figure 6:**
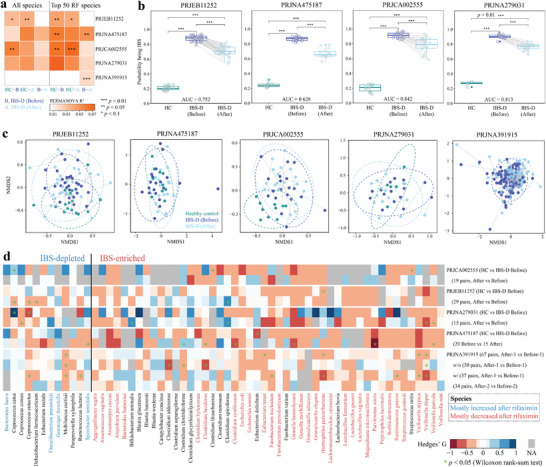
Modulation of altered gut microbiota of IBS‐D subjects by rifaximin. a) When focusing on the top 50 most important RF features, variation of the gut microbiome is reflective of health state and rifaximin administration; the color gradient indicates microbiome variations (Bray‐Curtis dissimilarity); ***p < 0.01, **p < 0.05, *p < 0.1. b) Rifaximin administration reduced the probability (0–1) of being classified as the IBS‐D group determined using random forest (RF) classification with classifiers trained on species‐level abundances of healthy controls and IBS‐D before rifaximin administration (***p < 0.01, Wilcoxon rank‐sum test); AUC indicates area under the curve that represents the prediction accuracy of RF training model for each cohort. c) Non‐metric multidimensional scaling (NMDS) of the top 50 most important differentiating species (Bray‐Curtis dissimilarity, permutations = 999). d) The shift of 60 identified microbial signature species in response to rifaximin administration; the color gradient indicates effect size; *p < 0.05 (Wilcoxon rank‐sum test). w/o indicates without symptom relapse; w/ indicates with symptom relapse.

Rifaximin is efficacious in managing IBS‐D, although the effect is generally transient,^[^ ^7^, ^21^, ^22^ ^]^ and its likely primary mechanism of altering the gut microbiota is largely underexplored.^[^ ^45^ ^]^ We assessed the gut microbial community composition of IBS‐D subjects who were treated with rifaximin, and corresponding healthy controls, from five studies (Table S1, Supporting Information). Among the four cohorts with both HC and IBS‐D, only one study (PRJCA002555)^[^ ^46^ ^]^ showed a significant difference in the overall gut microbiome composition between HC and IBS‐D subjects before rifaximin administration (*p* < 0.05, PERMANOVA, Bray‐Curtis dissimilarity, **Figure** **6**a). However, two additional studies (PRJEB11252^[^ ^7^ ^]^ and PRJNA475187^[^ ^9^ ^]^) became significant while considering the top 50 most important RF features that distinguished IBS‐D before rifaximin administration from HC (Figure 6a–c). Likewise, rifaximin administration induced significant microbiome changes in two studies (PRJNA475187^[^ ^9^ ^]^ and PRJNA391915^[^ ^21^ ^]^) when considering only the top 50 RF features rather than all species (*p* < 0.05, PERMANOVA, Bray‐Curtis dissimilarity, Figure 6a). Furthermore, machine learning demonstrated that rifaximin administration consistently shifted the gut microbiota towards a decreased probability of being IBS‐D (*p* < 0.01, Wilcoxon rank‐sum test, Figure 6b, Methods), even though it did not induce statistically significant changes in the overall gut microbial community composition (Figure 6a). Eight out of the top 50 RF features overlapped with the 60 REM signature species identified, including six IBS‐enriched species (*Clostridium clostridioforme*, *Clostridium glycyrrhizinilyticum*, *Escherichia coli*, *Eubacterium dolichum*, *Haemophilus parainfluenzae*, and *Ruminococcus gnavus*) and two IBS‐depleted species (*Coprococcus catus* and *Faecalibacterium prausnitzii*) in at least two of the four investigated cohorts in Figure 6b (Table S5, Supporting Information).

To examine the dynamics of the 60 IBS‐related microbial signature species identified above in the current study for their response to rifaximin administration, we estimated their effect size between “IBS‐D After” and “IBS‐D Before”. Of these signature species, four out of the 12 IBS‐depleted species were mostly increased (i.e., more than half of cohorts with the signature species detected) following rifaximin administration, while 32 out of the 48 IBS‐enriched species were mostly decreased (i.e., more than half of cohorts with the signature species detected) following rifaximin administration, although mostly insignificantly (Figure 6d). Overall, 60% of the signature species associated with IBS shifted towards a status more similar to HC in response to rifaximin administration. However, only a few differed significantly in particular cohorts, warranting further investigation.

## 3 Discussion

In this metanalysis, we have identified robust microbiome signatures of IBS, and distinct microecological changes reflecting outgrowth of dysbiosis anaerobes and oral microbes. Our major findings and their ramifications are:

### 3.1 Depleted SCFA Producers in IBS

Growing evidence indicates the role of gut microbiota in IBS though it is not clear if this relationship is causative, consequential, or symptom exacerbating. Various gut microbiome‐targeted interventions have produced inconsistent results, probably due to the clinical heterogeneity of IBS and lack of microbiome signatures,^[^ ^47^ ^]^ hence the current study. Intriguingly, we observed that IBS‐depleted species are primarily SCFA‐producing bacteria (Figure 3a; Table S3, Supporting Information). A growing body of evidence suggests that altered levels of SCFAs may affect the pathogenesis of IBS, with conflicting results.^[^ ^48^ ^]^ Our current data support a role for depletion of SCFA‐producing bacteria in IBS, contradicting our previous finding that excessive acid production by fermentative bacteria led to lower luminal pH that was perceived as pain in sensitized individuals.^[^ ^6^, ^49^ ^]^ This discrepancy may be due to the current study being based on a much greater number of subjects and much greater diversity of diet, and lifestyle, from a huge diversity of locations, whereas our previous studies were based in either of two western European cities with homogeneous populations and similar habitual diets.

### 3.2 Oral‐Gut Transmission and Gut Homeostasis in IBS

Two clusters of signature species differing in aerotolerance were enriched in IBS subjects, and the facultative anaerobic signature taxa in M2 are typically found in the human oral cavity. Translocation of oral bacteria to the gut has been described in healthy individuals but increased in patients with colorectal cancer and rheumatoid arthritis.^[^ ^50^ ^]^ Most of these “gut‐traveling” oral microbes in M2 are opportunistic pathogens (Figure 3a; Table S3, Supporting Information), which could plausibly trigger inflammation and thus contribute to the pathogenesis or perpetuation of IBS.^[^ ^51^ ^]^ Oral‐gut transmission could alter the gut microbiota with pathophysiological consequences.^[^ ^50^, ^52^ ^]^ The subsequent colonization by translocated oral microbes in the gut may be associated with the disturbance of gut homeostasis. *Veillonella* is positively correlated with smoking and dental calculus.^[^ ^53^ ^]^ Four *Veillonella* species (*V*. atypica, *V*. dispar, *V. parvula*, and *V. ratti*) were enriched in IBS (Figure 3a), in line with previous findings.^[^ ^54^, ^55^, ^56^ ^]^ Our results suggest another reason for promoting oral hygiene as community healthcare policy for risk‐reduction for IBS, and other diseases with so‐called “dysbiosis” involving oral microbes.

### 3.3 Potential Dietary Modulation of Gut Microbiota for Managing IBS

The consumption of specific foods has been strongly associated with IBS‐related symptoms,^[^ ^57^, ^58^ ^]^ and dietary management is the single most preferred therapy for treating IBS.^[^ ^59^ ^]^ Various types of dietary modifications, e.g., traditional dietary advice, low FODMAP diet, and gluten‐free diet, have been investigated, with varying degrees of effectiveness,^[^ ^60^, ^61^, ^62^ ^]^ probably due to the heterogeneity of IBS and the lack of microbial signatures^[^ ^47^ ^]^ that would justify a uniform dietary approach. Thus, the identification of microbiota‐based food response would be valuable.^[^ ^63^ ^]^

We found microbiota‐targeted dietary patterns, providing a basis for designing dietary interventions in modulating gut microbiome. The differences we identified in IBS‐microbiome functional potential further supports the food patterns. For example, the potential capacity to degrade polysaccharides was depleted in the gut microbiome of IBS subjects, consistent with fruits and vegetables being linked a higher abundance of IBS‐depleted bacteria and a lower abundance of IBS‐enriched bacteria. These results are generally in line with reported dietary patterns that have the potential to reduce gut inflammation via the gut microbiota.^[^ ^64^ ^]^ Furthermore, our data revealed inconsistencies between the foods categorized based on IBS‐associated microbial signatures and the FODMAP food group, possibly explaining the variable effectiveness of low‐FODMAP dietary intervention in IBS subjects.^[^ ^42^, ^62^ ^]^ Intriguingly, we found that alcohol consumption was associated with a greater abundance of IBS‐depleted bacteria and a lower abundance of IBS‐enriched bacteria. Additionally, alcohol consumption was reduced in IBS subjects in the four cohorts studied (Figure S9, Supporting Information). Alcohol intake was associated with higher gut microbiota diversity,^[^ ^65^, ^66^ ^]^ and alcohol consumption was significantly reduced in IBS subjects in two out of five studied cohorts (Figure S8, Supporting Information). Previous studies demonstrate that alcohol consumption was not associated with IBS.^[^ ^67^, ^68^, ^69^, ^70^ ^]^ In contrast, other studies implicated that alcohol consumption was associated with IBS.^[^ ^71^, ^72^, ^73^ ^]^ Heavy drinking was found to be associated with next‐day gastrointestinal symptoms in IBS subjects, despite no significant difference in alcohol consumption patterns between IBS subjects and healthy controls.^[^ ^74^ ^]^ The role of alcohol in IBS remains unclear, warranting further investigation.

Surprisingly, the root vegetables mashed potatoes, parsnips, and turnips were linked with a higher abundance of IBS‐enriched species. Recently, potato consumption was reported to be linked with elevated gut inflammatory markers.^[^ ^64^ ^]^ Parsnip contains high levels of furocoumarins, which can inflict irreversible inactivation of the cytochrome P450 3A4 enzyme (CYP3A4) in the small intestine, where it serves as a barrier against xenobiotics.^[^ ^75^ ^]^ Additionally, sauces, white cheese, and roast lamb are high in fat, and high dietary intake of fat has been found to adversely impact microbiota richness and diversity.^[^ ^76^ ^]^ Interestingly, cauliflower and cabbage were positively associated with both IBS‐enriched and ‐depleted species, although with low numbers of significant associations (Figure S7, Supporting Information). In addition to being good sources of fiber, cauliflower, and savoy/ fermented cabbage are high‐FODMAP foods, while common cabbage is a low‐FODMAP food.

### 3.4 Rifaximin may Improve IBS Symptoms by Suppressing Oralisation of the gut Microbiome

Exploiting the availability of 404 gut microbiomes from five antibiotic‐therapy studies, we found that the gut microbiota shifted towards a significantly decreased probability of being classified by machine learning models as IBS‐D status following rifaximin administration, and most IBS‐related signature species did not shift significantly, although 60% of signature species for IBS shifted towards a status more similar to those HC upon treatment (Figure 6). Nonetheless, the IBS‐enriched species that further increased in abundance following rifaximin administration were primarily obligate anaerobes in network M1. It appears that rifaximin is likely to repress the abundance of facultative anaerobic but not obligate pathobionts, possibly accounting for the relapse of IBS‐D after rifaximin administration. However, the shift in most signature species was not statistically significant, and further investigation is warranted to determine the impact of rifaximin on facultative anaerobic pathobionts in clinical trials. One recent study has suggested that rifaximin suppressed oralisation of the gut microbiome and attenuated systemic inflammation.^[^ ^77^ ^]^ The imbalanced redox status in IBS may promote the growth of these facultative anaerobic pathobionts. Nevertheless, although many signature species shifted unexpectedly in response to rifaximin administration, the identified dietary patterns could complement the modulation of some important signature species, e.g., *Coprococcus spp*., *Eubacterium rectale*, *Ruminococcus lactaris*, *Clostridium spp*., *Bifidobacterium animalis*.

### 3.5 Limitations

The current study was limited by combining case‐control cohorts that are heterogeneous for geography, ethnicity, diagnosis criteria for IBS, and methodological detail (e.g., DNA extraction method, primers and region of 16S rRNA gene, sequencing platform) (Table S1, Supporting Information). Given the heterogeneity and the small sample size of some cohorts, we applied a random‐effects model to identify the microbial signatures associated with IBS. Although SPINGO provides the highest accuracy at the species level compared to other tools and the metagenomic species is strongly associated with 16S species (Figure S4d, Supporting Information) or the REM estimates in two shotgun metagenomic cohorts (Figure S4a,e, Supporting Information), the annotation of 16S amplicon sequences at the species level is still limited. Despite this, the validation of the microbial signatures in the shotgun metagenomic cohorts supports the microbial signatures based on 16S amplicon sequences. Although the beta‐diversity did not differ significantly between IBS subtypes, it is still possible that individual taxa may differ in abundance between IBS subtypes. A limitation preventing resolution of this question is the paucity of IBS‐subtyped cohorts, and further meta‐analysis is warranted when such data becomes available to evaluate the variation of individual taxa between IBS subtypes across cohorts. Moreover, we could not confirm a causal relationship between the diets and the microbiota as the identified dietary patterns were based on relational diet‐microbiota analysis. There may be variability across three studies; nevertheless, these studies were all carried out in Ireland with a similar methodology. However, these microbiota‐targeting food patterns lay a foundation for trialling rational dietary therapy to manage symptoms of IBS. We also note that differing dosages of rifaximin were prescribed across the cohorts, which may have differentially affected the modulation of gut microbiota. Furthermore, covariates may also explain observed associations, while complete metadata was not available in some cohorts. In case‐control studies, healthy controls are typically matched to cases in order to avoid cofounding, and within the investigated cohorts, age and sex had no significant associations with gut microbiota, except for sex in one cohort. Nevertheless, future studies should make the entire metadata available to ensure all confounders may be tested.

## 4 Conclusions

We undercover reproducible gut microbiome signatures for IBS across cohorts and provide insights on the potential role of SCFA‐producing bacteria, oral‐gut transmission, and imbalance of redox homeostasis in IBS pathogenesis. These microbiome signatures, together with identified microbiota‐targeted dietary patterns, provide targets for future investigation. It appears that rifaximin exerts positive effects in IBS‐D, primarily by modulating the facultative anaerobic pathobionts in the gut. Further studies are warranted that investigate the role of redox homeostasis in the etiology of IBS and the clinical efficiency of dietary intervention.

## 5 Experimental Section

To combine the resources of literature and data availability, the terms “Irritable Bowel Syndrome” OR “IBS” [Title/Abstract] were searched in PubMed and NCBI sequence databases for case‐control studies with publicly available gut 16S rRNA data or shotgun metagenomic data from both IBS and healthy controls in October 2021. Additionally, studies were searched that used rifaximin therapy to treat IBS‐D, irrespective of the availability of healthy controls. After excluding cohorts without metadata demonstrating case or control status for individual samples and cohorts with less than five cases or controls, fourteen case‐control cohorts as discovery cohorts, three cohorts as validation cohorts, and five cohorts related to rifaximin therapy were curated (Table S1, Supporting Information). In January 2024, two new cohorts were studied (shotgun metagenomic PRJEB19857 and metatranscriptomic PRJNA812699) that were publicly available to identify replicable functional signatures. The PRJEB37924 and PRJEB19857 cohorts only included baseline samples. Raw sequences were downloaded from European Bioinformatics Institute (https://www.ebi.ac.uk/), except PRJCA002555, which was obtained from Genome Sequence Archive (https://ngdc.cncb.ac.cn/gsa/). For the AGP cohort, healthy controls and individuals with IBS but without any other comorbidities from the United States, United Kingdom, Australia, and Canada (16S data) and the United States and United Kingdom (shotgun) were included. The samples with shallow sequences (16S < 2000 reads per sample and shotgun metagenomic samples < 0.5 million reads per sample as suggested in previously published studies^[^ ^78^, ^79^ ^]^) were excluded, resulting in a total of 8906 gut microbiome datasets in the case‐control analysis and 404 gut microbiomes in the rifaximin therapy analysis.

### Taxonomic and Functional Annotation

16S rRNA amplicon and shotgun metagenomic sequences were filtered with Trim Galore using default settings under the “–paired” option for paired sequences.^[^ ^80^ ^]^ SPINGO is a bootstrapping k‐mers‐based algorithm, that provides highest accuracy at the species level compared to other tools.^[^ ^27^, ^81^, ^82^ ^]^ Thus, the filtered 16S rRNA sequences were then annotated using SPINGO (v1.3) for species‐level taxonomic profiling against the RDP database (default setting), with paired‐end sequences being concatenated. In the AGP cohort, both 16S rRNA sequences were annotated, with and without the removal of the 20 blooming sequences.^[^ ^33^ ^]^ The quality‐filtered shotgun metagenomic sequences were further mapped to the human and PhiX genome database^[^ ^83^ ^]^ to decontaminate human and PhiX sequences with Kraken 2.^[^ ^84^ ^]^ The resulting shotgun metagenomic sequences were annotated with bioBakery 3 platform,^[^ ^28^ ^]^ i.e., MetaPhlAn 3 for species‐level taxonomic profiling and HUMAnN 3 for functional profiling with the database mpa_v30_CHOCOPhlAn_201 901 based on UniRef90s gene families. These gene family profiles were further regrouped to three aspects of function, i.e., microbial metabolic pathways (Pathways), carbohydrate active enzyme (CAZyme), and enzyme commission number (EC Number). The CAZyme mapping database (https://github.com/junhuili/cazy_uniref90_humann/blob/main/map_cazy_uniref90.txt)^[^ ^85^ ^]^ was used to regroup the UniRef90s gene families.

### Statistical Analyses

Non‐metric multidimensional scaling (NMDS) ordinations were generated with Bray‐Curtis dissimilarity based on species‐level relative abundance using vegan^[^ ^86^ ^]^ package in R 3.6.2. The compositional difference between IBS and HC was assessed by permutational multivariate analysis of variance (PERMANOVA) with covariate adjusted and marginal sums of squares, via the adonis2 function in vegan:^[^ ^86^ ^]^ adonis2(relative abundance matrix ∼ Health status + Cohort + Age + Sex, permutations = 999, method = “bray”, by = “margin”). The abundance differences of species between IBS and healthy controls were evaluated using Wilcoxon rank‐sum test. The functional composition differences, including CAZyme, EC Number, and Pathways, between IBS and HC were assessed through PERMANOVA with available covariates adjusted and marginal sums of squares based on weighted Bray‐Curtis dissimilarity or unweighted binary Jaccard dissimilarity. PRJEB42304: adonis2(relative abundance ∼ Health status + Age + Sex, permutations = 999, method = “bray”, by = “margin”); PRJEB37924: adonis2(relative abundance ∼ Health status + Age + Sex + BMI + Antibiotics + Time, permutations = 999, method = “bray”, by = “margin”); and AGP: adonis2 (relative abundance ∼ Health status + Country + Ethnicity + Gender + Age + BMI + Antibiotic exposure within 6 months, permutations = 999, method = “bray”, by = “margin”). A regression model, adjusted for covariates (as shown above in the adonis2 model), was utilized to examine the difference in functional abundance between IBS subjects and HC for each shotgun metagenomic cohort. A function was considered to be differentially abundant between IBS and HC if it was significantly enriched or depleted in at least one cohort (FDR < 0.1 or *p* < 0.05) and in the same direction across all five cohorts.

### Random‐Effects Meta‐Analyses for Bacterial Signature Taxa

To identify consistent bacterial signature taxa, we assessed the Hedges’ G value (i.e., a measure of effect size that estimates how one group differs from another) between healthy control and IBS for each species within each discovery cohort using escalc function in the metaphor^[^ ^87^ ^]^ R package. To increase the signal‐to‐noise ratio, taxa were removed that did not show a consistent association with IBS status across studies. Based on the threshold of Hedges’ G > 0.1 for “small effect”,^[^ ^88^ ^]^ the species that were mostly depleted in IBS (i.e., species with Hedges’ G > 0.1 in at least 7 studies and < −0.1 in at most 3 studies, n = 25) and primarily more abundant in IBS (i.e., species with Hedges’ G < −0.1 in at least 7 studies and > 0.1 in at most 3 studies, n = 85) across 14 discovery cohorts were then selected. These two groups of taxa that showed reasonably consistent trends were then subjected to robust statistical testing using a meta‐analysis approach, as described below. The rma function in the metaphor^[^ ^87^ ^]^ R package was next used to fit the random‐effects models of the selected species. Among the 110 selected species, 62 species were found with a false discovery rate (FDR) below 0.1 using Benjamini‐Hochberg correction^[^ ^89^ ^]^ in the random‐effects model. The majority of these species have a p‐value less than 0.05, except two species that were excluded. This approach identified 60 significant species.

### Co‐Occurrence Network Building

To correct for compositional bias, the co‐occurrence network was constructed from SparCC^[^ ^35^ ^]^ correlation coefficients based on the centered log‐ratio (CLR) transformed read count of 60 microbial signature species from the microbiomes of the discovery cohorts. SparCC correlation coefficients and p‐values were estimated and bootstrapped using SpiecEasi (https://github.com/zdk123/SpiecEasi). The resulting values were then transformed to GML format for network visualization using R‐igraph (https://r.igraph.org/). A cut‐off value of FDR < 0.01 was used to generate statistically robust correlations. Gephi v0.9.7^[^ ^90^ ^]^ (modularity function in Blondel's algorithm: resolution = 1.0, randomize, use weights) was used to visualize the co‐occurrence network, which enabled clustering of microbial signature species into three groups with a total of 742 edges. The correlation matrix for all species can be found at https://github.com/junhuili/IBS_co‐occurrence_network.

### Random Forest Classifiers

The training and evaluation of Random Forest (RF) classification model were performed using randomForest R package,^[^ ^91^ ^]^ with varying mtry (from 6 to 60, increment = 2) and ntree (100, 200, 300, 400, 500, 600, 800, 1000, and 2000) parameters to optimize the model. To build microbiome classifiers to differentiate IBS from healthy controls, we performed the cross‐cohort and leave‐one‐out cross‐validations on the 16S datasets using the relative abundance of 60 microbial signature species and calculated the prediction accuracy quantified as area under the curve (AUC) for each validation. *Cross‐cohort cross‐validation*: Classifiers were trained on each cohort, and the prediction accuracy was externally estimated on all other cohorts. *Leave‐one‐out cross‐validation*: Classifiers were trained on all but one cohort, and the prediction accuracy was externally estimated on the remaining hold‐out cohort.

Similarly, to evaluate the effect of rifaximin on gut microbial community composition, the probability (0–1) of being classified in the IBS‐D group after rifaximin administration was predicted using trained RF classifiers based on species‐level abundances of healthy controls and IBS‐D before rifaximin administration. A Wilcoxon rank‐sum test was used to test the difference of prediction probability before and after rifaximin administration.

### Diet‐Microbiome Associations

To identify potentially healthy microbiome‐associated foods, we used Spearman's rank correlation to associate the gut abundance of the identified microbial signature species with habitual diet patterns of healthy individuals in previously published studies.^[^ ^6^, ^39^, ^40^ ^]^ Habitual dietary intakes were assessed using a semi‐quantitative food frequency questionnaire (FFQ), weighted by ten consumptive frequencies according to the method used by Claesson et al.^[^ ^92^ ^]^ The European food classification system was used to evaluate the food groups and McCance and Widdowson's Composition of Foods (seventh) was used to generate nutrient profiles^[^ ^93^ ^]^ from FFQ, respectively. Among the identified microbial signature species for IBS, 23 were detected in the published datasets. The Spearman association with FDR < 0.1 was considered significant.

## Conflict of Interest

The authors declare no conflict of interest.

## Author Contributions

J.L. performed conceptualization (Equal), data curation (Lead), formal analysis (Lead), investigation (Lead), methodology (Lead), visualization (Lead), writing – original draft (Lead), and writing – review & editing (Equal). T.S.G. performed conceptualization (Equal), methodology (Equal), and writing – review & editing (Equal). E.A. performed conceptualization (Equal), funding acquisition (Lead), project administration (Equal), and writing – review & editing (Equal). F.S. performed conceptualization (Equal), funding acquisition (Equal), and writing – review & editing (Equal). P.W.O.T. performed conceptualization (Equal), funding acquisition (Equal), project administration (Equal), supervision (Lead), writing – original draft (Equal), and writing – review & editing (Equal).

## Supporting information

Supporting Information

Supporting Information

Supplemental Tables

## Data Availability

The data that support the findings of this study are openly available in European Bioinformatics Institute at https://www.ebi.ac.uk. Please refer to Table S1 for accession numbers associated with individual cohorts included in this meta‐analysis.
