# Supplementary material for: Cross‐Cohort Gut Microbiome Signatures of Irritable Bowel Syndrome Presentation and Treatment
Source: Adv Sci (Weinh). 2024 Sep 7;11(41):2308313. doi: 10.1002/advs.202308313 (PMC11538712; doi:10.1002/advs.202308313)
Supplement: Supplementary file 1 — Supporting Information [file ADVS-11-2308313-s003.docx]

**Cross-Cohort Gut Microbiome Signatures of Irritable Bowel Syndrome Presentation and Treatment**

Junhui Li^1,2^, Tarini Shankar Ghosh^1,2†^, Elke Arendt^1,3^, Fergus Shanahan^1,4^, Paul W. O’Toole^1,2*^

**Supplementary Figure List**

**Figure S1. Microbial variance analysis. (a)** Significant cohort effects across 16S studies. Non-metric multidimensional scaling (NMDS) based on species-level Bray-Curtis dissimilarity of pooled 16S rRNA gene amplicon sequencing samples (n = 1,422) from 13 16S studies (r^2^ = 0.175, *p* < 0.001, PERMANOVA, permutations=999). Colored boxplots on the top and the right represent Bray-Curtis distance by cohort in the first and second ordinations respectively. **(b)** Variance explained by Rome criteria was small across 11 16S studies (n = 1,311) with the known Rome criteria (Rome III and Rome IV). Columns in red indicate values from the model: adonis2(species abundance ~ HC/ IBS + Rome criteria + Paired/ Single + 16S rRNA gene region + Country, permutations=999, method="bray", by="margin"); column in blue indicates value from the model: adonis2(species abundance ~ Cohort, permutations=999, method="bray").

**Figure S2. (a) mostly decreased features in IBS and (b) mostly increased features in IBS across 14 discovery cohorts.** The color gradient indicates effect size (Hedge’s g) for each cohort; * indicates *p* < 0.05 (Wilcoxon rank-sum test).

**Figure S3. The correlation between the effect size of microbial signatures for IBS based on relative abundance and the effect size based on CLR-transformed read count (Spearman) is strong across the discovery cohorts.** CLR, centered log-ratio.

**Figure S4. Validation of microbial signatures for IBS. (**a) The effect size of baseline samples in PRJEB37924 (shotgun) cohort strongly associates with REM estimate (Spearman). (b) Neither the effect size of REM species in American Gut Project (AGP, i.e., PRJEB11419) shotgun dataset (UK or US) nor 16S dataset associates with REM estimate (Spearman); hollow circles in red indicate AGP US samples (n = 176 HC, 6 IBS3), solid circles in blue indicate AGP UK samples (n = 53 HC, 15 IBS3), while hollow triangles in black AGP 16S samples (n = 5830 HC, 493 IBS). (c) After removing the 20 blooming sequences (https://github.com/knightlab-analyses/bloom-analyses/blob/master/data/newbloom.all.fna), the REM species’ effect size in the AGP 16S dataset is not significantly associated with the REM estimate (Spearman), irrespective of diagnosis mode or country (where all indicate samples from the USA, UK, Australia, and Canada); shape indicates diagnosis mode, color indicates country. (d) The effect size of the species detected in both shotgun metagenome and 16S amplicon sequences significantly associates with each other (Spearman) in the PRJEB42304 cohort. (e) The effect size of baseline samples in PRJEB19857 (shotgun) cohort significantly associates with REM estimate (Spearman). (f) The REM species’ effect size in metatranscriptomic (PRJNA812699) cohort is not significantly associated with REM estimate (Spearman).

**Figure S5. Predictive accuracy of the mtry- and ntree-optimized RF model as measured by the AUC for RF classifiers trained on 60 microbial signatures of discovery cohorts for both cohort-to-cohort and leave-one-out cross-validation.** For further details regarding the tuning of mtry and ntree values to optimize the predictive model, please refer to Table S5. AUC, area under the curve; RF, random forest.

**Figure S6. Many associations (64%, 129 out of 202** **significant associations) were consistent in directionality as healthy control (HC), when focusing on IBS subjects (n = 78, Jeffery et al, 2020).**

Among the 73 inconsistent associations, 85% (62) had a correlation coefficient rho < 0.1 in the insignificant association group. Among the 129 consistent associations, 50% (65) consistent associations had a correlation coefficient rho < 0.1 in the insignificant association group. 202 significant associations indicate associations of microbiome signature species with the food items (white pasta, oily fish, white rice, alcohol, high fibre cereal, dressing, meat substitute, vegetable, brown rice, fruit, whole meal bread, prepared food, eggs, beans, fish, coffee, breakfast cereal, high sugar, and potatoes) in either HC or IBS data. Of these, seven were significant in both HC and IBS data, six were significant only in IBS data, and 189 were significant only in HC data.

**Figure S7. Association between intake levels of foods and microbial signatures for IBS**. Cluster (C) was generated with heatmap.2 function using a complete agglomeration method based on Euclidean distance. The color gradient indicates Spearman's correlation coefficient (ρ) for each cohort; * indicates FDR < 0.1. Food in dark green indicates low FODMAP food; Food in purple indicates high FODMAP food; Food in black indicates both low and high FODMAP food subtypes in the food or Unknown FODMAP category.

**Figure S8. Association between intake levels of food micronutrients and microbial signatures for IBS.** Cluster (C) was generated with heatmap.2 function using a complete agglomeration method based on Euclidean distance. The color gradient indicates Spearman's correlation coefficient (ρ) for each cohort; * indicates FDR < 0.1.

**Figure S9. Reduced frequency of alcohol consumption in subjects with IBS relative to healthy controls.** The p-values in Panel A (PRJEB42304) and Panel B (PRJEB37924) were based on Wilcoxon rank-sum test; The p-values in Panel C (PRJNA541572), Panel D (PRJEB11419, IBS subjects with and without other diseases), and Panel E (PRJEB11419, IBS subjects without other diseases) were based on Fisher’s exact test.


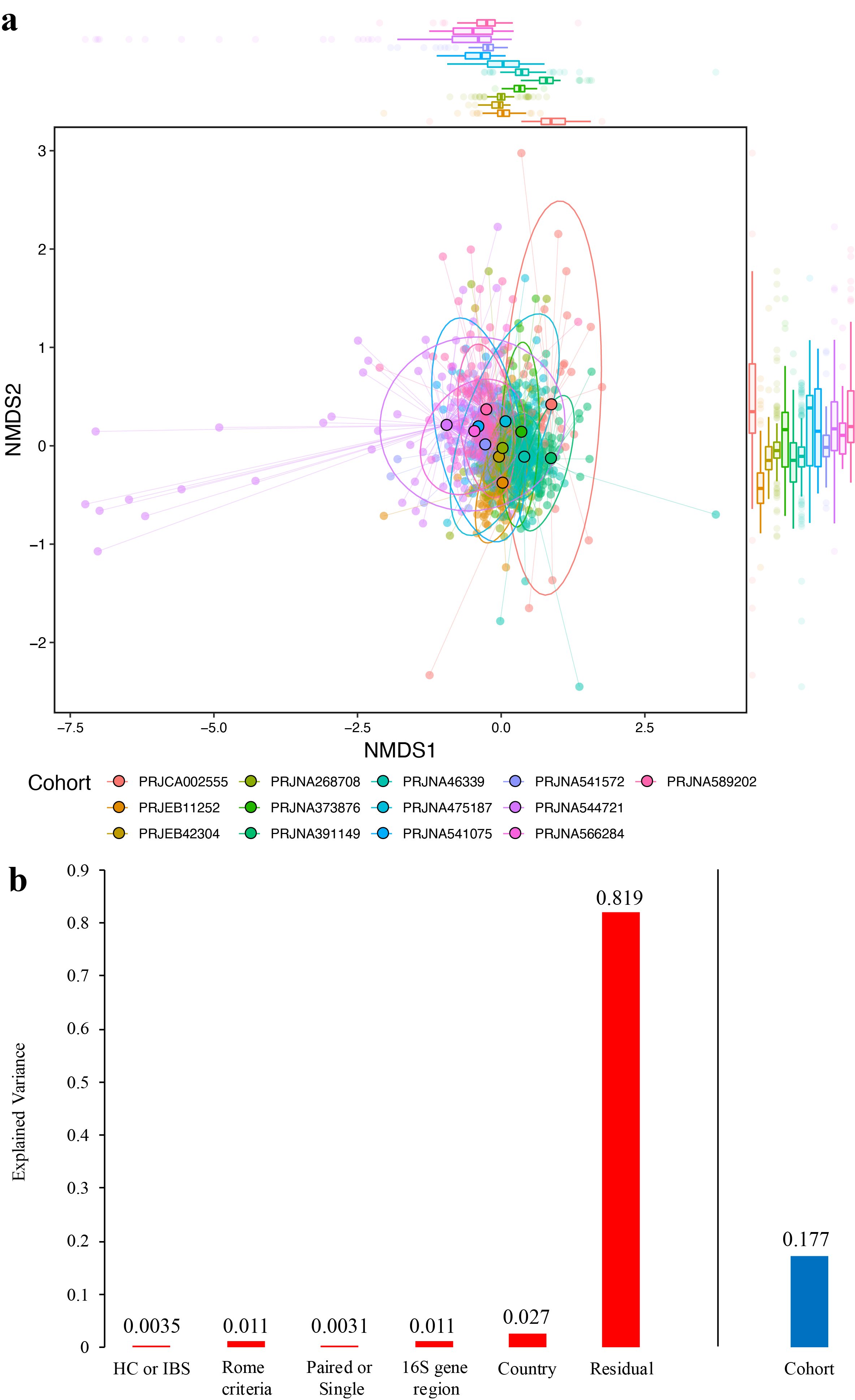


**Figure S1. Microbial variance analysis. (a)** Significant cohort effects across 16S studies. Non-metric multidimensional scaling (NMDS) based on species-level Bray-Curtis dissimilarity of pooled 16S rRNA gene amplicon sequencing samples (n = 1,422) from 13 16S studies (r^2^ = 0.175, *p* < 0.001, PERMANOVA, permutations=999). Colored boxplots on the top and the right represent Bray-Curtis distance by cohort in the first and second ordinations respectively. **(b)** Variance explained by Rome criteria was small across 11 16S studies (n = 1,311) with the known Rome criteria (Rome III and Rome IV). Columns in red indicate values from the model: adonis2(species abundance ~ HC/ IBS + Rome criteria + Paired/ Single + 16S rRNA gene region + Country, permutations=999, method="bray", by="margin"), *p* < 0.001 for each factor; column in blue indicates value from the model: adonis2(species abundance ~ Cohort, permutations=999, method="bray").


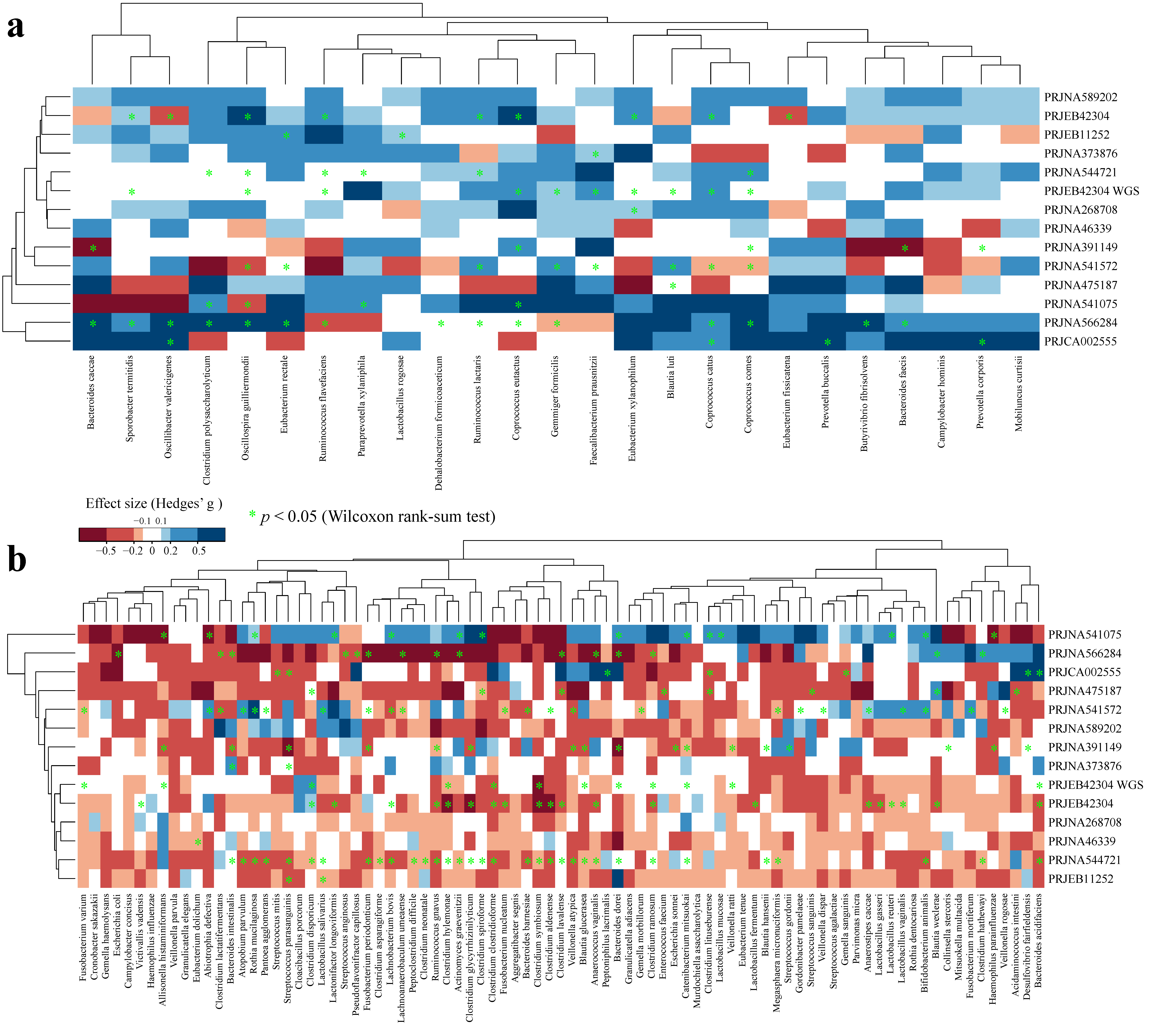


**Figure S2. (a) primarily depleted features in IBS and (b) primarily elevated abundance features in IBS across 14 discovery cohorts.** The color gradient indicates effect size (Hedge’s g) for each cohort; * indicates *p* < 0.05 (Wilcoxon rank-sum test).


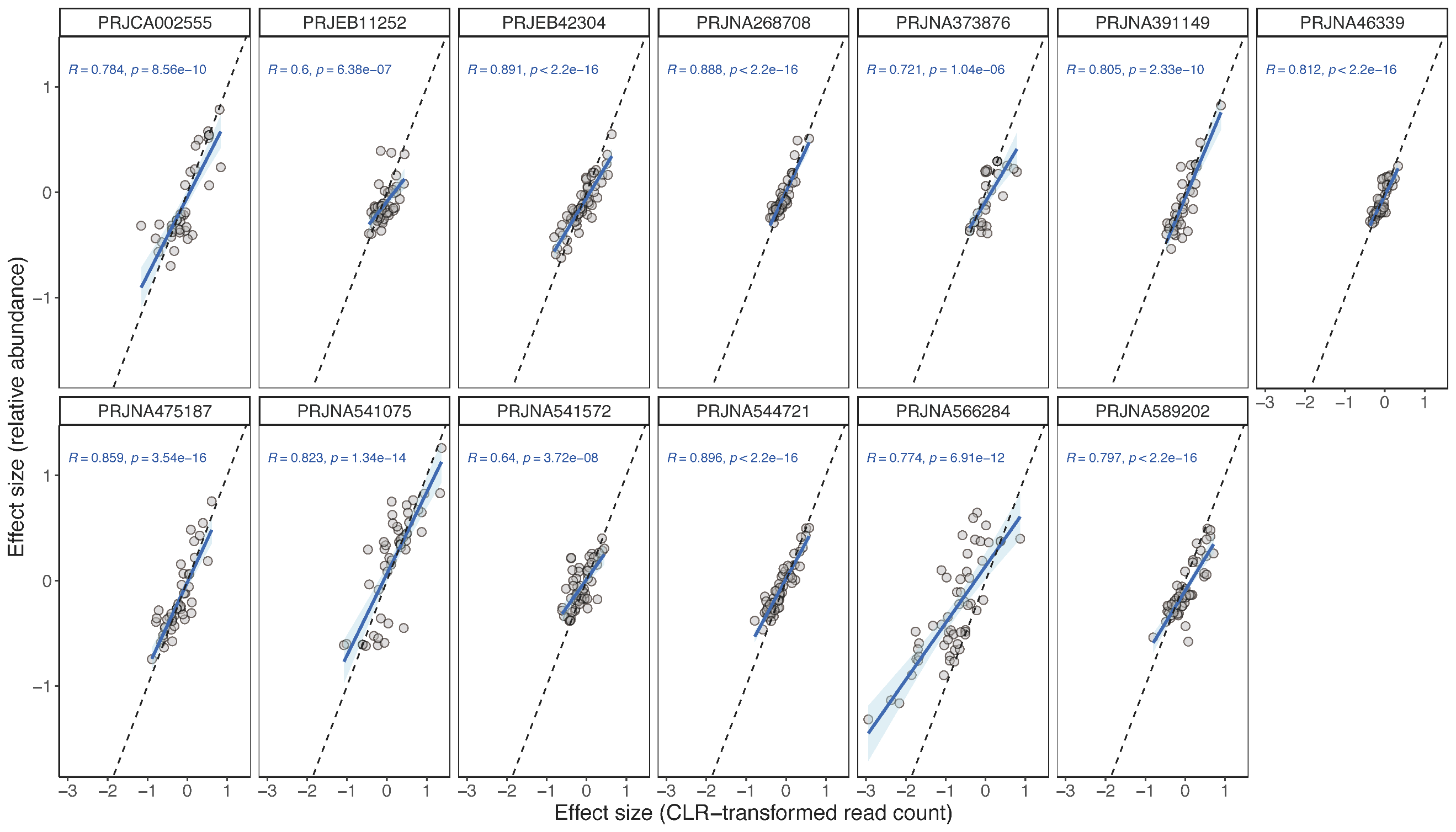


**Figure S3. The correlation between the effect size of microbial signatures for IBS based on relative abundance and the effect size based on CLR-transformed read count (Spearman) is strong across the discovery cohorts.** CLR, centered log-ratio.


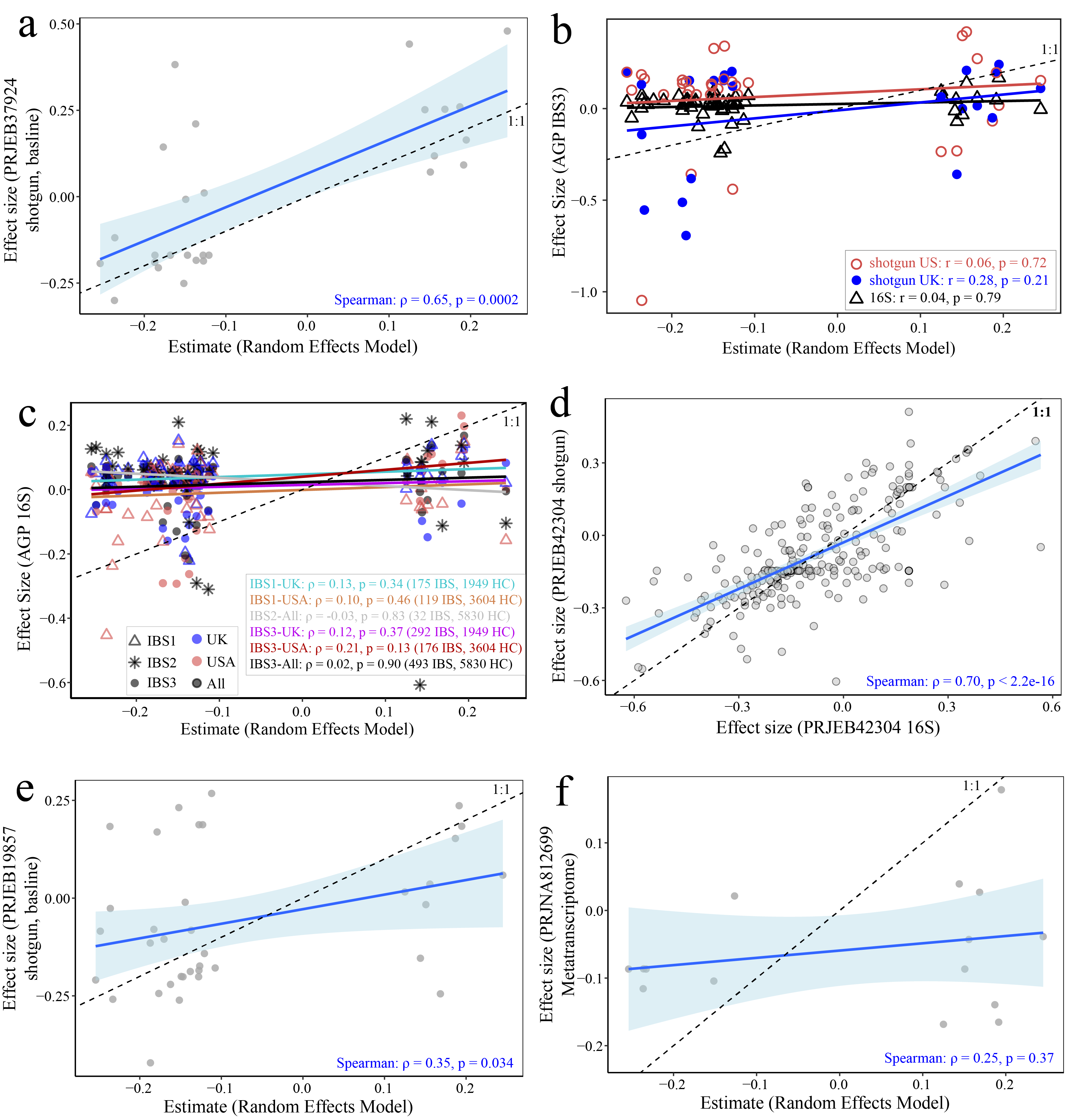


**Figure S4. Validation of microbial signatures for IBS. (**a) The effect size of baseline samples in PRJEB37924 (shotgun) cohort strongly associates with REM estimate (Spearman). (b) Neither the effect size of REM species in American Gut Project (AGP, i.e., PRJEB11419) shotgun dataset (UK or US) nor 16S dataset associates with REM estimate (Spearman); hollow circles in red indicate AGP US samples (n = 176 HC, 6 IBS3), solid circles in blue indicate AGP UK samples (n = 53 HC, 15 IBS3), while hollow triangles in black AGP 16S samples (n = 5830 HC, 493 IBS). (c) After removing the 20 blooming sequences (https://github.com/knightlab-analyses/bloom-analyses/blob/master/data/newbloom.all.fna), the REM species’ effect size in the AGP 16S dataset is not significantly associated with the REM estimate (Spearman), irrespective of diagnosis mode or country (where all indicate samples from the USA, UK, Australia, and Canada); shape indicates diagnosis mode, color indicates country. (d) The effect size of the species detected in both shotgun metagenome and 16S amplicon sequences significantly associates with each other (Spearman) in the PRJEB42304 cohort. (e) The effect size of baseline samples in PRJEB19857 (shotgun) cohort significantly associates with REM estimate (Spearman). (f) The REM species’ effect size in metatranscriptomic (PRJNA812699) cohort is not significantly associated with REM estimate (Spearman).


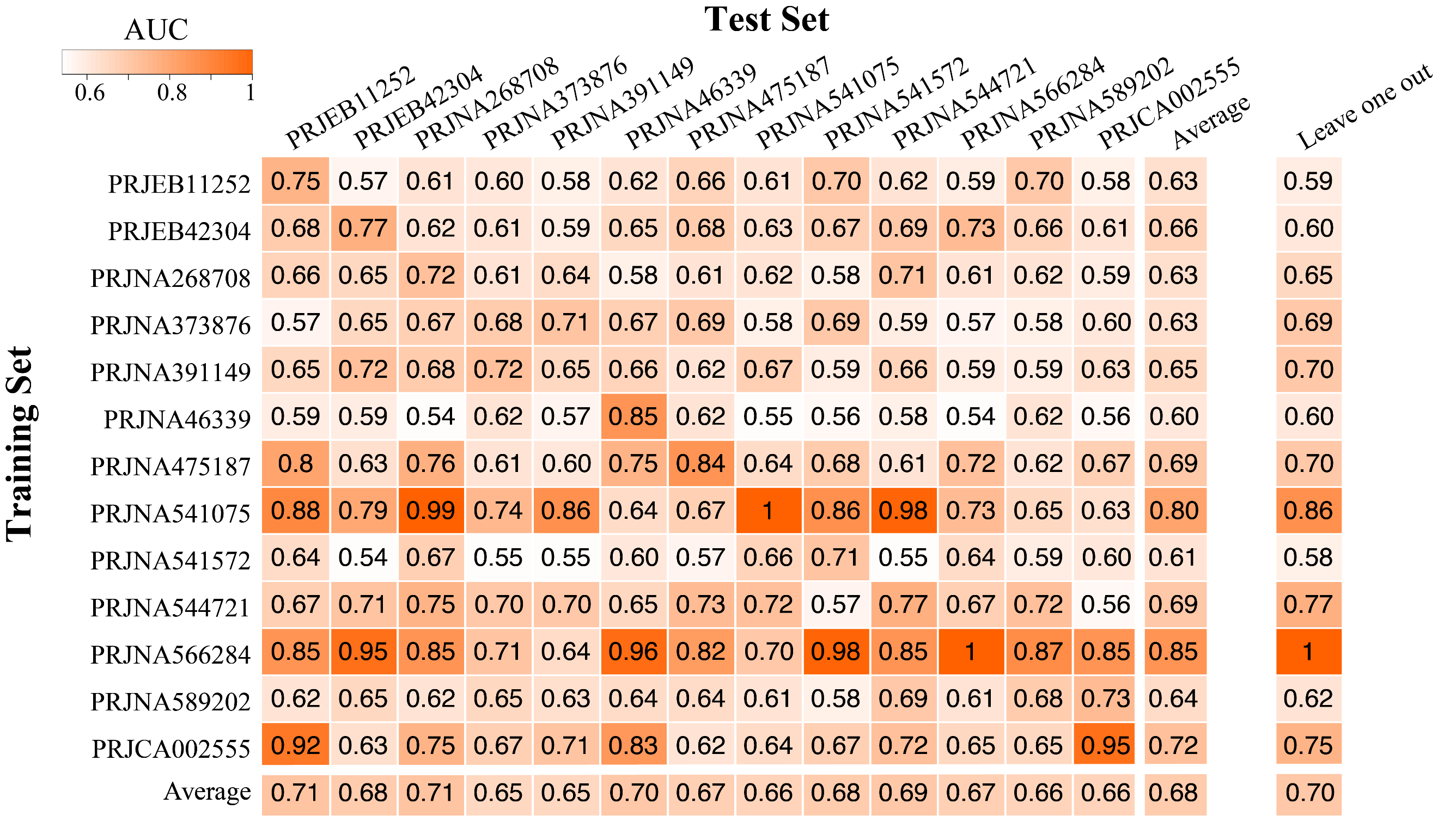


**Figure S5. Predictive accuracy of the mtry- and ntree-optimized RF model as measured by the AUC for RF classifiers trained on 60 microbial signatures of discovery cohorts for both cohort-to-cohort and leave-one-out cross-validation.** For further details regarding the tuning of mtry and ntree values to optimize the predictive model, please refer to Table S5a. AUC, area under the curve; RF, random forest.


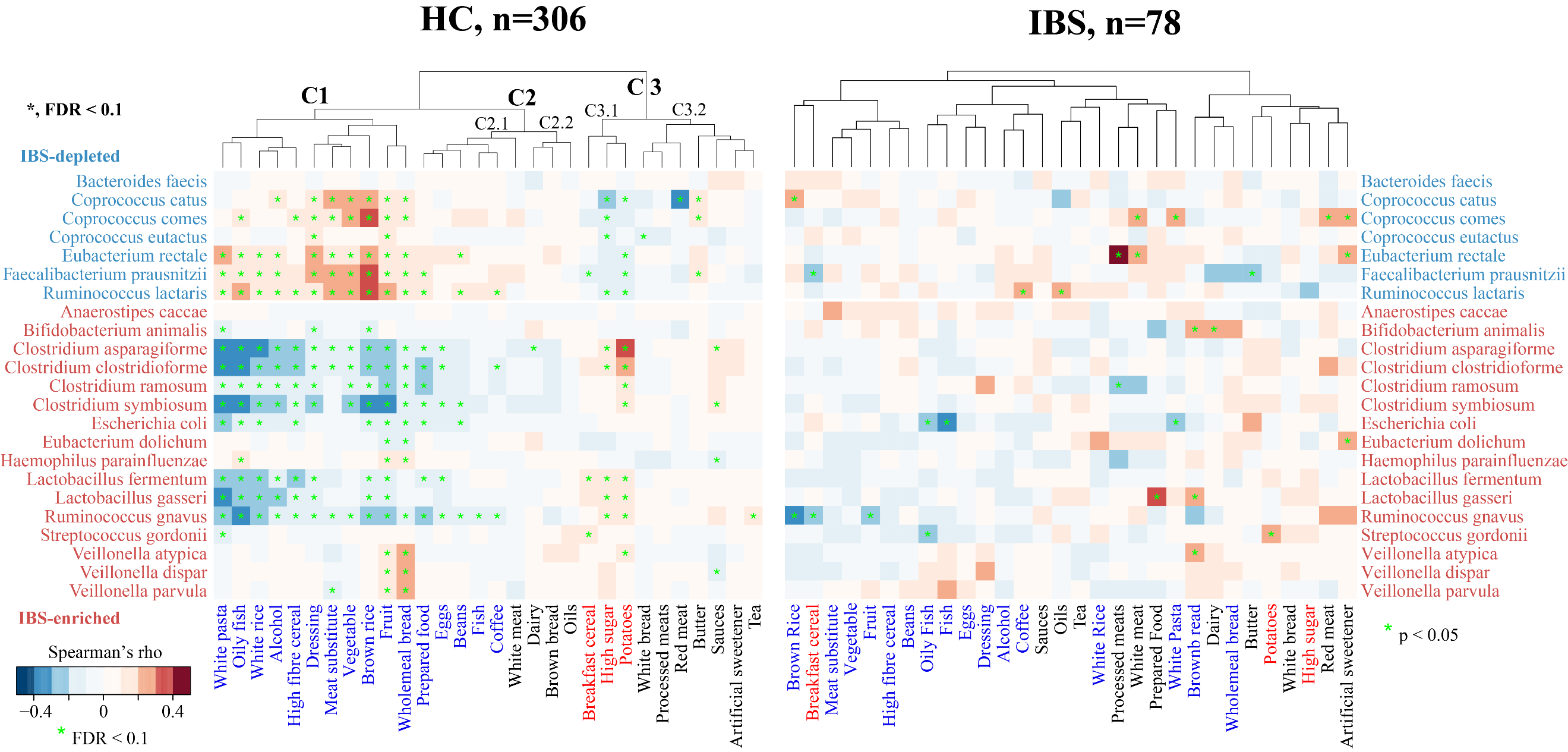


**Figure S6. Many associations (64%, 129 out of 202** **significant associations) were consistent in directionality as healthy control (HC), when focusing on IBS subjects (n = 78, Jeffery et al, 2020).**

Among the 73 inconsistent associations, 85% (62) had a correlation coefficient rho < 0.1 in the insignificant association group. Among the 129 consistent associations, 50% (65) consistent associations had a correlation coefficient rho < 0.1 in the insignificant association group. 202 significant associations indicate associations of microbiome signature species with the food items (white pasta, oily fish, white rice, alcohol, high fibre cereal, dressing, meat substitute, vegetable, brown rice, fruit, whole meal bread, prepared food, eggs, beans, fish, coffee, breakfast cereal, high sugar, and potatoes) in either HC or IBS data. Of these, seven were significant in both HC and IBS data, six were significant only in IBS data, and 189 were significant only in HC data.


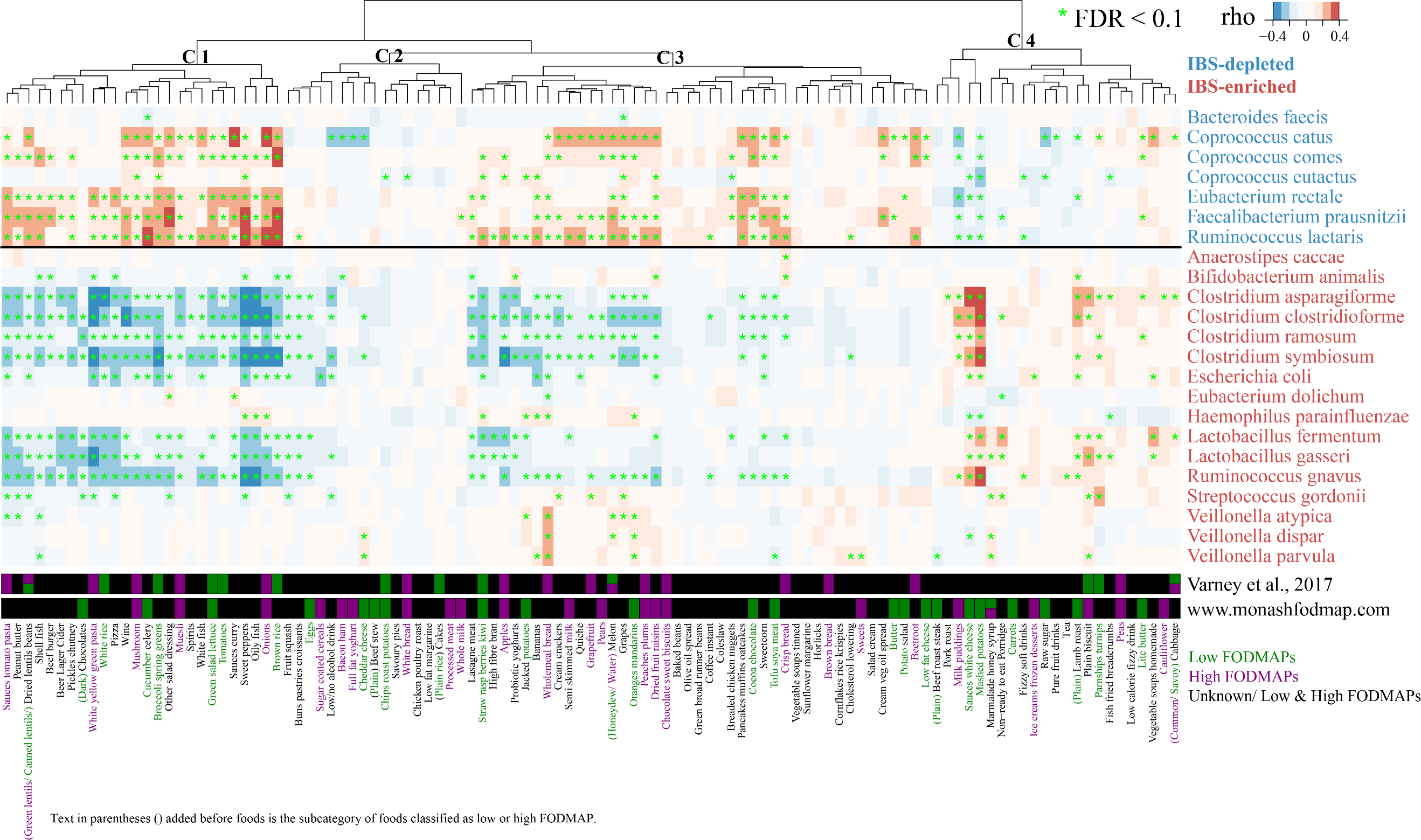


**Figure S7. Association between intake levels of foods and microbial signatures for IBS**. Cluster (C) was generated with heatmap.2 function using a complete agglomeration method based on Euclidean distance. The color gradient indicates Spearman's correlation coefficient (ρ) for each cohort; * indicates FDR < 0.1. Food in dark green indicates low FODMAP food; Food in purple indicates high FODMAP food; Food in black indicates both low and high FODMAP food subtypes in the food or Unknown FODMAP category.

Varney J, Barrett J, Scarlata K, Catsos P, Gibson PR, Muir JG (2017). FODMAPs: food composition, defining cutoff values and international application. *Journal of Gastroenterology and Hepatology* **32:** 53-61.

Alcohol: Beer Lager Cider, Wine, Spirits, Low/no alcohol drink, Low calorie fizzy drink

Artificial sweetener: Low calorie fizzy drink

Beans: Baked beans, Dried lentils beans

Breakfast cereal: Sugar coated cereals, Cornflakes rice krispies, Non−ready to eat Porridge

Butter: Butter, Lite butter

Dairy: Full fat yoghurt, Cheddar cheese, Whole milk, Probiotic yoghurts, Semi skimmed milk, Low fat cheese

Dressing: Other salad dressing, Salad cream

Fish: Shell fish, White fish, Fish fried breadcrumbs

Fruit: Fruit squash, Straw rasp berries kiwi, Apples, Bananas, Grapefruit, Pears, Melon, Grapes, Oranges mandarins, Peaches plums, Dried fruit raisins

High fibre cereal: Muesli, High fibre bran

High sugar: Chocolates, Buns pastries croissants, Cakes, Chocolate sweet biscuits, Pancakes muffins oatcakes, Cocoa chocolate, Horlicks, Sweets, Milk puddings, Marmalade honey syrup, Fizzy soft drinks, Ice creams frozen desserts, Raw sugar, Pure fruit drinks, Plain biscuit, Tofu soya meat

Meat substitute: Tofu soya meat

Oils: Low fat margarine, Olive oil spread, Sunflower margarine, Cholesterol lowering, Cream veg oil spread

Potatoes: Chips roast potatoes, Jacked potatoes, Mashed potatoes

Prepared food: Pizza, Lasagne meat, Quiche, Vegetable soups tinned, Potato salad, Savoury pies

Processed meats: Savoury pies, Processed meat

Red meat: Beef burger, Bacon ham, Beef stew, Beef roast steak, Pork roast, Lamb roast

Sauces: Sauces tomato pasta, Sauces curry, Sauces white cheese

Vegetable: Mushroom, Cucumber celery, Broccoli spring greens, Green salad lettuce, Tomatoes, Sweet peppers, Onions, Green broad runner beans, Coleslaw, Sweetcorn, Beetroot, Carrots, Parnships turnips, Peas, Vegetable soups homemade, Cauliflower, Cabbage

White meat: Breaded chicken nuggets, Chicken poultry roast


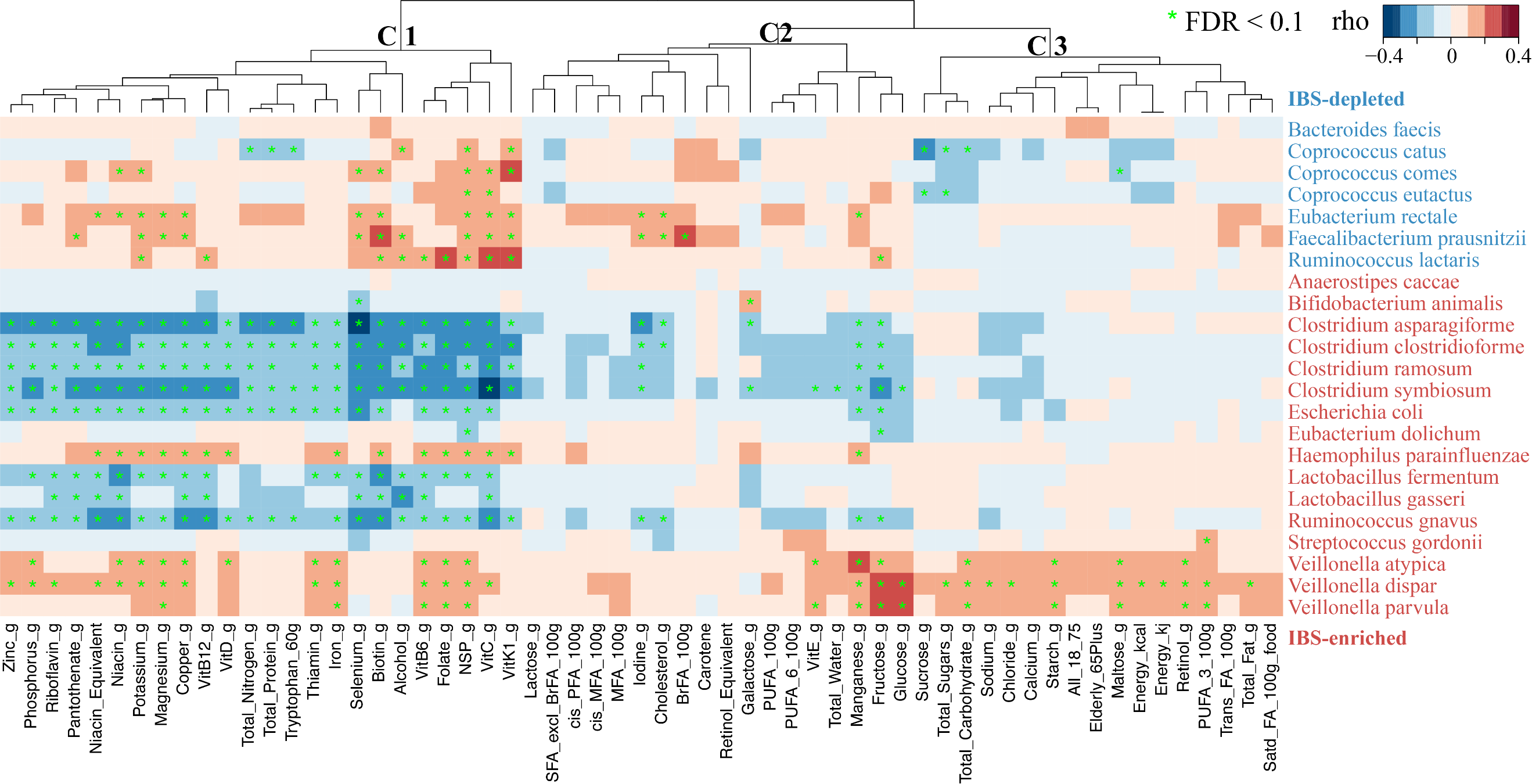


**Figure S8. Association between intake levels of food micronutrients and microbial signatures for IBS.** Cluster (C) was generated with heatmap.2 function using a complete agglomeration method based on Euclidean distance. The color gradient indicates Spearman's correlation coefficient (ρ) for each cohort; * indicates FDR < 0.1.


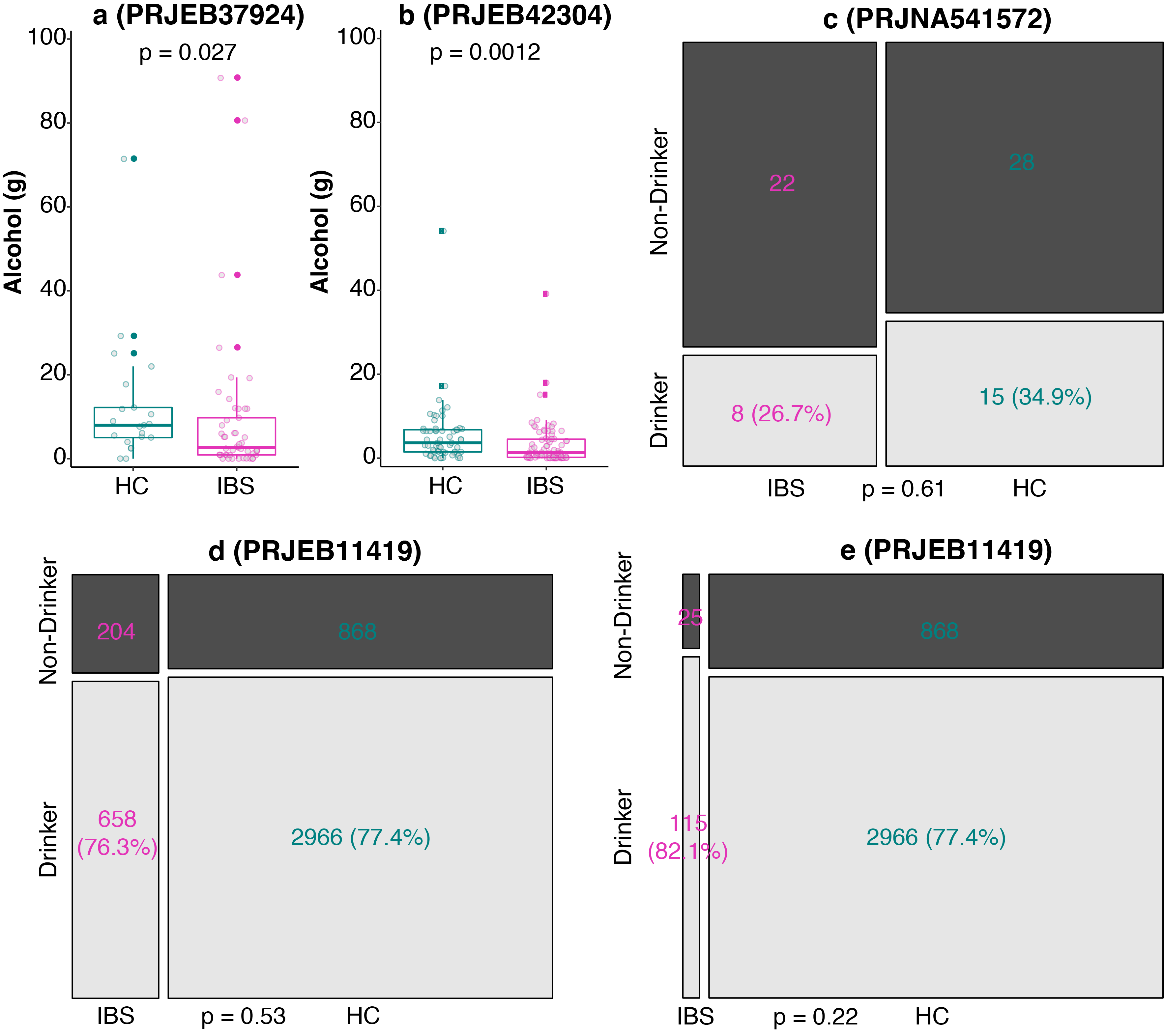


**Figure S9. Reduced frequency of alcohol consumption in subjects with IBS relative to healthy controls.** The p-values in Panel A (PRJEB42304) and Panel B (PRJEB37924) were based on Wilcoxon rank-sum test; The p-values in Panel C (PRJNA541572), Panel D (PRJEB11419, IBS subjects with and without other diseases), and Panel E (PRJEB11419, IBS subjects without other diseases) were based on Fisher’s exact test.
