# Supplementary material for: Cross‐Cohort Gut Microbiome Signatures of Irritable Bowel Syndrome Presentation and Treatment
Source: Adv Sci (Weinh). 2024 Sep 7;11(41):2308313. doi: 10.1002/advs.202308313 (PMC11538712; doi:10.1002/advs.202308313)
Supplement: Supplementary file 2 — Supporting Information [file ADVS-11-2308313-s001.pdf]

ADVANCED SCIENCE
Open Access

Supporting Information

for *Adv. Sci.*, DOI 10.1002/advs.202308313

Cross-Cohort Gut Microbiome Signatures of Irritable Bowel Syndrome Presentation and Treatment

*Junhui Li, Tarini Shankar Ghosh, Elke Arendt, Fergus Shanahan and Paul W. O'Toole**

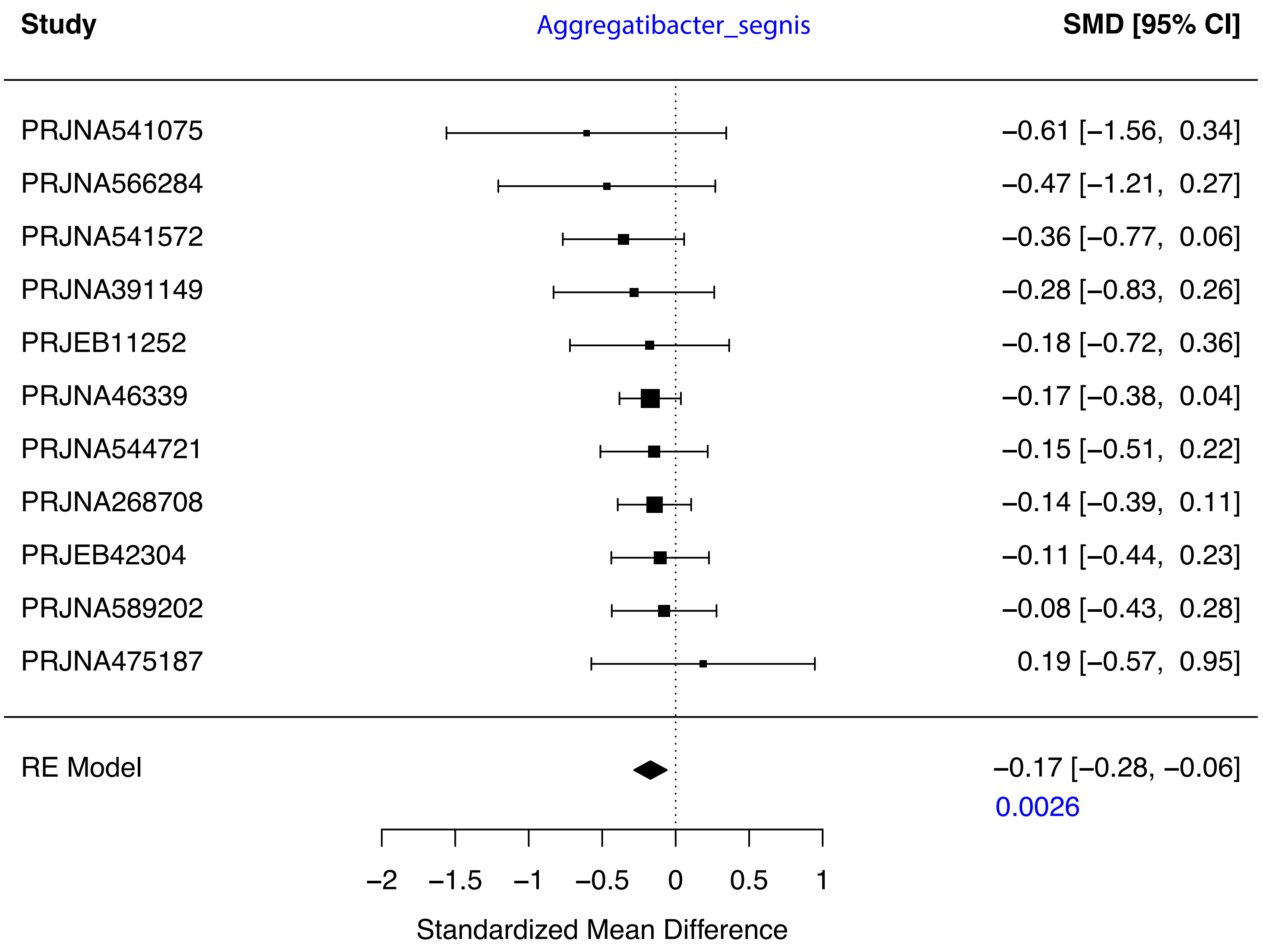
Study
Aggregatibacter_segnis
SMD [95% CI]
PRJNA541075 −0.61 [−1.56, 0.34]
PRJNA566284 −0.47 [−1.21, 0.27]
PRJNA541572 −0.36 [−0.77, 0.06]
PRJNA391149 −0.28 [−0.83, 0.26]
PRJEB11252 −0.18 [−0.72, 0.36]
PRJNA46339 −0.17 [−0.38, 0.04]
PRJNA544721 −0.15 [−0.51, 0.22]
PRJNA268708 −0.14 [−0.39, 0.11]
PRJEB42304 −0.11 [−0.44, 0.23]
PRJNA589202 −0.08 [−0.43, 0.28]
PRJNA475187 0.19 [−0.57, 0.95]
RE Model −0.17 [−0.28, −0.06]
0.0026
−2
−1.5
−1
−0.5
0
0.5
1
Standardized Mean Difference

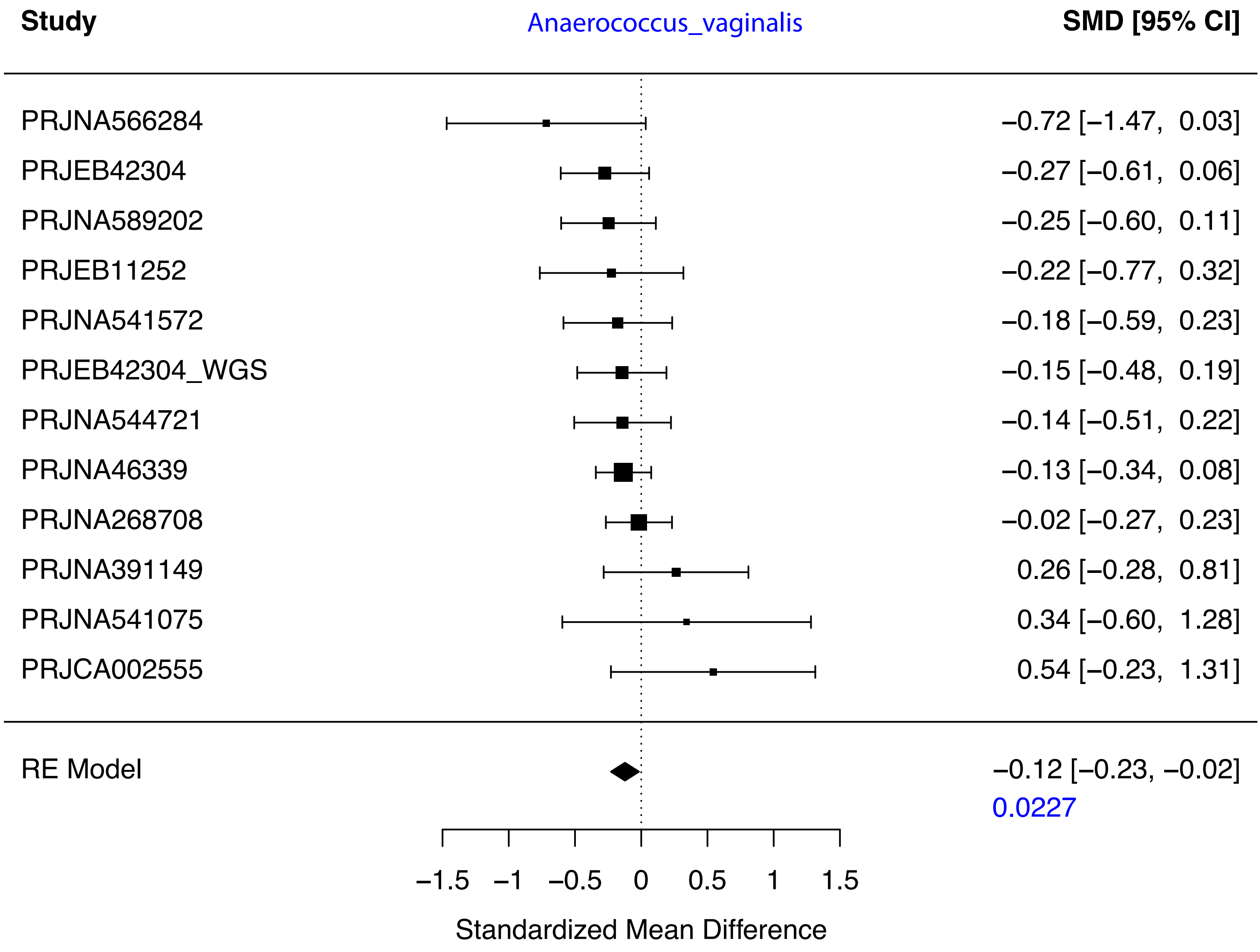
Study
Anaerococcus_vaginalis
SMD [95% CI]
PRJNA566284 −0.72 [−1.47, 0.03]
PRJEB42304 −0.27 [−0.61, 0.06]
PRJNA589202 −0.25 [−0.60, 0.11]
PRJEB11252 −0.22 [−0.77, 0.32]
PRJNA541572 −0.18 [−0.59, 0.23]
PRJEB42304_WGS −0.15 [−0.48, 0.19]
PRJNA544721 −0.14 [−0.51, 0.22]
PRJNA46339 −0.13 [−0.34, 0.08]
PRJNA268708 −0.02 [−0.27, 0.23]
PRJNA391149 0.26 [−0.28, 0.81]
PRJNA541075 0.34 [−0.60, 1.28]
PRJCA002555 0.54 [−0.23, 1.31]
RE Model −0.12 [−0.23, −0.02]
0.0227
−1.5
−1
−0.5
0
0.5
1
1.5
Standardized Mean Difference

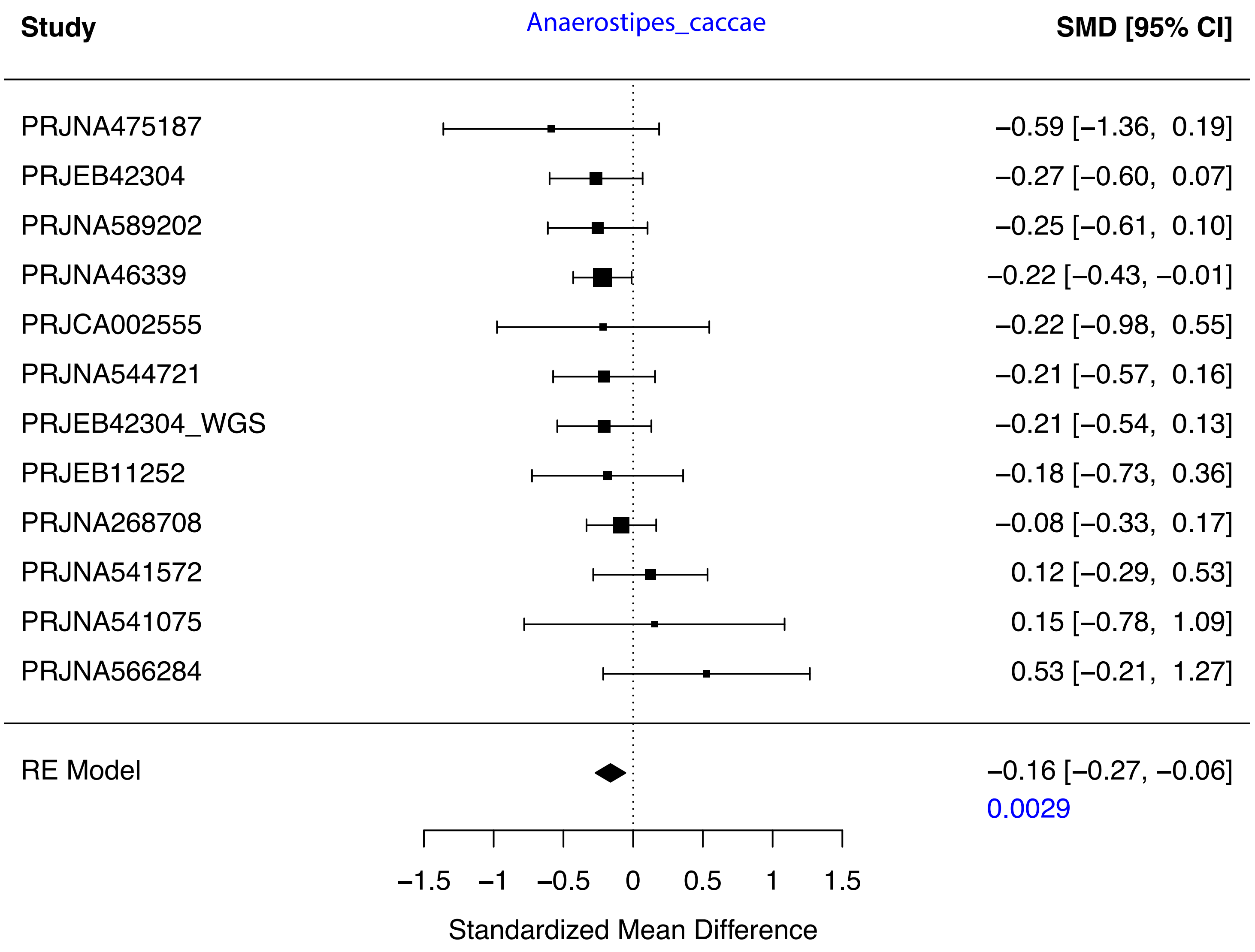
Study
Anaerostipes_caccae
SMD [95% CI]
PRJNA475187 −0.59 [−1.36, 0.19]
PRJEB42304 −0.27 [−0.60, 0.07]
PRJNA589202 −0.25 [−0.61, 0.10]
PRJNA46339 −0.22 [−0.43, −0.01]
PRJCA002555 −0.22 [−0.98, 0.55]
PRJNA544721 −0.21 [−0.57, 0.16]
PRJEB42304_WGS −0.21 [−0.54, 0.13]
PRJEB11252 −0.18 [−0.73, 0.36]
PRJNA268708 −0.08 [−0.33, 0.17]
PRJNA541572 0.12 [−0.29, 0.53]
PRJNA541075 0.15 [−0.78, 1.09]
PRJNA566284 0.53 [−0.21, 1.27]
RE Model −0.16 [−0.27, −0.06]
0.0029
−1.5
−1
−0.5
0
0.5
1
1.5
Standardized Mean Difference

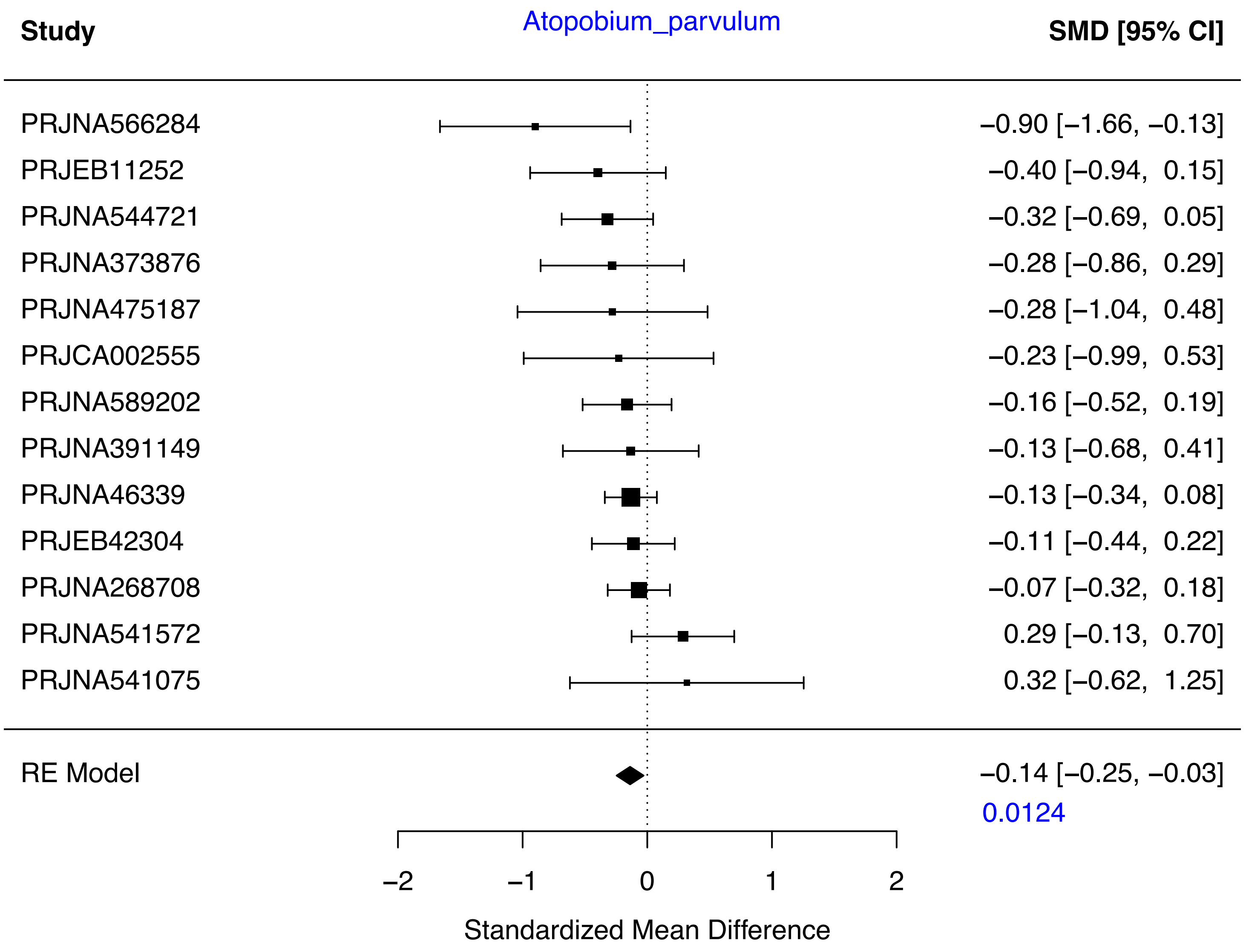
Study
Atopobium_parvulum
SMD [95% CI]
PRJNA566284 −0.90 [−1.66, −0.13]
PRJEB11252 −0.40 [−0.94, 0.15]
PRJNA544721 −0.32 [−0.69, 0.05]
PRJNA373876 −0.28 [−0.86, 0.29]
PRJNA475187 −0.28 [−1.04, 0.48]
PRJCA002555 −0.23 [−0.99, 0.53]
PRJNA589202 −0.16 [−0.52, 0.19]
PRJNA391149 −0.13 [−0.68, 0.41]
PRJNA46339 −0.13 [−0.34, 0.08]
PRJEB42304 −0.11 [−0.44, 0.22]
PRJNA268708 −0.07 [−0.32, 0.18]
PRJNA541572 0.29 [−0.13, 0.70]
PRJNA541075 0.32 [−0.62, 1.25]
RE Model −0.14 [−0.25, −0.03]
0.0124
−2
−1
0
1
2
Standardized Mean Difference

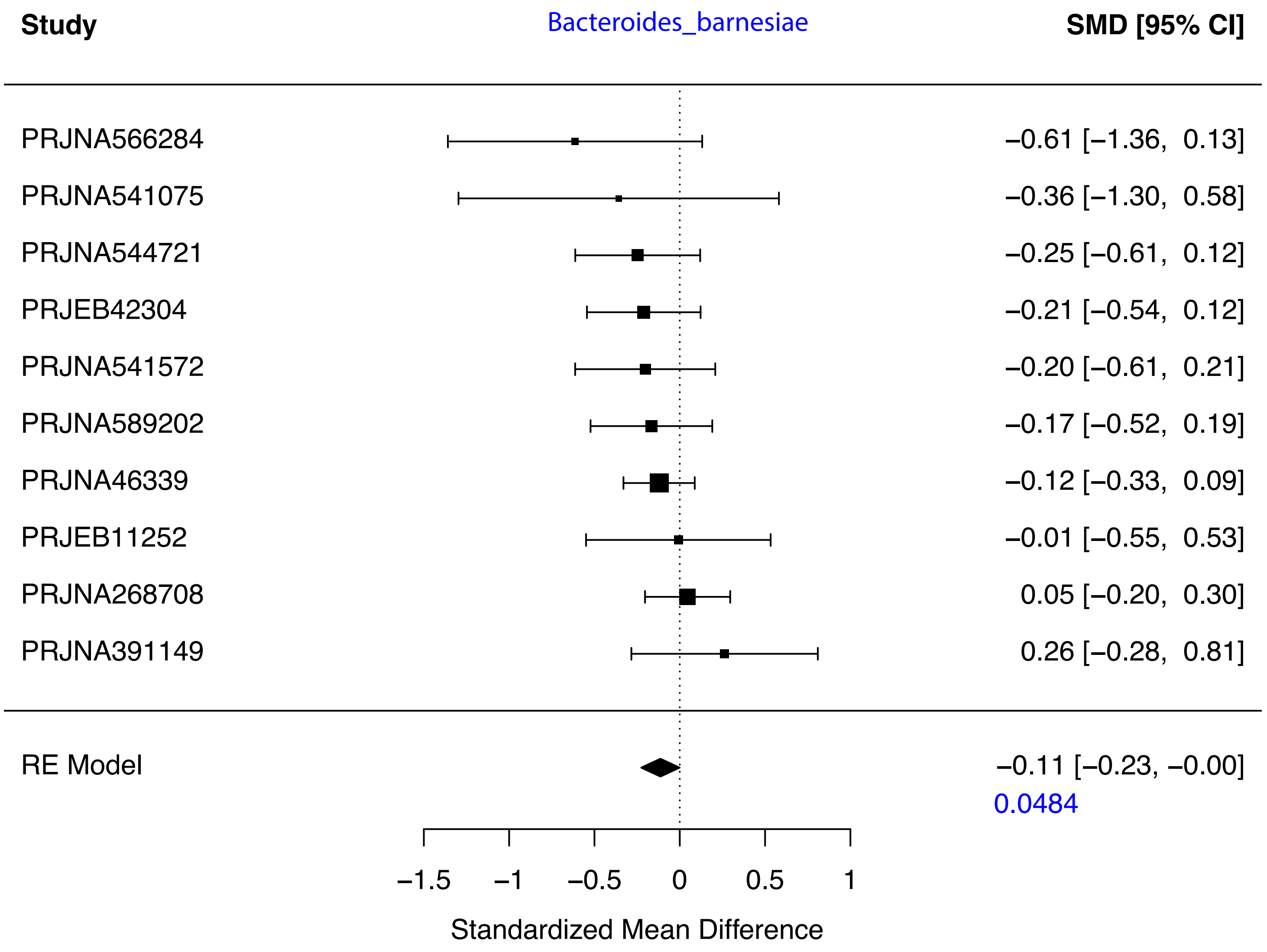
Study
Bacteroides_barnesiae
SMD [95% CI]
PRJNA566284 −0.61 [−1.36, 0.13]
PRJNA541075 −0.36 [−1.30, 0.58]
PRJNA544721 −0.25 [−0.61, 0.12]
PRJEB42304 −0.21 [−0.54, 0.12]
PRJNA541572 −0.20 [−0.61, 0.21]
PRJNA589202 −0.17 [−0.52, 0.19]
PRJNA46339 −0.12 [−0.33, 0.09]
PRJEB11252 −0.01 [−0.55, 0.53]
PRJNA268708 0.05 [−0.20, 0.30]
PRJNA391149 0.26 [−0.28, 0.81]
RE Model −0.11 [−0.23, −0.00]
0.0484
−1.5
−1
−0.5
0
0.5
1
Standardized Mean Difference

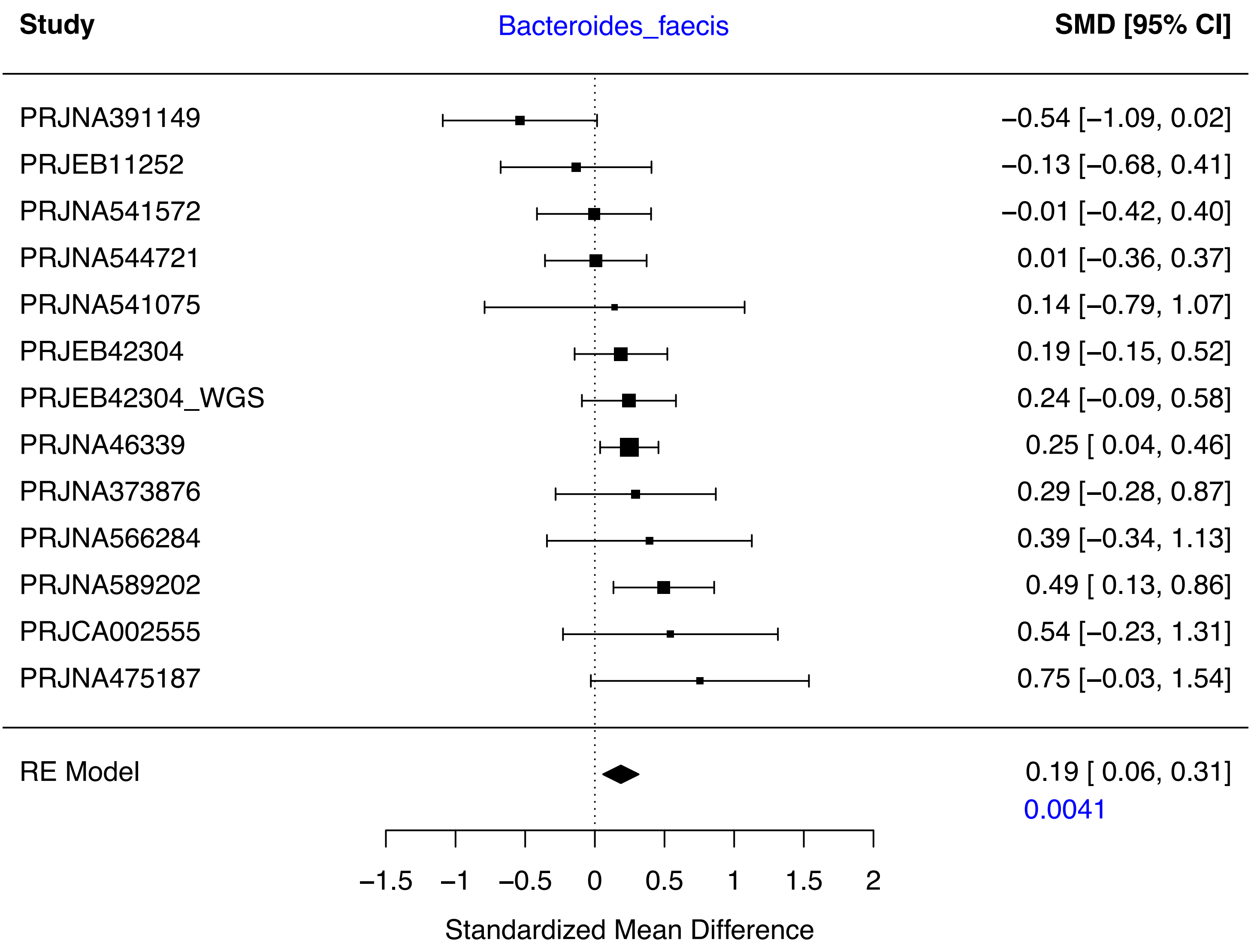
Study
Bacteroides_faecis
SMD [95% CI]
PRJNA391149 −0.54 [−1.09, 0.02]
PRJEB11252 −0.13 [−0.68, 0.41]
PRJNA541572 −0.01 [−0.42, 0.40]
PRJNA544721 0.01 [−0.36, 0.37]
PRJNA541075 0.14 [−0.79, 1.07]
PRJEB42304 0.19 [−0.15, 0.52]
PRJEB42304_WGS 0.24 [−0.09, 0.58]
PRJNA46339 0.25 [0.04, 0.46]
PRJNA373876 0.29 [−0.28, 0.87]
PRJNA566284 0.39 [−0.34, 1.13]
PRJNA589202 0.49 [0.13, 0.86]
PRJCA002555 0.54 [−0.23, 1.31]
PRJNA475187 0.75 [−0.03, 1.54]
RE Model 0.19 [0.06, 0.31]
0.0041
−1.5 −1 −0.5 0 0.5 1 1.5 2
Standardized Mean Difference

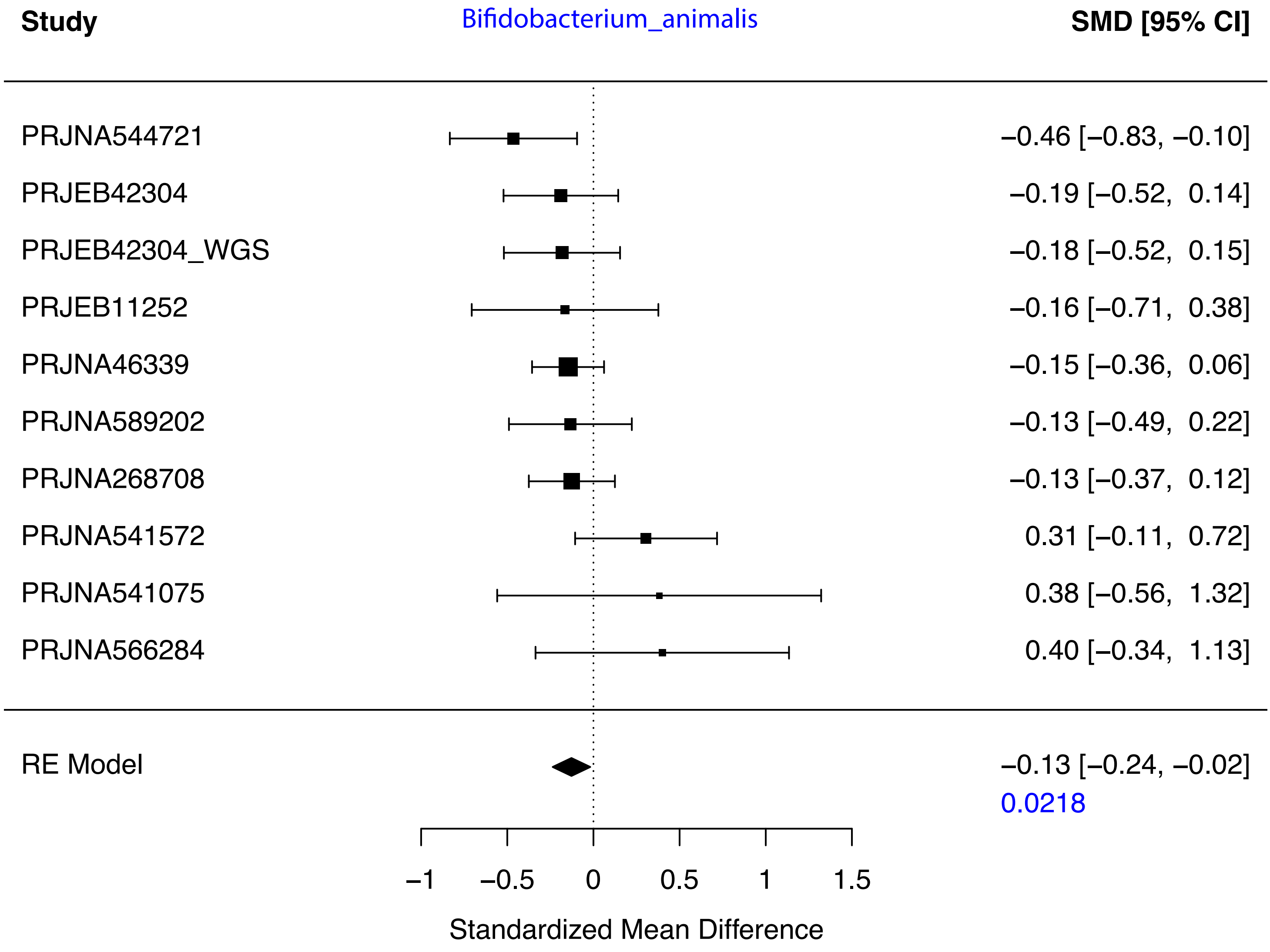
Study
Bifidobacterium_animalis
SMD [95% CI]
PRJNA544721
−0.46 [−0.83, −0.10]
PRJEB42304
−0.19 [−0.52, 0.14]
PRJEB42304_WGS
−0.18 [−0.52, 0.15]
PRJEB11252
−0.16 [−0.71, 0.38]
PRJNA46339
−0.15 [−0.36, 0.06]
PRJNA589202
−0.13 [−0.49, 0.22]
PRJNA268708
−0.13 [−0.37, 0.12]
PRJNA541572
0.31 [−0.11, 0.72]
PRJNA541075
0.38 [−0.56, 1.32]
PRJNA566284
0.40 [−0.34, 1.13]
RE Model
−0.13 [−0.24, −0.02]
0.0218
−1
−0.5
0
0.5
1
1.5
Standardized Mean Difference

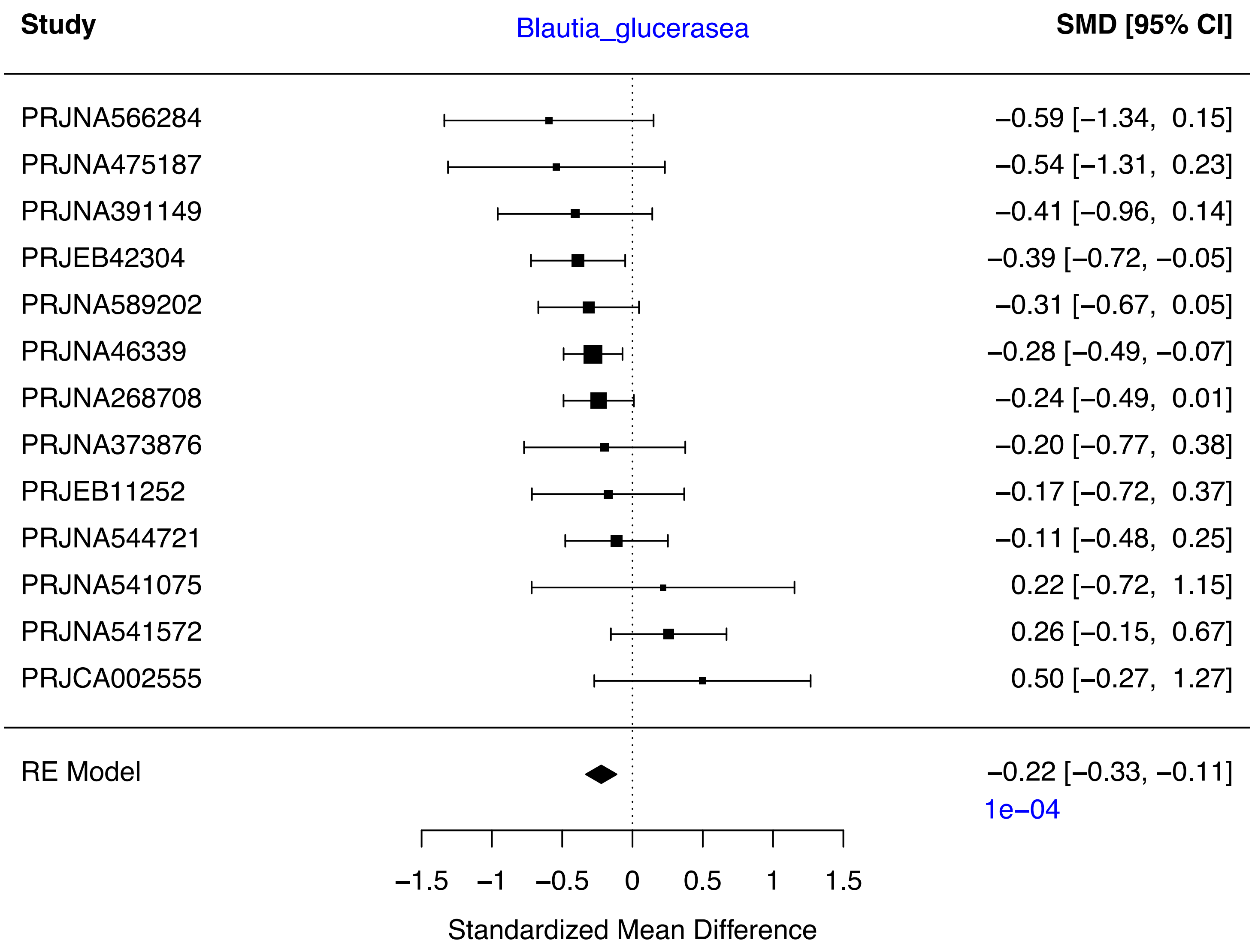
Study
Blautia_glucerasea
SMD [95% CI]
PRJNA566284 −0.59 [−1.34, 0.15]
PRJNA475187 −0.54 [−1.31, 0.23]
PRJNA391149 −0.41 [−0.96, 0.14]
PRJEB42304 −0.39 [−0.72, −0.05]
PRJNA589202 −0.31 [−0.67, 0.05]
PRJNA46339 −0.28 [−0.49, −0.07]
PRJNA268708 −0.24 [−0.49, 0.01]
PRJNA373876 −0.20 [−0.77, 0.38]
PRJEB11252 −0.17 [−0.72, 0.37]
PRJNA544721 −0.11 [−0.48, 0.25]
PRJNA541075 0.22 [−0.72, 1.15]
PRJNA541572 0.26 [−0.15, 0.67]
PRJCA002555 0.50 [−0.27, 1.27]
RE Model −0.22 [−0.33, −0.11]
1e−04
−1.5 −1 −0.5 0 0.5 1 1.5
Standardized Mean Difference

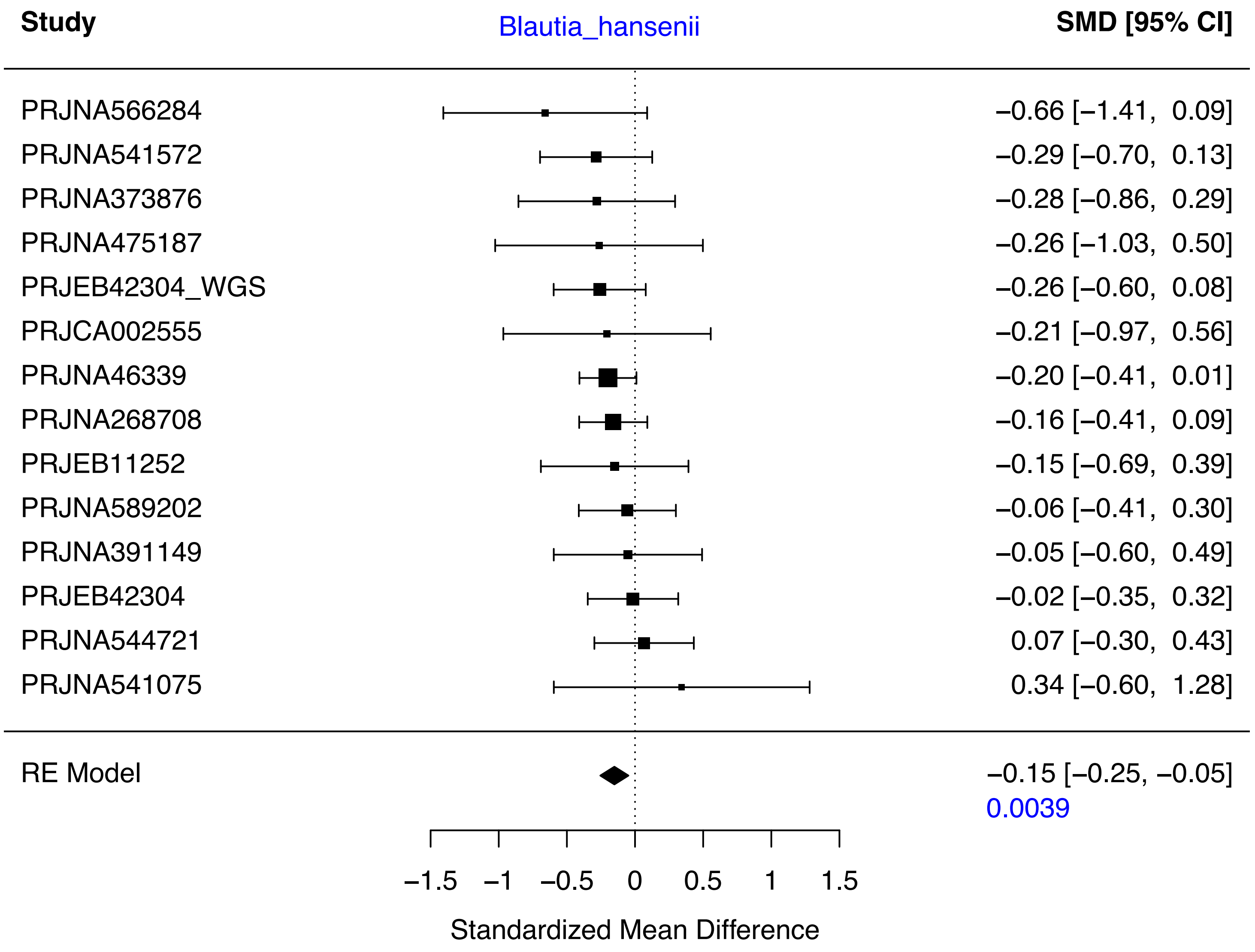
Study
Blautia_hansenii
SMD [95% CI]
PRJNA566284 −0.66 [−1.41, 0.09]
PRJNA541572 −0.29 [−0.70, 0.13]
PRJNA373876 −0.28 [−0.86, 0.29]
PRJNA475187 −0.26 [−1.03, 0.50]
PRJEB42304_WGS −0.26 [−0.60, 0.08]
PRJCA002555 −0.21 [−0.97, 0.56]
PRJNA46339 −0.20 [−0.41, 0.01]
PRJNA268708 −0.16 [−0.41, 0.09]
PRJEB11252 −0.15 [−0.69, 0.39]
PRJNA589202 −0.06 [−0.41, 0.30]
PRJNA391149 −0.05 [−0.60, 0.49]
PRJEB42304 −0.02 [−0.35, 0.32]
PRJNA544721 0.07 [−0.30, 0.43]
PRJNA541075 0.34 [−0.60, 1.28]
RE Model −0.15 [−0.25, −0.05]
0.0039
−1.5 −1 −0.5 0 0.5 1 1.5
Standardized Mean Difference

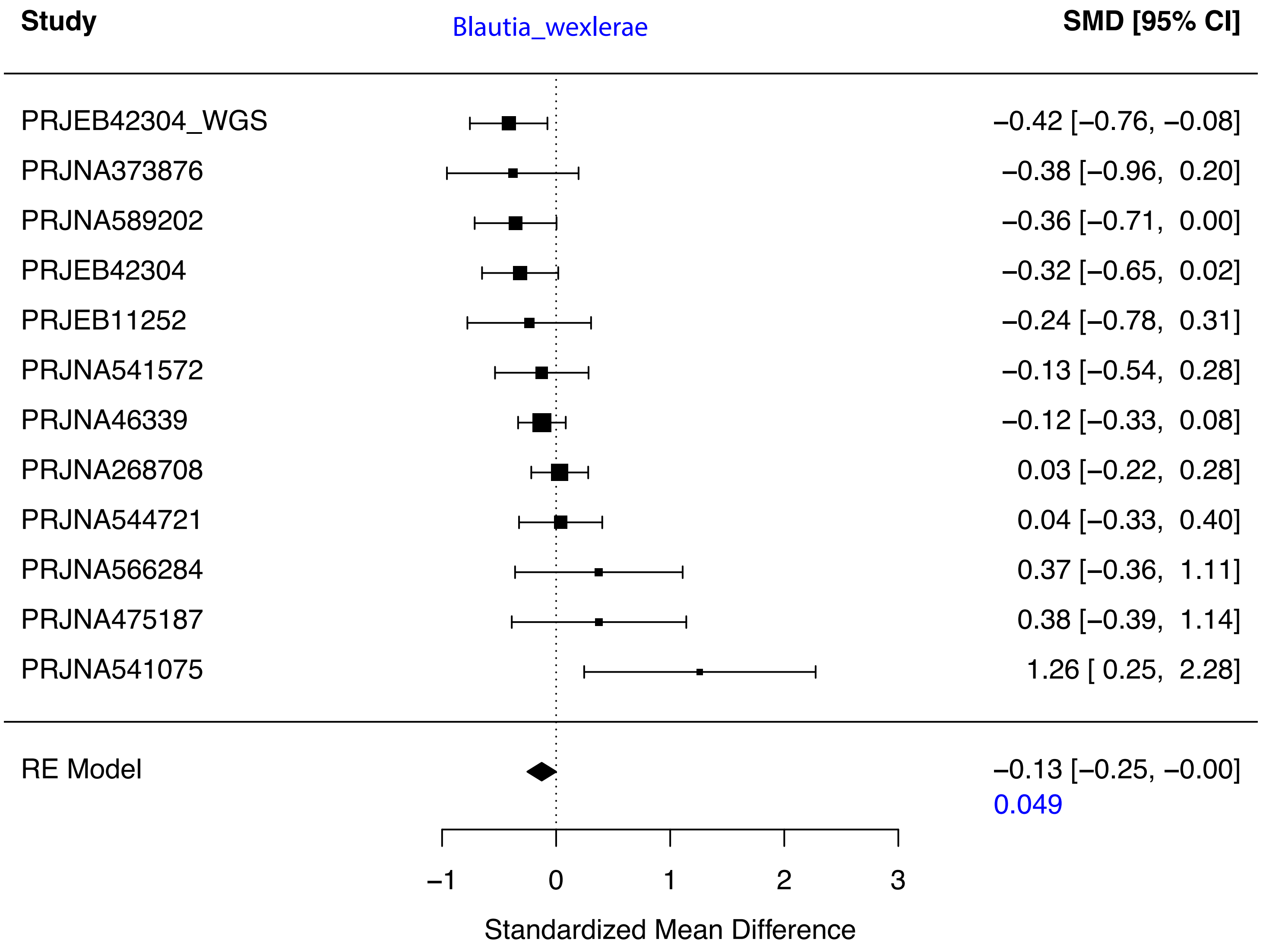
Study
Blautia_wexlerae
SMD [95% CI]
PRJEB42304_WGS
PRJNA373876
PRJNA589202
PRJEB42304
PRJEB11252
PRJNA541572
PRJNA46339
PRJNA268708
PRJNA544721
PRJNA566284
PRJNA475187
PRJNA541075
−0.42 [−0.76, −0.08]
−0.38 [−0.96, 0.20]
−0.36 [−0.71, 0.00]
−0.32 [−0.65, 0.02]
−0.24 [−0.78, 0.31]
−0.13 [−0.54, 0.28]
−0.12 [−0.33, 0.08]
0.03 [−0.22, 0.28]
0.04 [−0.33, 0.40]
0.37 [−0.36, 1.11]
0.38 [−0.39, 1.14]
1.26 [0.25, 2.28]
RE Model
−0.13 [−0.25, −0.00]
0.049
−1
0
1
2
3
Standardized Mean Difference

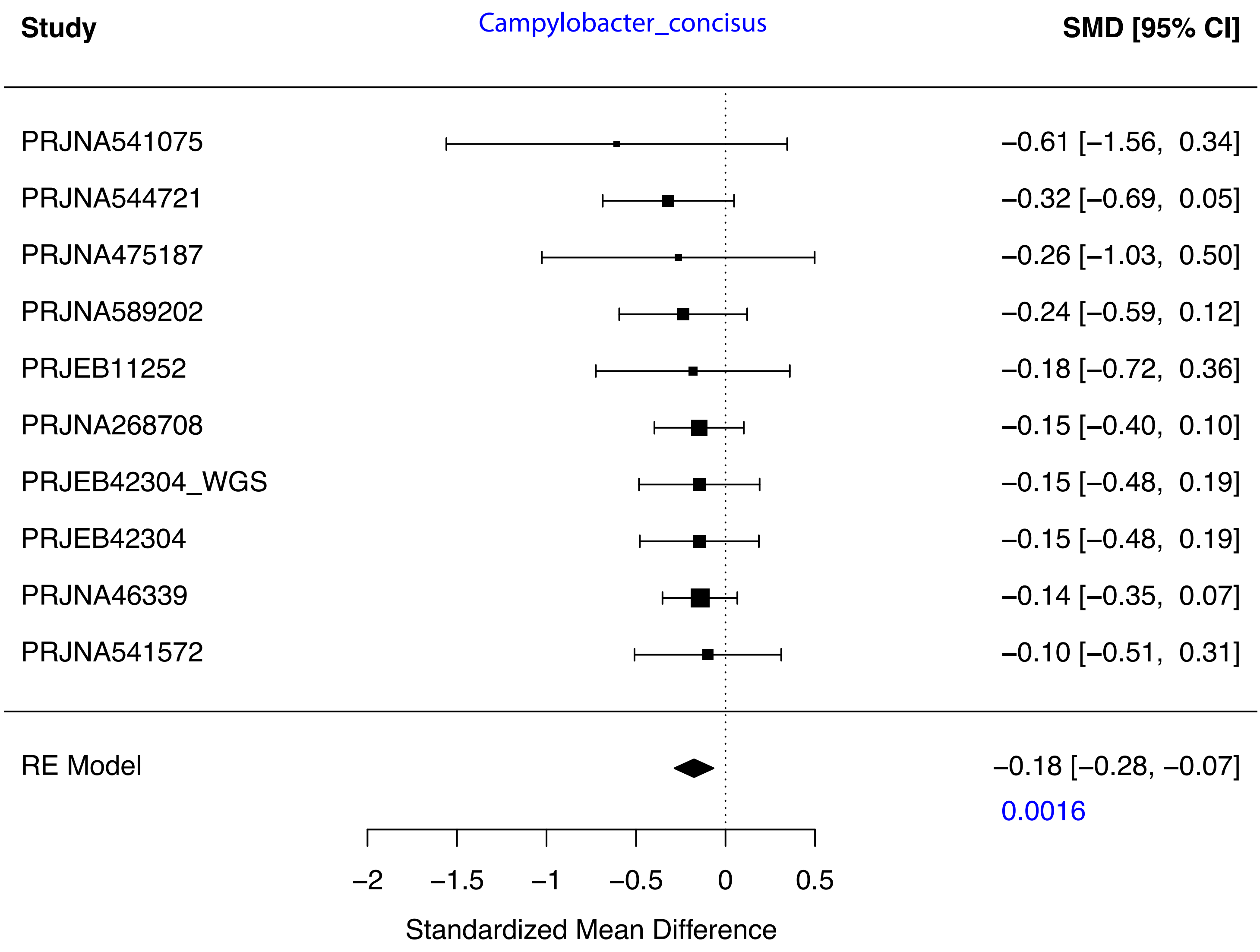
Study
Campylobacter_concisus
SMD [95% CI]
PRJNA541075
−0.61 [−1.56, 0.34]
PRJNA544721
−0.32 [−0.69, 0.05]
PRJNA475187
−0.26 [−1.03, 0.50]
PRJNA589202
−0.24 [−0.59, 0.12]
PRJEB11252
−0.18 [−0.72, 0.36]
PRJNA268708
−0.15 [−0.40, 0.10]
PRJEB42304_WGS
−0.15 [−0.48, 0.19]
PRJEB42304
−0.15 [−0.48, 0.19]
PRJNA46339
−0.14 [−0.35, 0.07]
PRJNA541572
−0.10 [−0.51, 0.31]
RE Model
−0.18 [−0.28, −0.07]
0.0016
−2
−1.5
−1
−0.5
0
0.5
Standardized Mean Difference

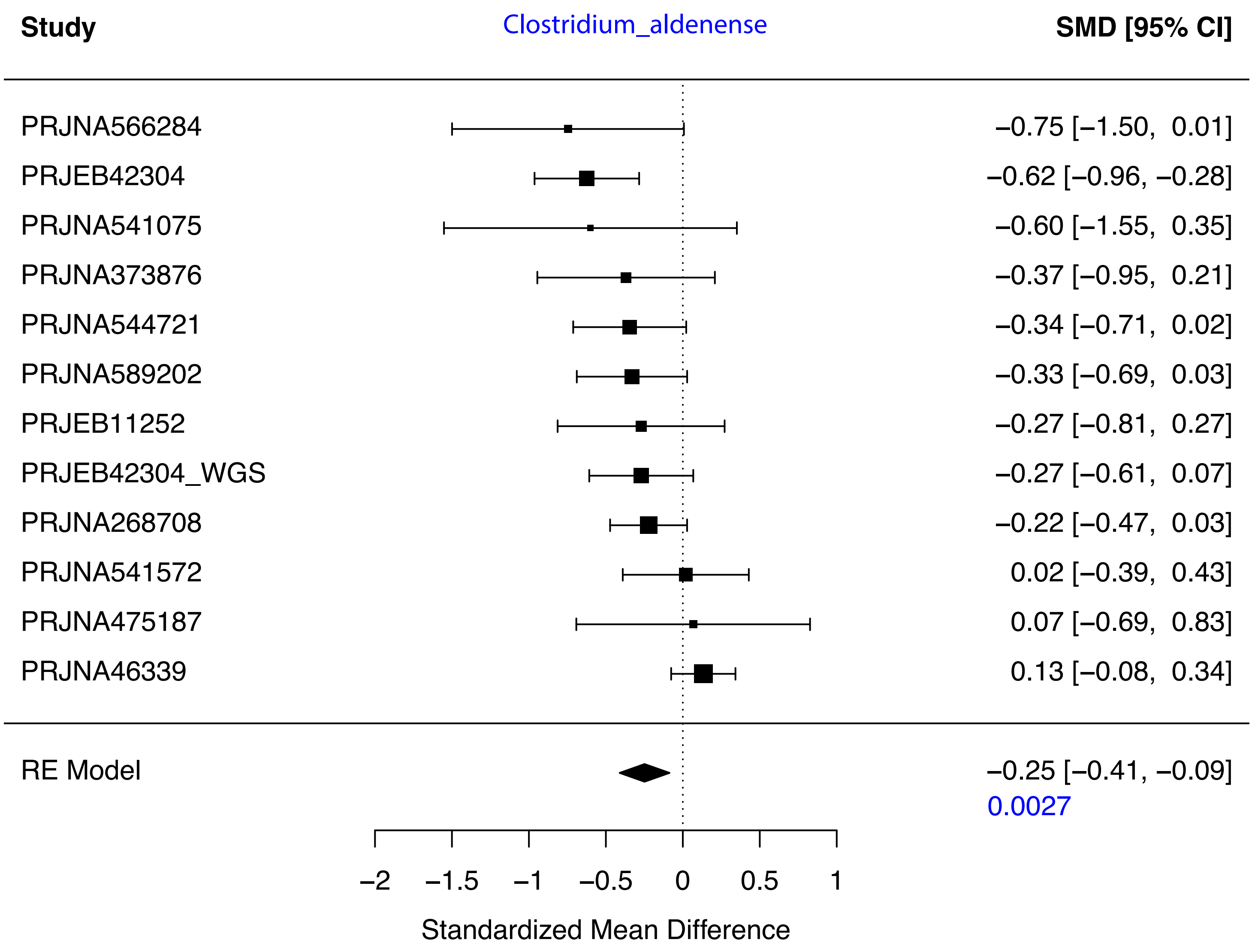
Study
Clostridium_aldenense
SMD [95% CI]
PRJNA566284 −0.75 [−1.50, 0.01]
PRJEB42304 −0.62 [−0.96, −0.28]
PRJNA541075 −0.60 [−1.55, 0.35]
PRJNA373876 −0.37 [−0.95, 0.21]
PRJNA544721 −0.34 [−0.71, 0.02]
PRJNA589202 −0.33 [−0.69, 0.03]
PRJEB11252 −0.27 [−0.81, 0.27]
PRJEB42304_WGS −0.27 [−0.61, 0.07]
PRJNA268708 −0.22 [−0.47, 0.03]
PRJNA541572 0.02 [−0.39, 0.43]
PRJNA475187 0.07 [−0.69, 0.83]
PRJNA46339 0.13 [−0.08, 0.34]
RE Model −0.25 [−0.41, −0.09]
0.0027
−2
−1.5
−1
−0.5
0
0.5
1
Standardized Mean Difference

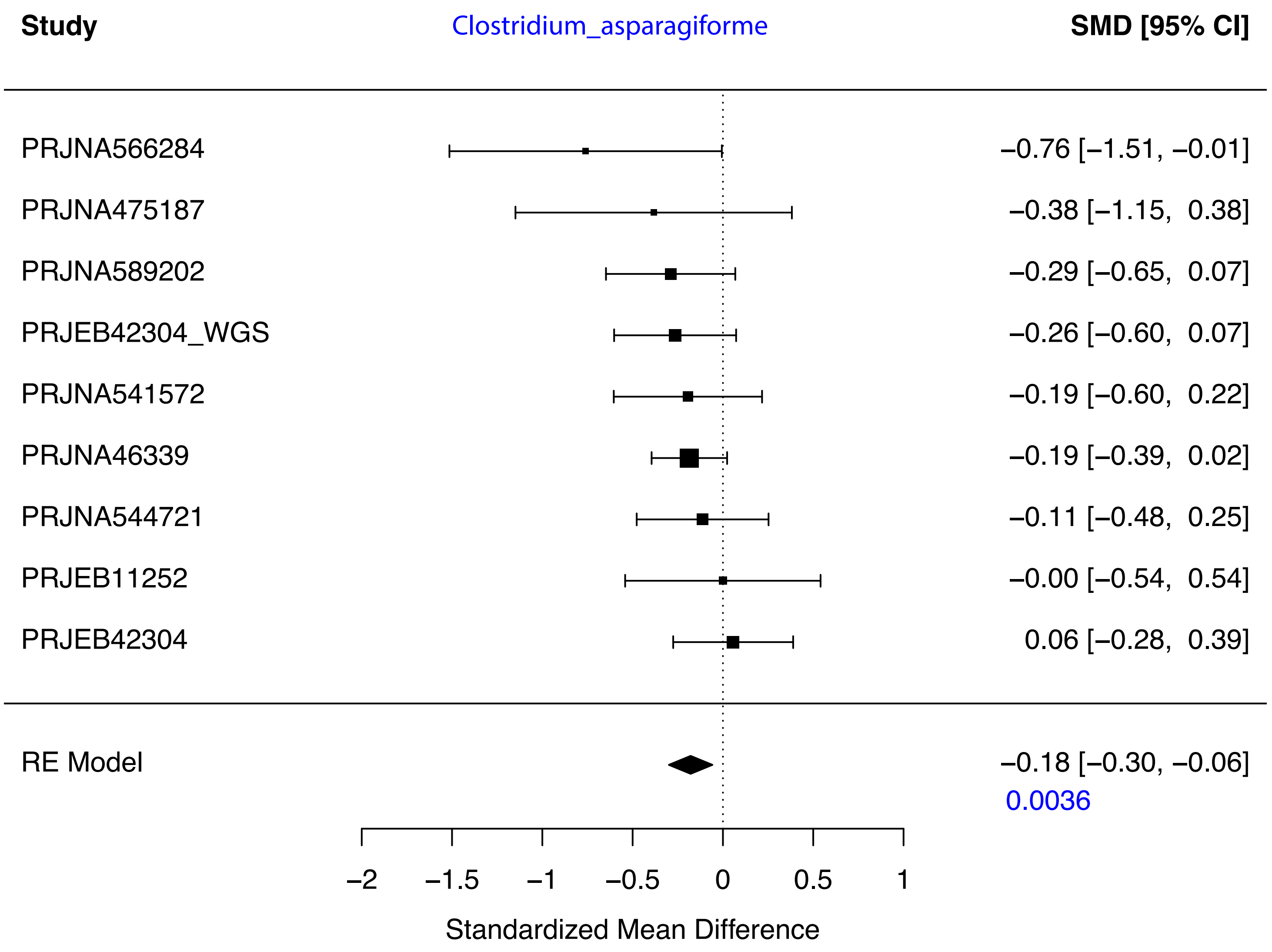

Study
Clostridium_asparagiforme
SMD [95% CI]
PRJNA566284
−0.76 [−1.51, −0.01]
PRJNA475187
−0.38 [−1.15, 0.38]
PRJNA589202
−0.29 [−0.65, 0.07]
PRJEB42304_WGS
−0.26 [−0.60, 0.07]
PRJNA541572
−0.19 [−0.60, 0.22]
PRJNA46339
−0.19 [−0.39, 0.02]
PRJNA544721
−0.11 [−0.48, 0.25]
PRJEB11252
−0.00 [−0.54, 0.54]
PRJEB42304
0.06 [−0.28, 0.39]
RE Model
−0.18 [−0.30, −0.06]
0.0036
−2
−1.5
−1
−0.5
0
0.5
1
Standardized Mean Difference

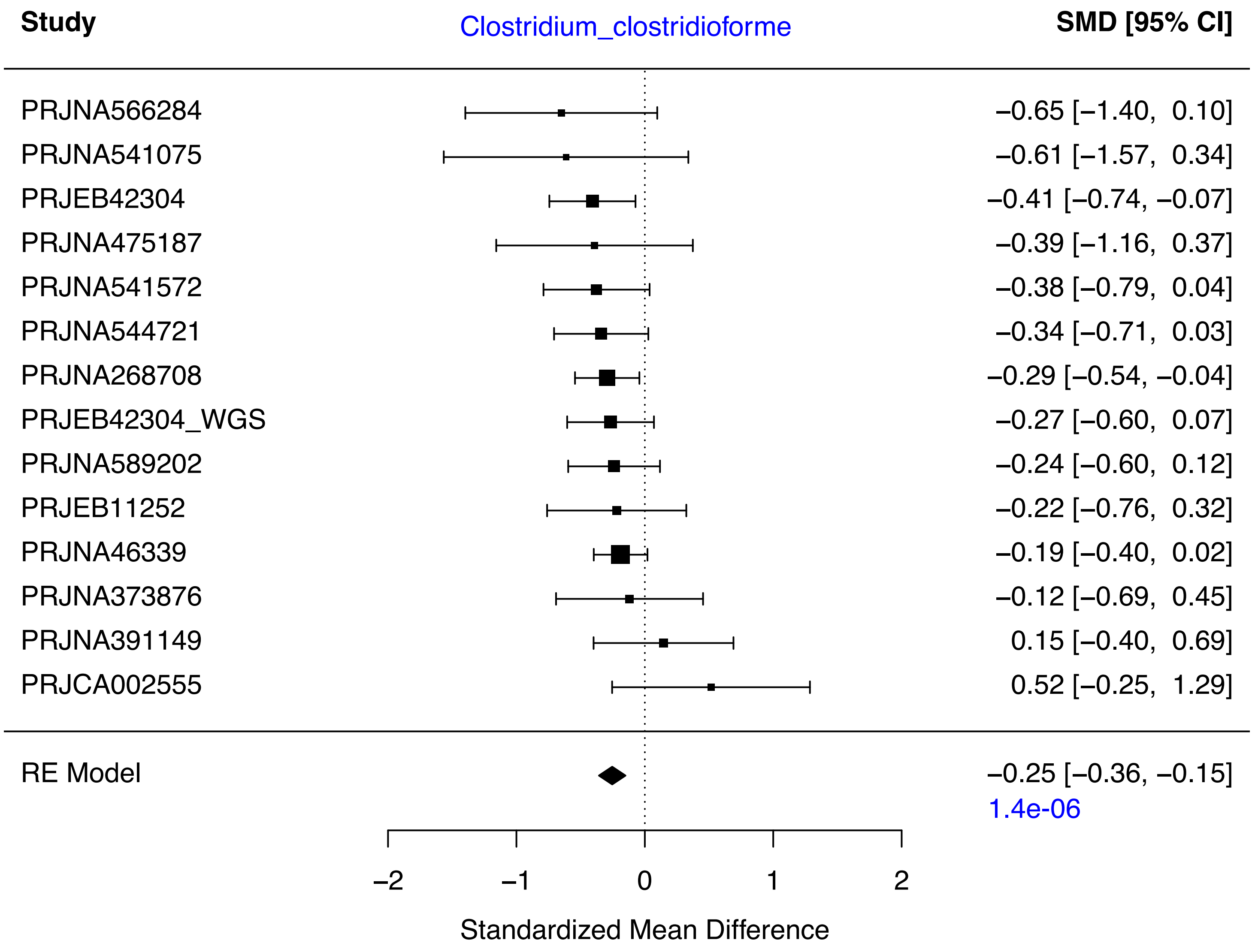
Study
Clostridium_clostridioforme
SMD [95% CI]
PRJNA566284 −0.65 [−1.40, 0.10]
PRJNA541075 −0.61 [−1.57, 0.34]
PRJEB42304 −0.41 [−0.74, −0.07]
PRJNA475187 −0.39 [−1.16, 0.37]
PRJNA541572 −0.38 [−0.79, 0.04]
PRJNA544721 −0.34 [−0.71, 0.03]
PRJNA268708 −0.29 [−0.54, −0.04]
PRJEB42304_WGS −0.27 [−0.60, 0.07]
PRJNA589202 −0.24 [−0.60, 0.12]
PRJEB11252 −0.22 [−0.76, 0.32]
PRJNA46339 −0.19 [−0.40, 0.02]
PRJNA373876 −0.12 [−0.69, 0.45]
PRJNA391149 0.15 [−0.40, 0.69]
PRJCA002555 0.52 [−0.25, 1.29]
RE Model −0.25 [−0.36, −0.15]
1.4e-06
−2
−1
0
1
2
Standardized Mean Difference

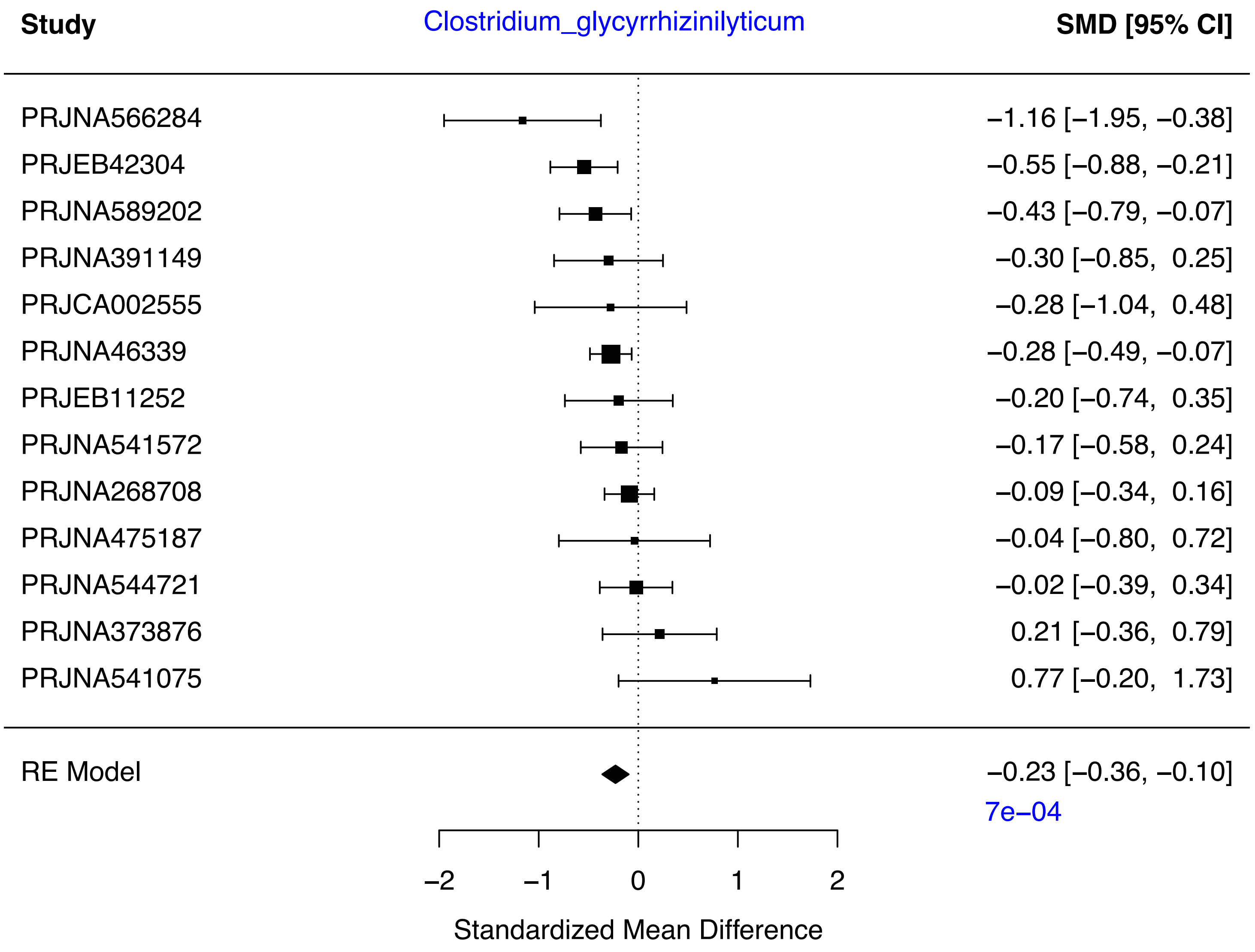
Study
Clostridium_glycyrrhizinilyticum
SMD [95% CI]
PRJNA566284 −1.16 [−1.95, −0.38]
PRJEB42304 −0.55 [−0.88, −0.21]
PRJNA589202 −0.43 [−0.79, −0.07]
PRJNA391149 −0.30 [−0.85, 0.25]
PRJCA002555 −0.28 [−1.04, 0.48]
PRJNA46339 −0.28 [−0.49, −0.07]
PRJEB11252 −0.20 [−0.74, 0.35]
PRJNA541572 −0.17 [−0.58, 0.24]
PRJNA268708 −0.09 [−0.34, 0.16]
PRJNA475187 −0.04 [−0.80, 0.72]
PRJNA544721 −0.02 [−0.39, 0.34]
PRJNA373876 0.21 [−0.36, 0.79]
PRJNA541075 0.77 [−0.20, 1.73]
RE Model −0.23 [−0.36, −0.10]
7e−04
−2
−1
0
1
2
Standardized Mean Difference

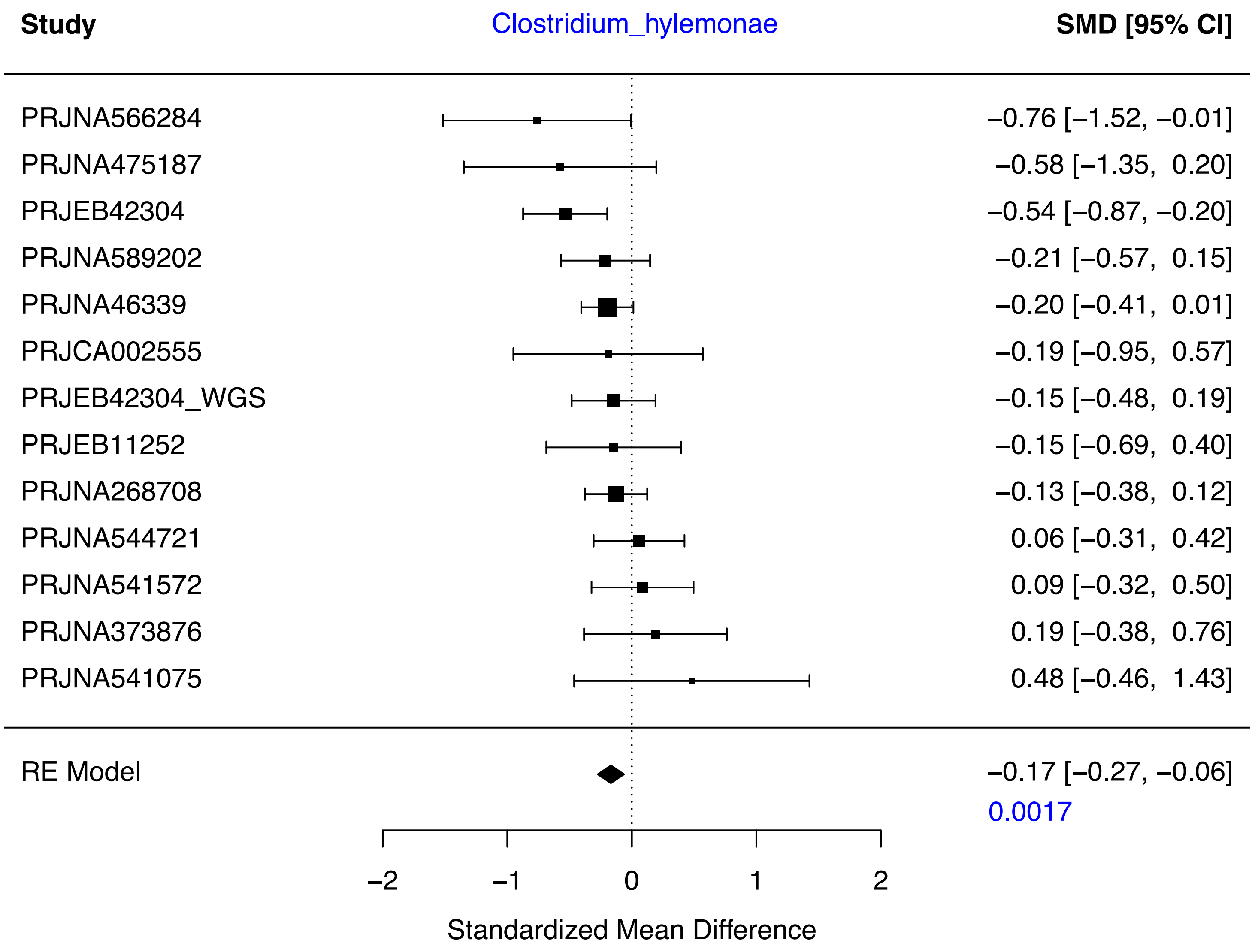
Study
Clostridium_hylemonae
SMD [95% CI]
PRJNA566284 −0.76 [−1.52, −0.01]
PRJNA475187 −0.58 [−1.35, 0.20]
PRJEB42304 −0.54 [−0.87, −0.20]
PRJNA589202 −0.21 [−0.57, 0.15]
PRJNA46339 −0.20 [−0.41, 0.01]
PRJCA002555 −0.19 [−0.95, 0.57]
PRJEB42304_WGS −0.15 [−0.48, 0.19]
PRJEB11252 −0.15 [−0.69, 0.40]
PRJNA268708 −0.13 [−0.38, 0.12]
PRJNA544721 0.06 [−0.31, 0.42]
PRJNA541572 0.09 [−0.32, 0.50]
PRJNA373876 0.19 [−0.38, 0.76]
PRJNA541075 0.48 [−0.46, 1.43]
RE Model −0.17 [−0.27, −0.06]
0.0017
−2
−1
0
1
2
Standardized Mean Difference

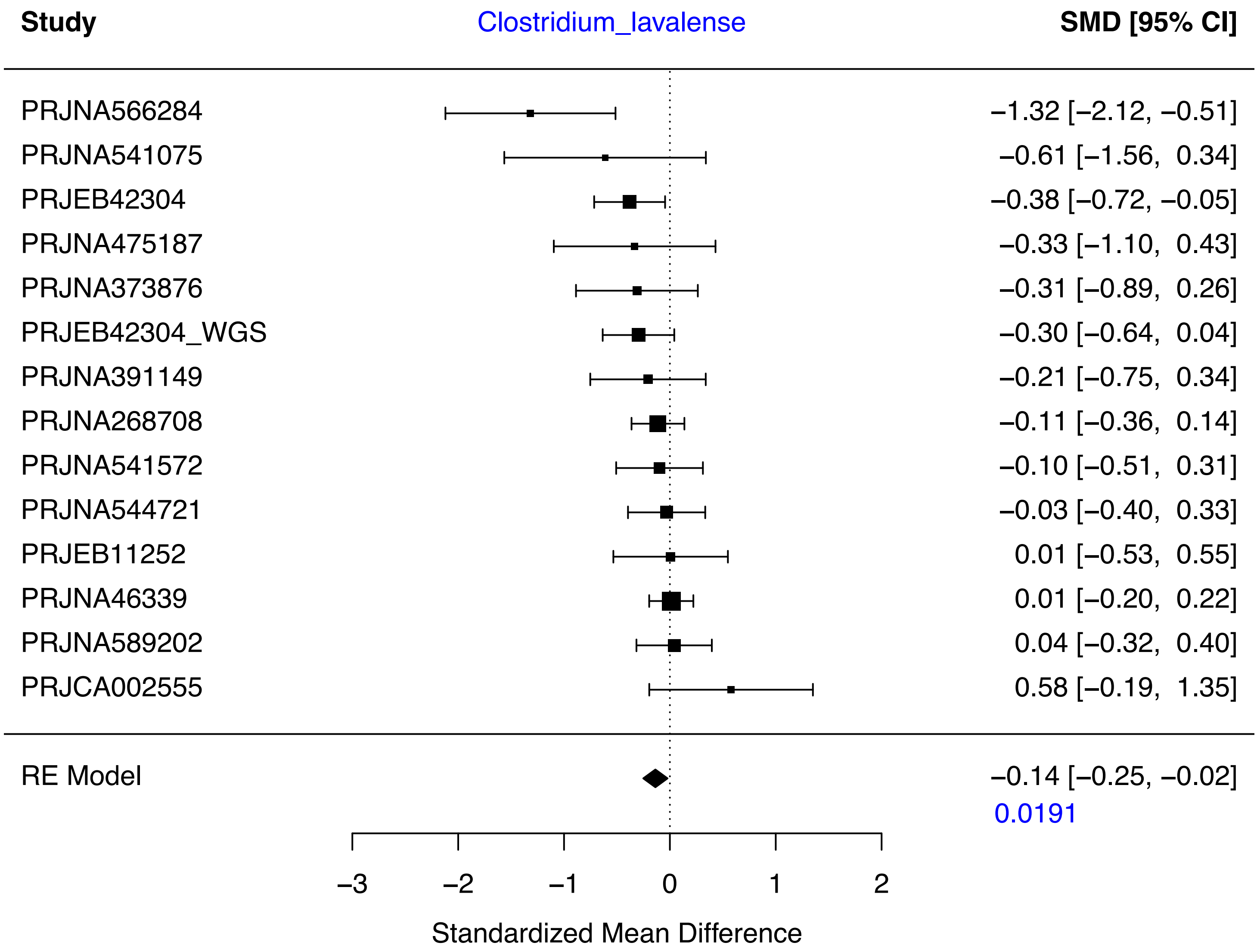
Study
Clostridium_lavalense
SMD [95% CI]
PRJNA566284 −1.32 [−2.12, −0.51]
PRJNA541075 −0.61 [−1.56, 0.34]
PRJEB42304 −0.38 [−0.72, −0.05]
PRJNA475187 −0.33 [−1.10, 0.43]
PRJNA373876 −0.31 [−0.89, 0.26]
PRJEB42304_WGS −0.30 [−0.64, 0.04]
PRJNA391149 −0.21 [−0.75, 0.34]
PRJNA268708 −0.11 [−0.36, 0.14]
PRJNA541572 −0.10 [−0.51, 0.31]
PRJNA544721 −0.03 [−0.40, 0.33]
PRJEB11252 0.01 [−0.53, 0.55]
PRJNA46339 0.01 [−0.20, 0.22]
PRJNA589202 0.04 [−0.32, 0.40]
PRJCA002555 0.58 [−0.19, 1.35]
RE Model −0.14 [−0.25, −0.02]
0.0191
−3
−2
−1
0
1
2
Standardized Mean Difference

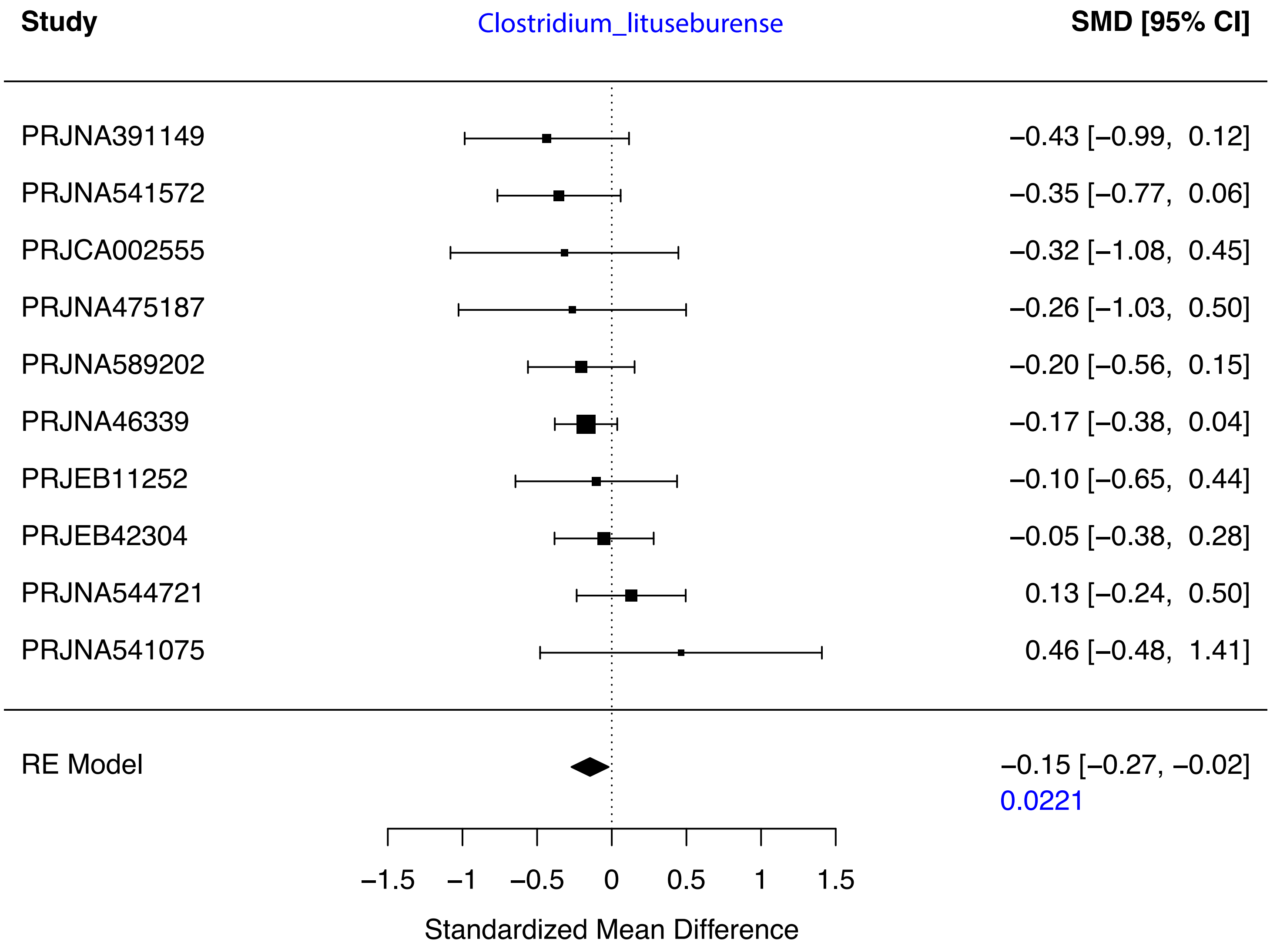
Study
Clostridium_lituseburense
SMD [95% CI]
PRJNA391149 −0.43 [−0.99, 0.12]
PRJNA541572 −0.35 [−0.77, 0.06]
PRJCA002555 −0.32 [−1.08, 0.45]
PRJNA475187 −0.26 [−1.03, 0.50]
PRJNA589202 −0.20 [−0.56, 0.15]
PRJNA46339 −0.17 [−0.38, 0.04]
PRJEB11252 −0.10 [−0.65, 0.44]
PRJEB42304 −0.05 [−0.38, 0.28]
PRJNA544721 0.13 [−0.24, 0.50]
PRJNA541075 0.46 [−0.48, 1.41]
RE Model −0.15 [−0.27, −0.02]
0.0221
−1.5 −1 −0.5 0 0.5 1 1.5
Standardized Mean Difference

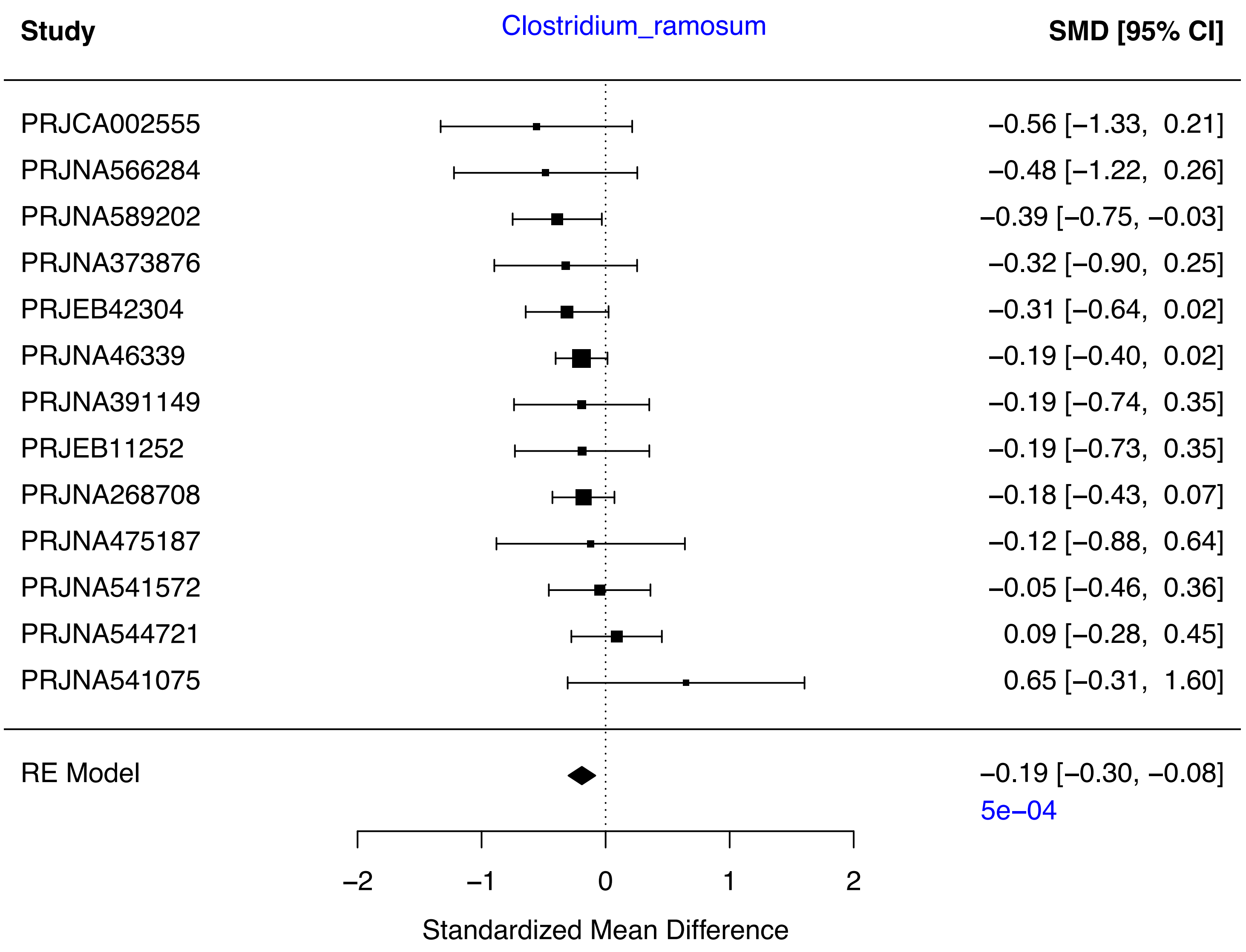
Study
Clostridium_ramosum
SMD [95% CI]
PRJCA002555 −0.56 [−1.33, 0.21]
PRJNA566284 −0.48 [−1.22, 0.26]
PRJNA589202 −0.39 [−0.75, −0.03]
PRJNA373876 −0.32 [−0.90, 0.25]
PRJEB42304 −0.31 [−0.64, 0.02]
PRJNA46339 −0.19 [−0.40, 0.02]
PRJNA391149 −0.19 [−0.74, 0.35]
PRJEB11252 −0.19 [−0.73, 0.35]
PRJNA268708 −0.18 [−0.43, 0.07]
PRJNA475187 −0.12 [−0.88, 0.64]
PRJNA541572 −0.05 [−0.46, 0.36]
PRJNA544721 0.09 [−0.28, 0.45]
PRJNA541075 0.65 [−0.31, 1.60]
RE Model −0.19 [−0.30, −0.08]
5e−04
−2
−1
0
1
2
Standardized Mean Difference

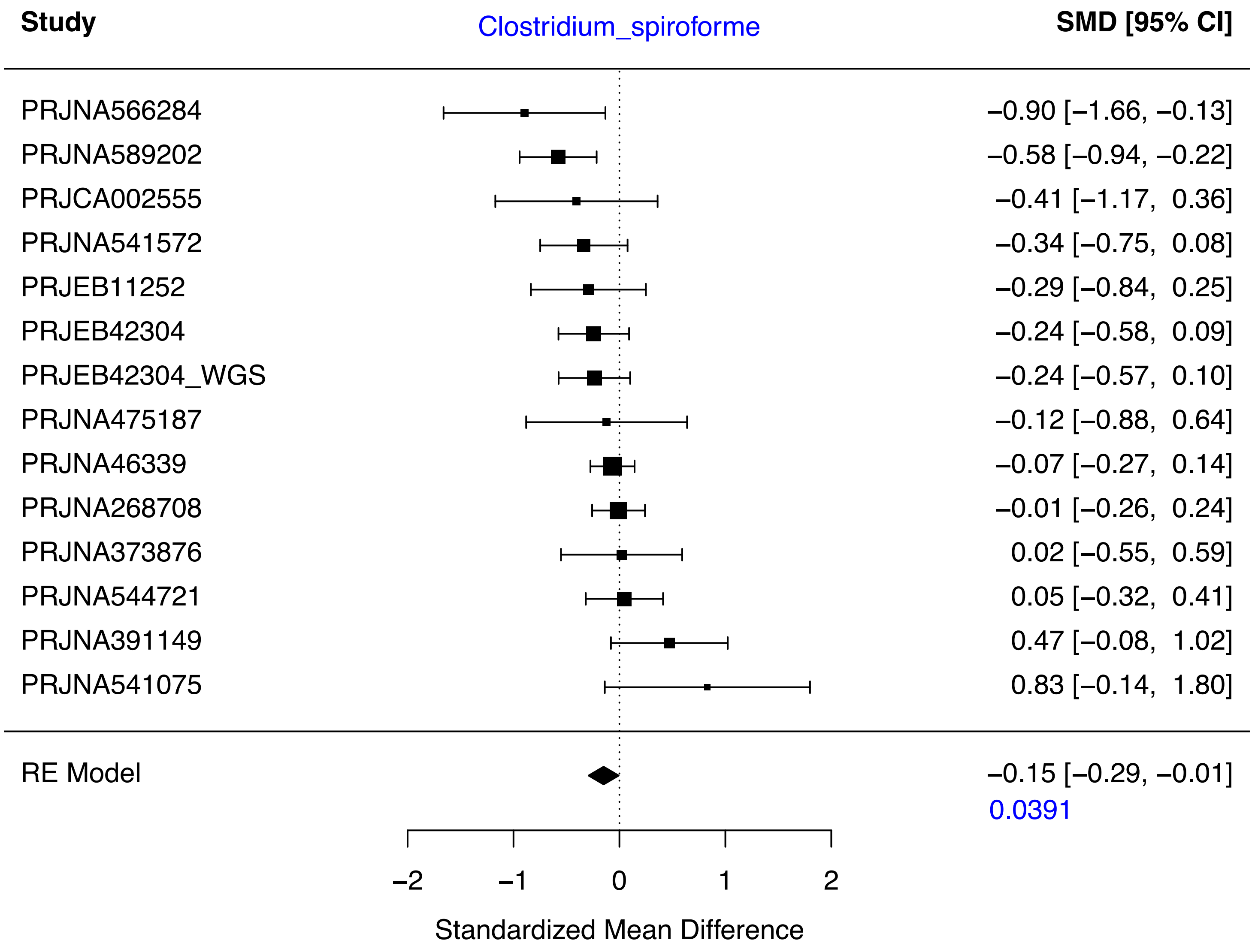
Study
Clostridium_spiroforme
SMD [95% CI]
PRJNA566284 −0.90 [−1.66, −0.13]
PRJNA589202 −0.58 [−0.94, −0.22]
PRJCA002555 −0.41 [−1.17, 0.36]
PRJNA541572 −0.34 [−0.75, 0.08]
PRJEB11252 −0.29 [−0.84, 0.25]
PRJEB42304 −0.24 [−0.58, 0.09]
PRJEB42304_WGS −0.24 [−0.57, 0.10]
PRJNA475187 −0.12 [−0.88, 0.64]
PRJNA46339 −0.07 [−0.27, 0.14]
PRJNA268708 −0.01 [−0.26, 0.24]
PRJNA373876 0.02 [−0.55, 0.59]
PRJNA544721 0.05 [−0.32, 0.41]
PRJNA391149 0.47 [−0.08, 1.02]
PRJNA541075 0.83 [−0.14, 1.80]
RE Model −0.15 [−0.29, −0.01]
0.0391
−2
−1
0
1
2
Standardized Mean Difference

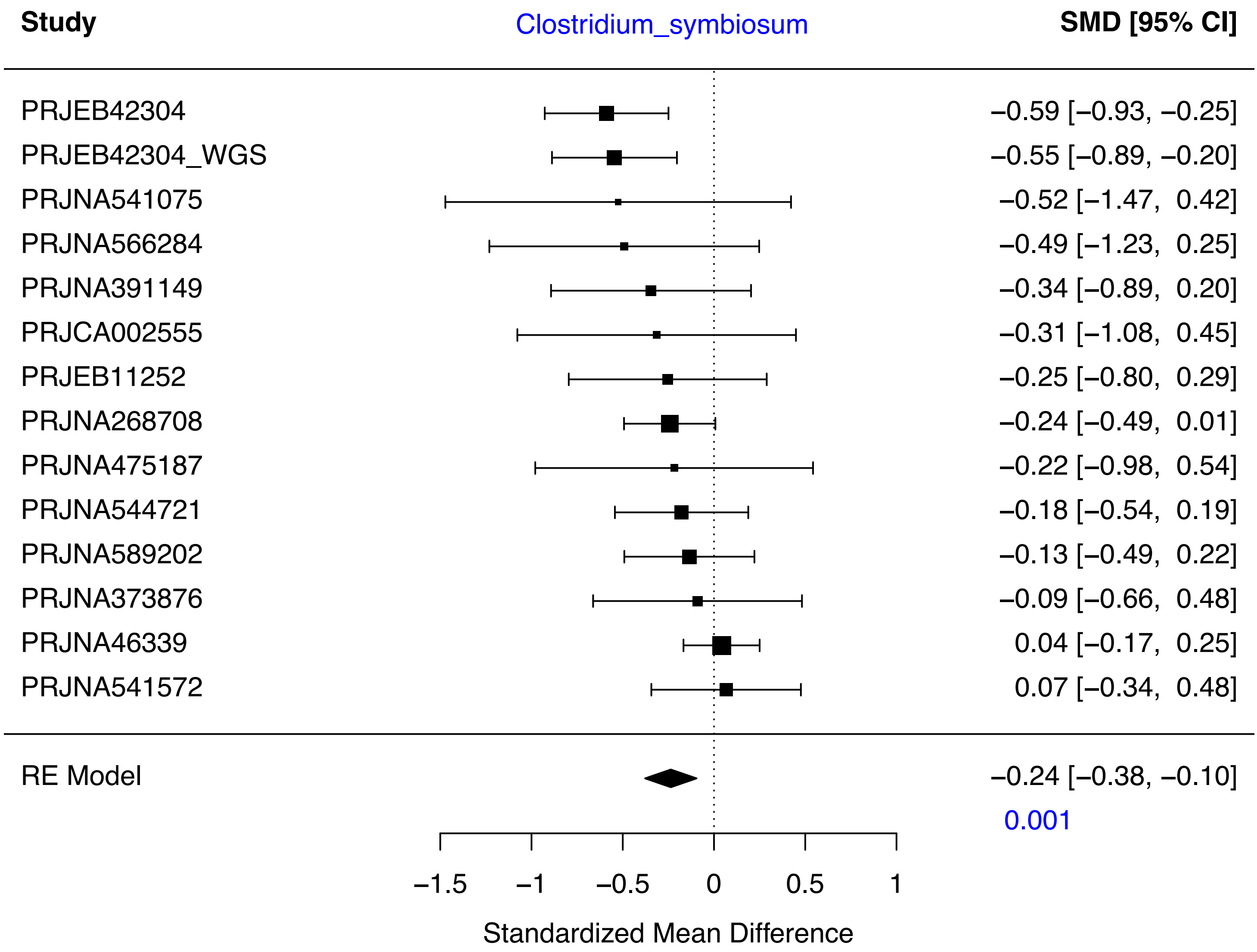
Study
Clostridium_symbiosum
SMD [95% CI]
PRJEB42304 −0.59 [−0.93, −0.25]
PRJEB42304_WGS −0.55 [−0.89, −0.20]
PRJNA541075 −0.52 [−1.47, 0.42]
PRJNA566284 −0.49 [−1.23, 0.25]
PRJNA391149 −0.34 [−0.89, 0.20]
PRJCA002555 −0.31 [−1.08, 0.45]
PRJEB11252 −0.25 [−0.80, 0.29]
PRJNA268708 −0.24 [−0.49, 0.01]
PRJNA475187 −0.22 [−0.98, 0.54]
PRJNA544721 −0.18 [−0.54, 0.19]
PRJNA589202 −0.13 [−0.49, 0.22]
PRJNA373876 −0.09 [−0.66, 0.48]
PRJNA46339 0.04 [−0.17, 0.25]
PRJNA541572 0.07 [−0.34, 0.48]
RE Model −0.24 [−0.38, −0.10]
0.001
−1.5
−1
−0.5
0
0.5
1
Standardized Mean Difference

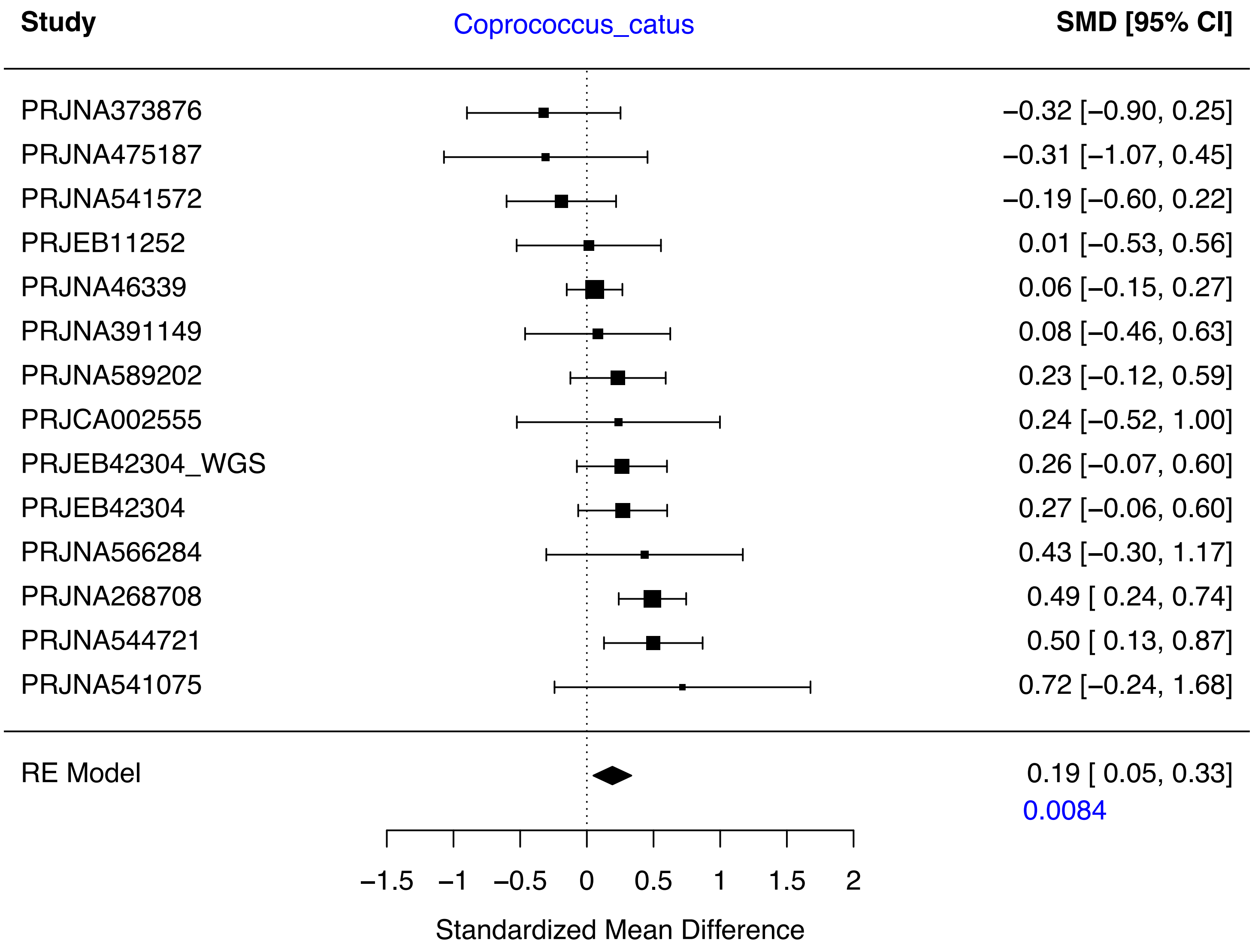
Study
Coprococcus_catus
SMD [95% CI]
PRJNA373876 −0.32 [−0.90, 0.25]
PRJNA475187 −0.31 [−1.07, 0.45]
PRJNA541572 −0.19 [−0.60, 0.22]
PRJEB11252 0.01 [−0.53, 0.56]
PRJNA46339 0.06 [−0.15, 0.27]
PRJNA391149 0.08 [−0.46, 0.63]
PRJNA589202 0.23 [−0.12, 0.59]
PRJCA002555 0.24 [−0.52, 1.00]
PRJEB42304_WGS 0.26 [−0.07, 0.60]
PRJEB42304 0.27 [−0.06, 0.60]
PRJNA566284 0.43 [−0.30, 1.17]
PRJNA268708 0.49 [0.24, 0.74]
PRJNA544721 0.50 [0.13, 0.87]
PRJNA541075 0.72 [−0.24, 1.68]
RE Model 0.19 [0.05, 0.33]
0.0084
−1.5 −1 −0.5 0 0.5 1 1.5 2
Standardized Mean Difference

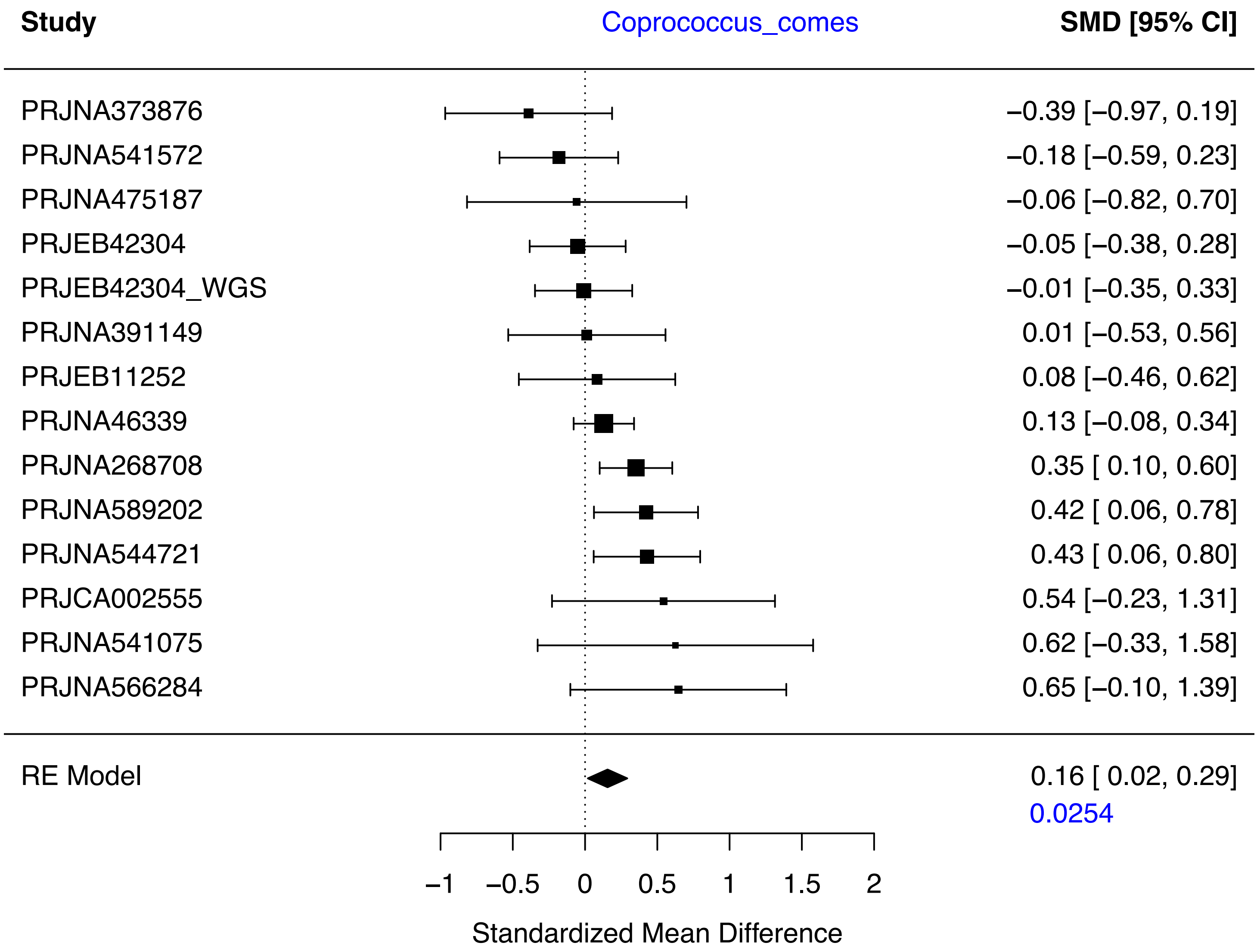
Study
Coprococcus_comes
SMD [95% CI]
PRJNA373876
PRJNA541572
PRJNA475187
PRJEB42304
PRJEB42304_WGS
PRJNA391149
PRJEB11252
PRJNA46339
PRJNA268708
PRJNA589202
PRJNA544721
PRJCA002555
PRJNA541075
PRJNA566284
RE Model
−0.39 [−0.97, 0.19]
−0.18 [−0.59, 0.23]
−0.06 [−0.82, 0.70]
−0.05 [−0.38, 0.28]
−0.01 [−0.35, 0.33]
0.01 [−0.53, 0.56]
0.08 [−0.46, 0.62]
0.13 [−0.08, 0.34]
0.35 [0.10, 0.60]
0.42 [0.06, 0.78]
0.43 [0.06, 0.80]
0.54 [−0.23, 1.31]
0.62 [−0.33, 1.58]
0.65 [−0.10, 1.39]
0.16 [0.02, 0.29]
0.0254
−1
−0.5
0
0.5
1
1.5
2
Standardized Mean Difference

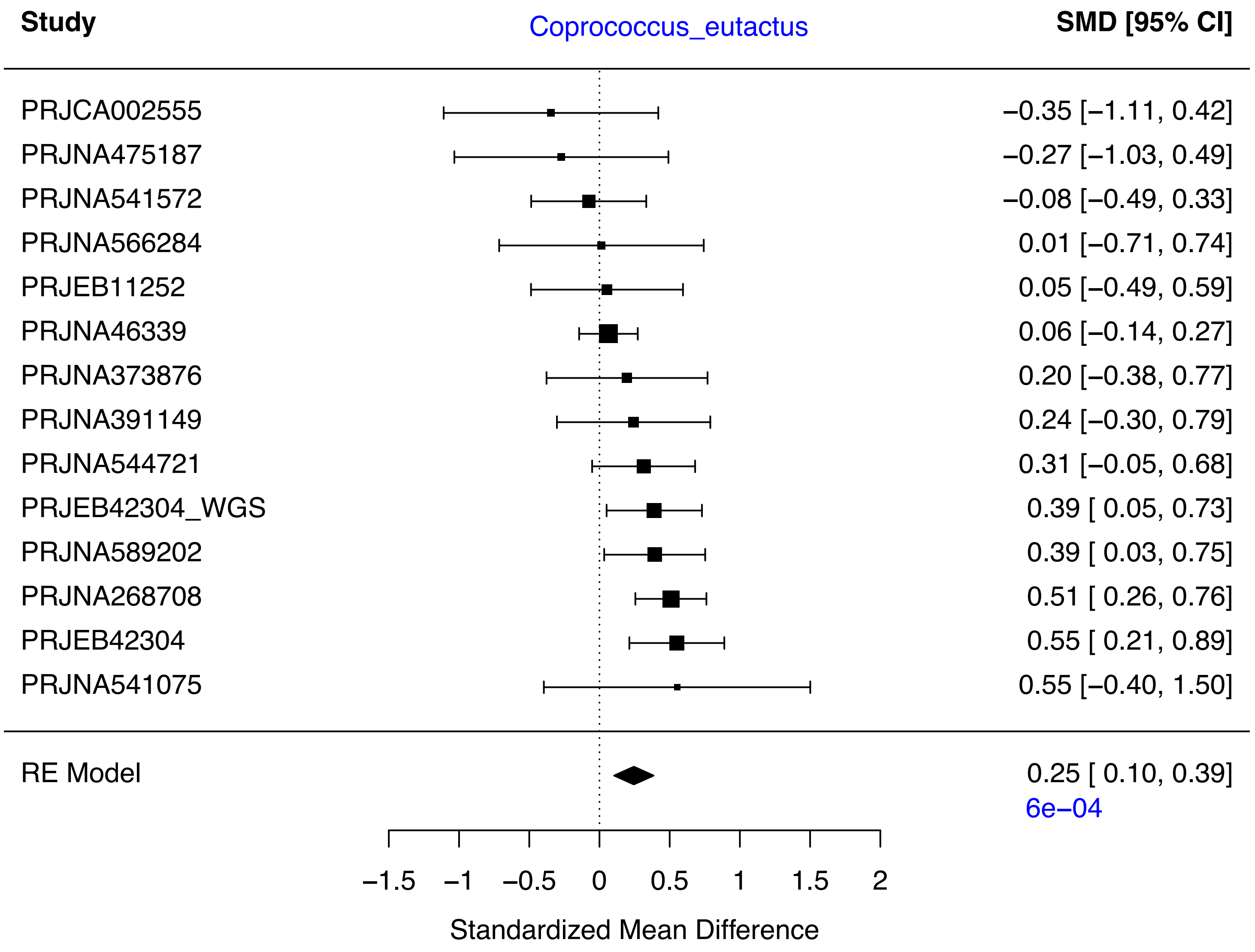
Study
Coprococcus_eutactus
SMD [95% CI]
PRJCA002555 −0.35 [−1.11, 0.42]
PRJNA475187 −0.27 [−1.03, 0.49]
PRJNA541572 −0.08 [−0.49, 0.33]
PRJNA566284 0.01 [−0.71, 0.74]
PRJEB11252 0.05 [−0.49, 0.59]
PRJNA46339 0.06 [−0.14, 0.27]
PRJNA373876 0.20 [−0.38, 0.77]
PRJNA391149 0.24 [−0.30, 0.79]
PRJNA544721 0.31 [−0.05, 0.68]
PRJEB42304_WGS 0.39 [0.05, 0.73]
PRJNA589202 0.39 [0.03, 0.75]
PRJNA268708 0.51 [0.26, 0.76]
PRJEB42304 0.55 [0.21, 0.89]
PRJNA541075 0.55 [−0.40, 1.50]
RE Model 0.25 [0.10, 0.39]
6e−04
−1.5 −1 −0.5 0 0.5 1 1.5 2
Standardized Mean Difference

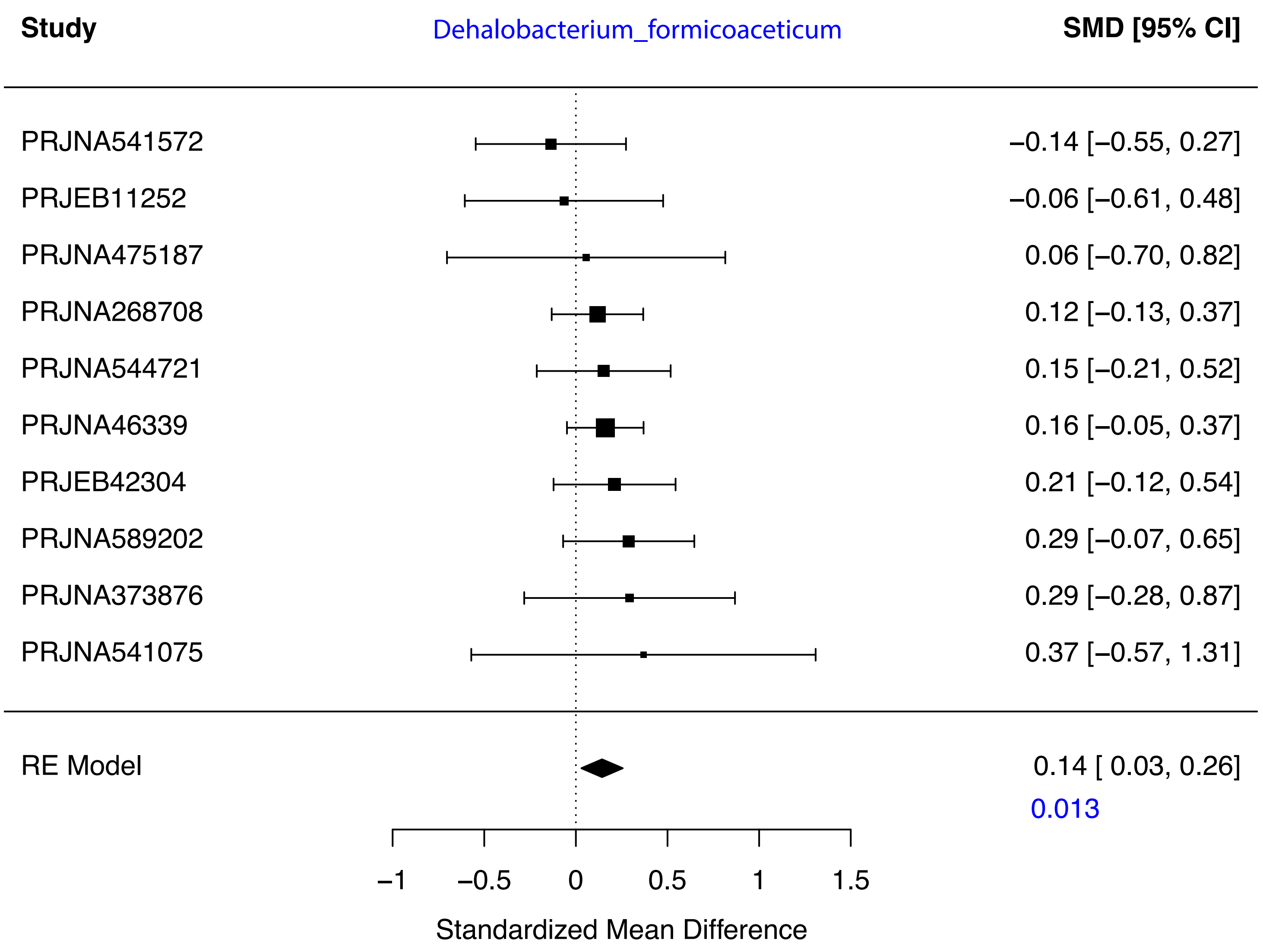
Study
Dehalobacterium_formicoaceticum
SMD [95% CI]
PRJNA541572 −0.14 [−0.55, 0.27]
PRJEB11252 −0.06 [−0.61, 0.48]
PRJNA475187 0.06 [−0.70, 0.82]
PRJNA268708 0.12 [−0.13, 0.37]
PRJNA544721 0.15 [−0.21, 0.52]
PRJNA46339 0.16 [−0.05, 0.37]
PRJEB42304 0.21 [−0.12, 0.54]
PRJNA589202 0.29 [−0.07, 0.65]
PRJNA373876 0.29 [−0.28, 0.87]
PRJNA541075 0.37 [−0.57, 1.31]
RE Model 0.14 [0.03, 0.26]
0.013
−1
−0.5
0
0.5
1
1.5
Standardized Mean Difference

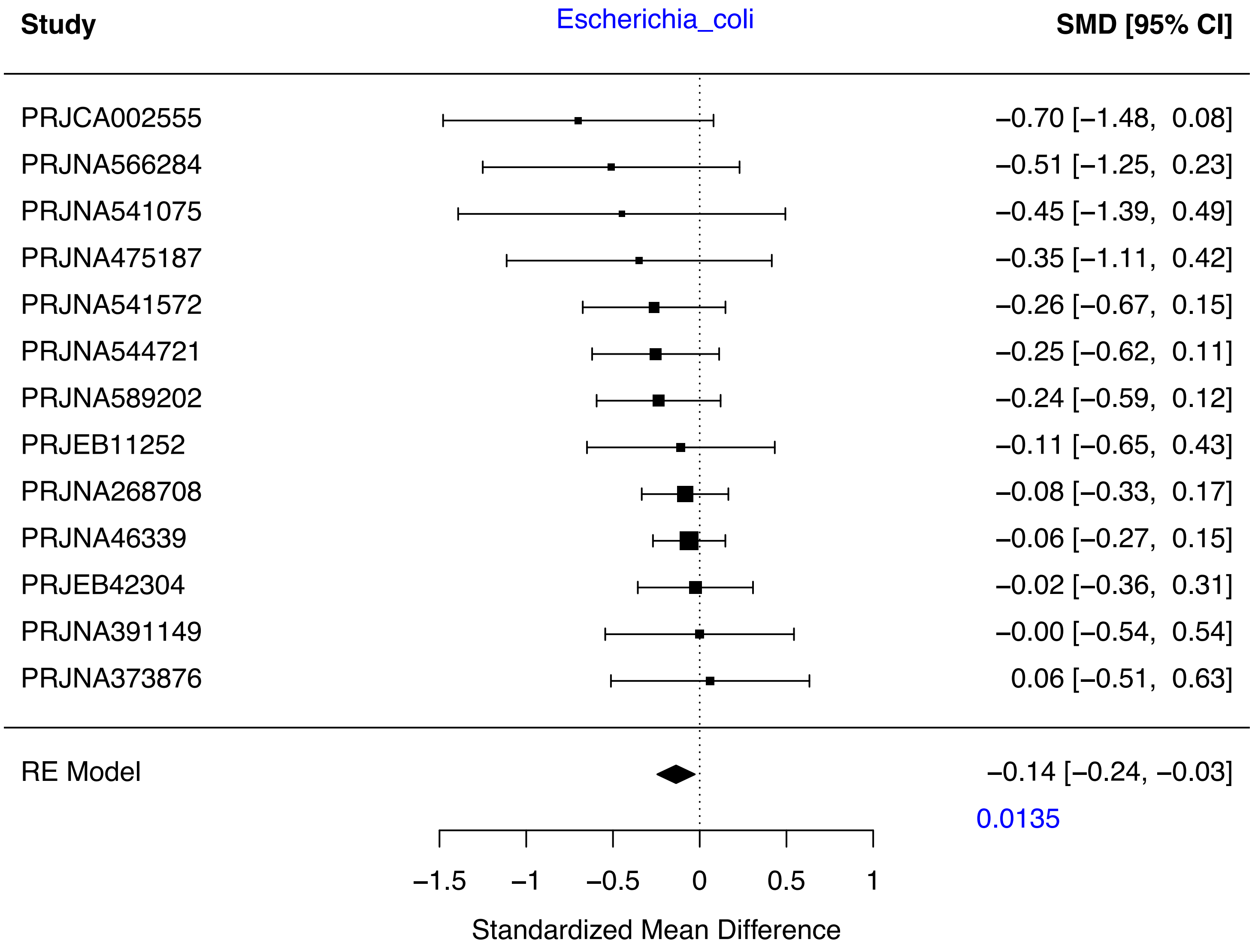
Study
Escherichia_coli
SMD [95% CI]
PRJCA002555 −0.70 [−1.48, 0.08]
PRJNA566284 −0.51 [−1.25, 0.23]
PRJNA541075 −0.45 [−1.39, 0.49]
PRJNA475187 −0.35 [−1.11, 0.42]
PRJNA541572 −0.26 [−0.67, 0.15]
PRJNA544721 −0.25 [−0.62, 0.11]
PRJNA589202 −0.24 [−0.59, 0.12]
PRJEB11252 −0.11 [−0.65, 0.43]
PRJNA268708 −0.08 [−0.33, 0.17]
PRJNA46339 −0.06 [−0.27, 0.15]
PRJEB42304 −0.02 [−0.36, 0.31]
PRJNA391149 −0.00 [−0.54, 0.54]
PRJNA373876 0.06 [−0.51, 0.63]
RE Model −0.14 [−0.24, −0.03]
0.0135
−1.5
−1
−0.5
0
0.5
1
Standardized Mean Difference

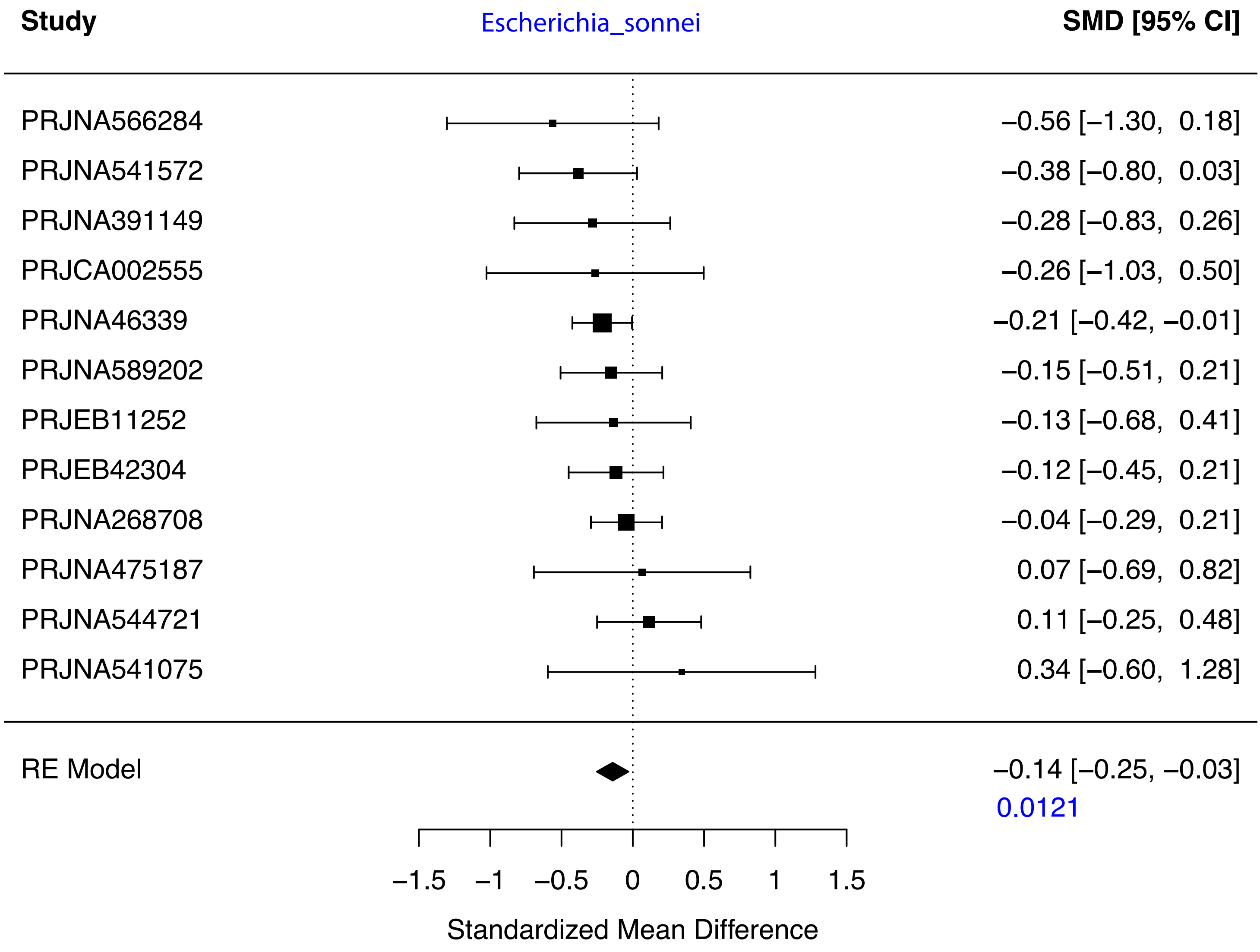
Study
Escherichia_sonnei
SMD [95% CI]
PRJNA566284 −0.56 [−1.30, 0.18]
PRJNA541572 −0.38 [−0.80, 0.03]
PRJNA391149 −0.28 [−0.83, 0.26]
PRJCA002555 −0.26 [−1.03, 0.50]
PRJNA46339 −0.21 [−0.42, −0.01]
PRJNA589202 −0.15 [−0.51, 0.21]
PRJEB11252 −0.13 [−0.68, 0.41]
PRJEB42304 −0.12 [−0.45, 0.21]
PRJNA268708 −0.04 [−0.29, 0.21]
PRJNA475187 0.07 [−0.69, 0.82]
PRJNA544721 0.11 [−0.25, 0.48]
PRJNA541075 0.34 [−0.60, 1.28]
RE Model −0.14 [−0.25, −0.03]
0.0121
−1.5
−1
−0.5
0
0.5
1
1.5
Standardized Mean Difference

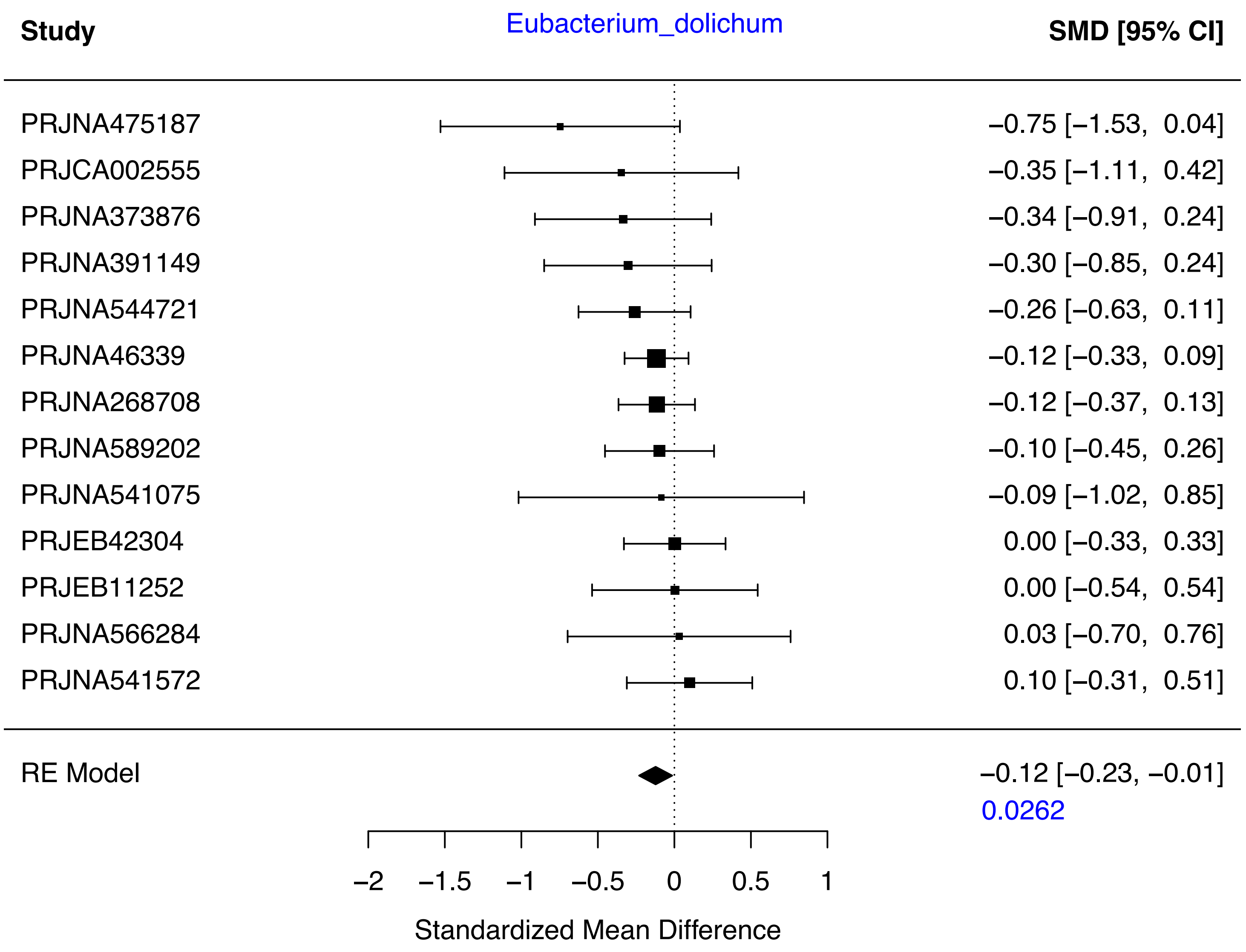
Study
Eubacterium_dolichum
SMD [95% CI]
PRJNA475187 −0.75 [−1.53, 0.04]
PRJCA002555 −0.35 [−1.11, 0.42]
PRJNA373876 −0.34 [−0.91, 0.24]
PRJNA391149 −0.30 [−0.85, 0.24]
PRJNA544721 −0.26 [−0.63, 0.11]
PRJNA46339 −0.12 [−0.33, 0.09]
PRJNA268708 −0.12 [−0.37, 0.13]
PRJNA589202 −0.10 [−0.45, 0.26]
PRJNA541075 −0.09 [−1.02, 0.85]
PRJEB42304 0.00 [−0.33, 0.33]
PRJEB11252 0.00 [−0.54, 0.54]
PRJNA566284 0.03 [−0.70, 0.76]
PRJNA541572 0.10 [−0.31, 0.51]
RE Model −0.12 [−0.23, −0.01]
0.0262
−2
−1.5
−1
−0.5
0
0.5
1
Standardized Mean Difference

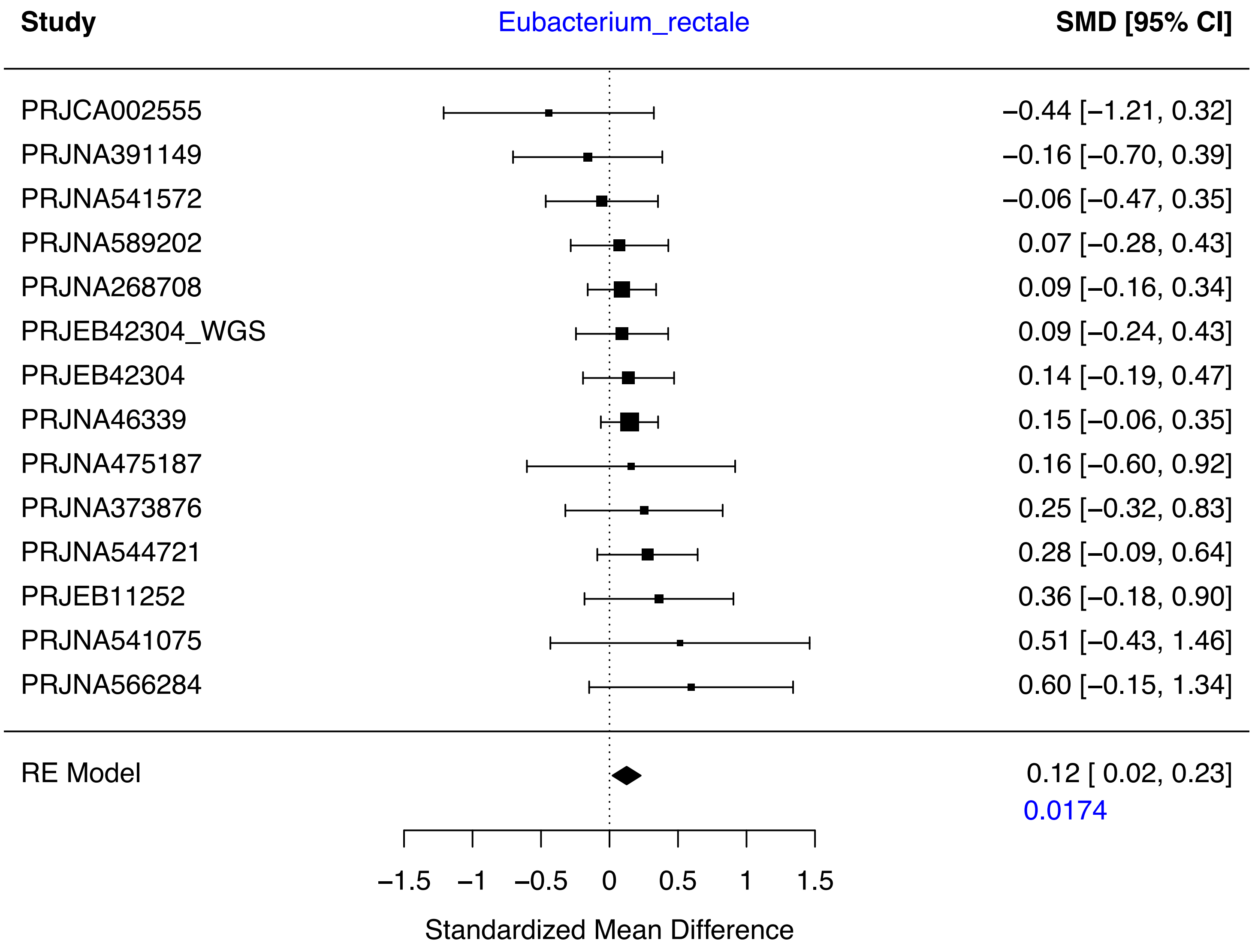
Study
Eubacterium_rectale
SMD [95% CI]
PRJCA002555 −0.44 [−1.21, 0.32]
PRJNA391149 −0.16 [−0.70, 0.39]
PRJNA541572 −0.06 [−0.47, 0.35]
PRJNA589202 0.07 [−0.28, 0.43]
PRJNA268708 0.09 [−0.16, 0.34]
PRJEB42304_WGS 0.09 [−0.24, 0.43]
PRJEB42304 0.14 [−0.19, 0.47]
PRJNA46339 0.15 [−0.06, 0.35]
PRJNA475187 0.16 [−0.60, 0.92]
PRJNA373876 0.25 [−0.32, 0.83]
PRJNA544721 0.28 [−0.09, 0.64]
PRJEB11252 0.36 [−0.18, 0.90]
PRJNA541075 0.51 [−0.43, 1.46]
PRJNA566284 0.60 [−0.15, 1.34]
RE Model 0.12 [0.02, 0.23]
0.0174
−1.5
−1
−0.5
0
0.5
1
1.5
Standardized Mean Difference

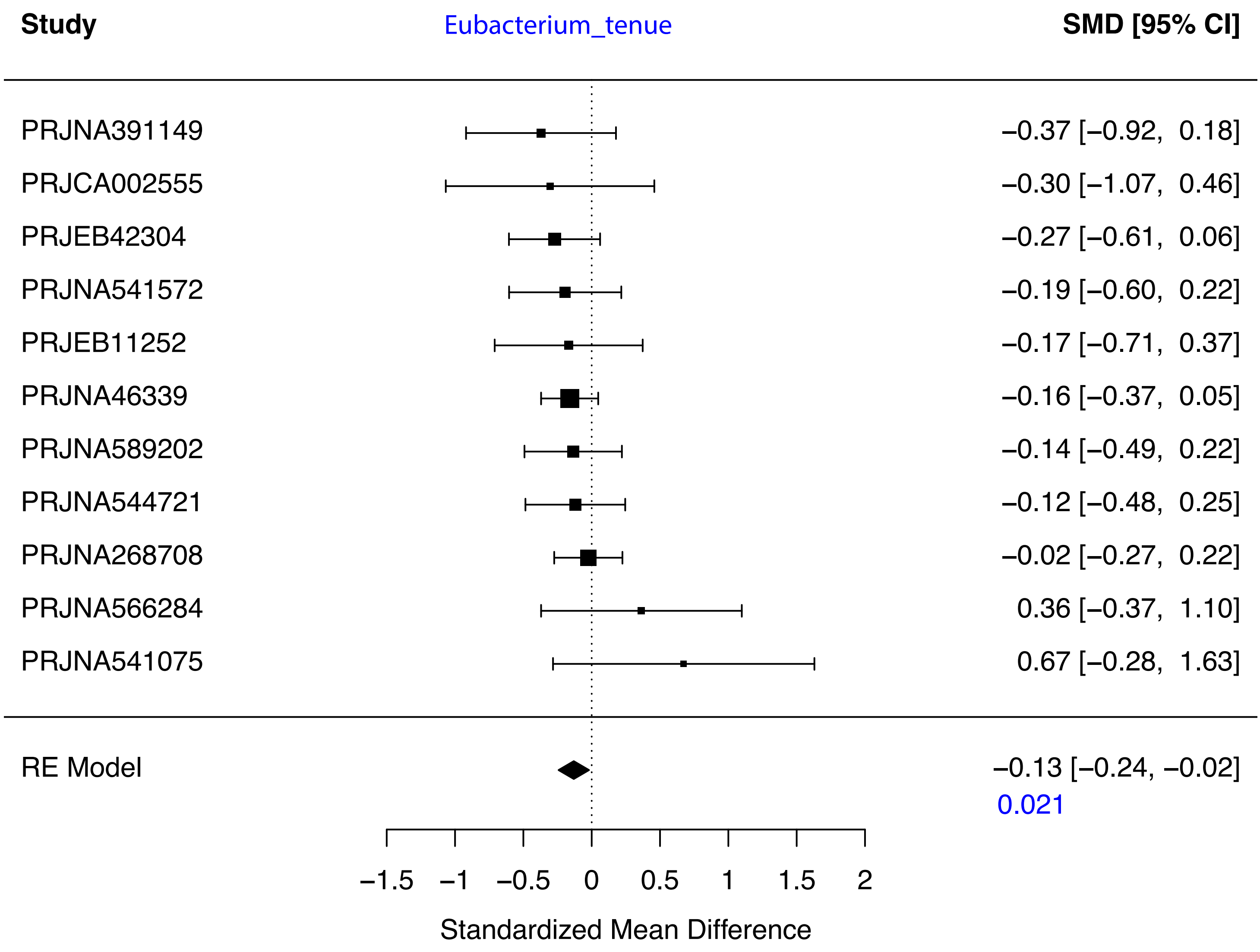

Study
Eubacterium_tenue
SMD [95% CI]
PRJNA391149 −0.37 [−0.92, 0.18]
PRJCA002555 −0.30 [−1.07, 0.46]
PRJEB42304 −0.27 [−0.61, 0.06]
PRJNA541572 −0.19 [−0.60, 0.22]
PRJEB11252 −0.17 [−0.71, 0.37]
PRJNA46339 −0.16 [−0.37, 0.05]
PRJNA589202 −0.14 [−0.49, 0.22]
PRJNA544721 −0.12 [−0.48, 0.25]
PRJNA268708 −0.02 [−0.27, 0.22]
PRJNA566284 0.36 [−0.37, 1.10]
PRJNA541075 0.67 [−0.28, 1.63]
RE Model −0.13 [−0.24, −0.02]
0.021
−1.5 −1 −0.5 0 0.5 1 1.5 2
Standardized Mean Difference

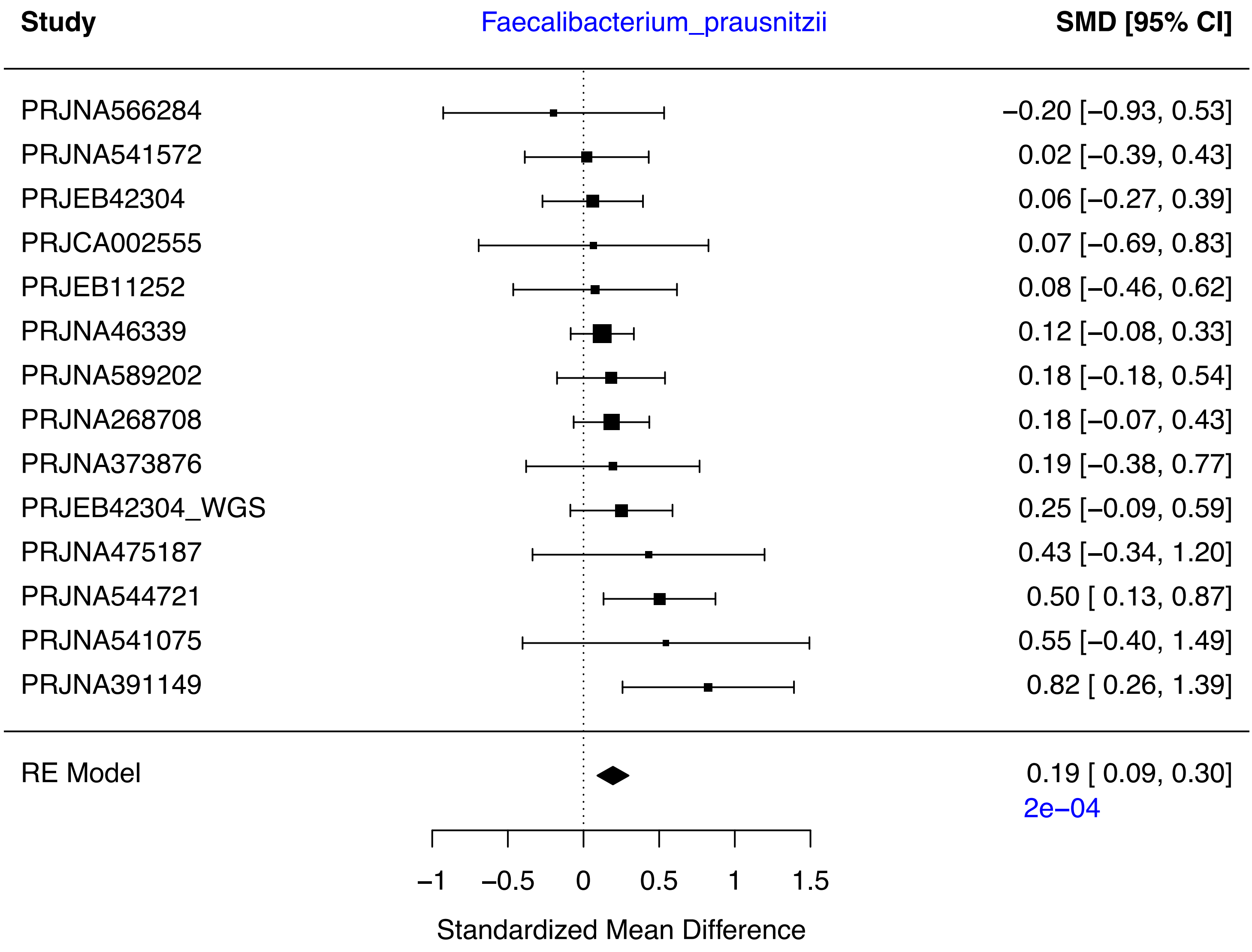
Study
Faecalibacterium_prausnitzii
SMD [95% CI]
PRJNA566284 −0.20 [−0.93, 0.53]
PRJNA541572 0.02 [−0.39, 0.43]
PRJEB42304 0.06 [−0.27, 0.39]
PRJCA002555 0.07 [−0.69, 0.83]
PRJEB11252 0.08 [−0.46, 0.62]
PRJNA46339 0.12 [−0.08, 0.33]
PRJNA589202 0.18 [−0.18, 0.54]
PRJNA268708 0.18 [−0.07, 0.43]
PRJNA373876 0.19 [−0.38, 0.77]
PRJEB42304_WGS 0.25 [−0.09, 0.59]
PRJNA475187 0.43 [−0.34, 1.20]
PRJNA544721 0.50 [0.13, 0.87]
PRJNA541075 0.55 [−0.40, 1.49]
PRJNA391149 0.82 [0.26, 1.39]
RE Model 0.19 [0.09, 0.30]
2e−04
−1
−0.5
0
0.5
1
1.5
Standardized Mean Difference

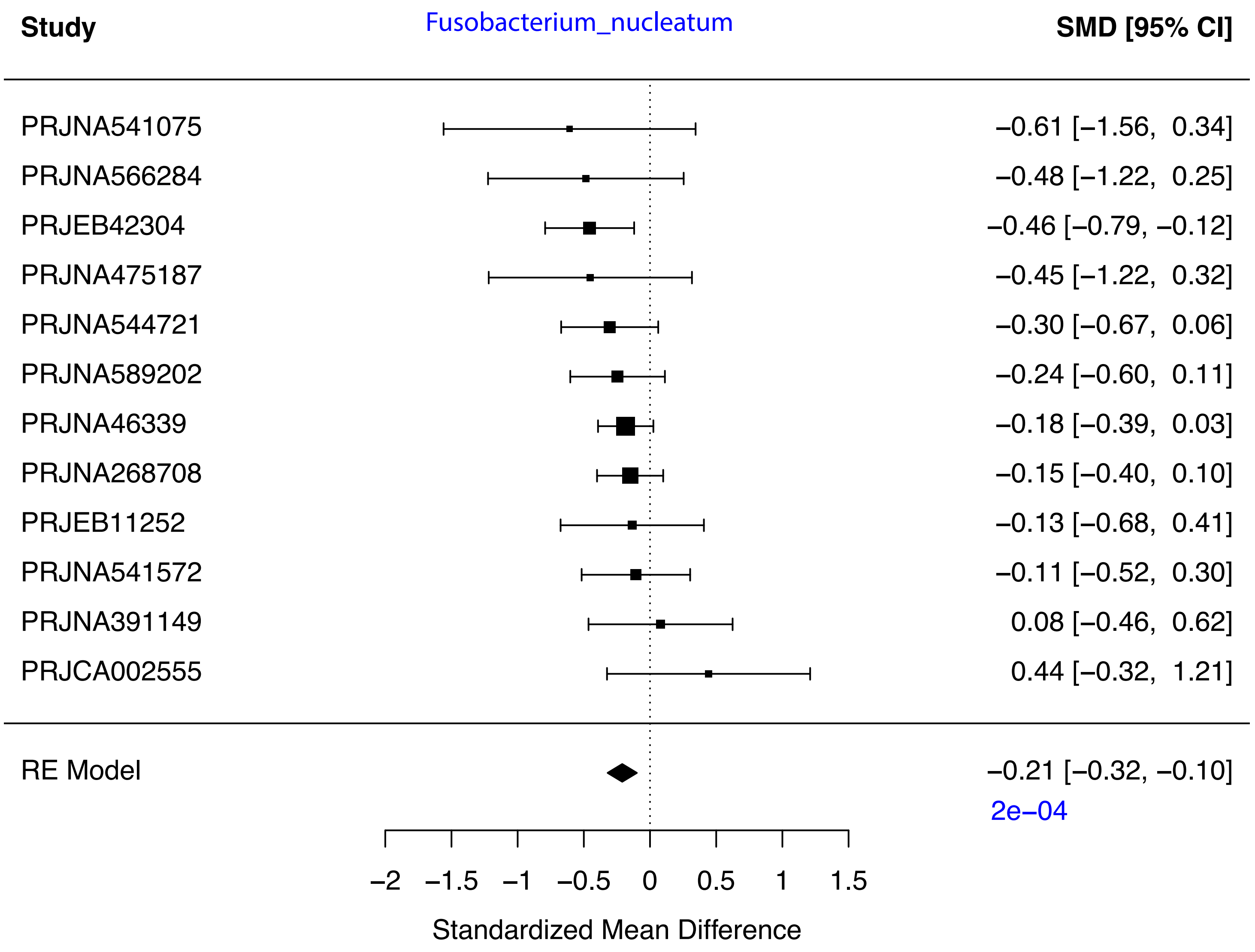
Study
Fusobacterium_nucleatum
SMD [95% CI]
PRJNA541075 −0.61 [−1.56, 0.34]
PRJNA566284 −0.48 [−1.22, 0.25]
PRJEB42304 −0.46 [−0.79, −0.12]
PRJNA475187 −0.45 [−1.22, 0.32]
PRJNA544721 −0.30 [−0.67, 0.06]
PRJNA589202 −0.24 [−0.60, 0.11]
PRJNA46339 −0.18 [−0.39, 0.03]
PRJNA268708 −0.15 [−0.40, 0.10]
PRJEB11252 −0.13 [−0.68, 0.41]
PRJNA541572 −0.11 [−0.52, 0.30]
PRJNA391149 0.08 [−0.46, 0.62]
PRJCA002555 0.44 [−0.32, 1.21]
RE Model −0.21 [−0.32, −0.10]
2e−04
−2
−1.5
−1
−0.5
0
0.5
1
1.5
Standardized Mean Difference

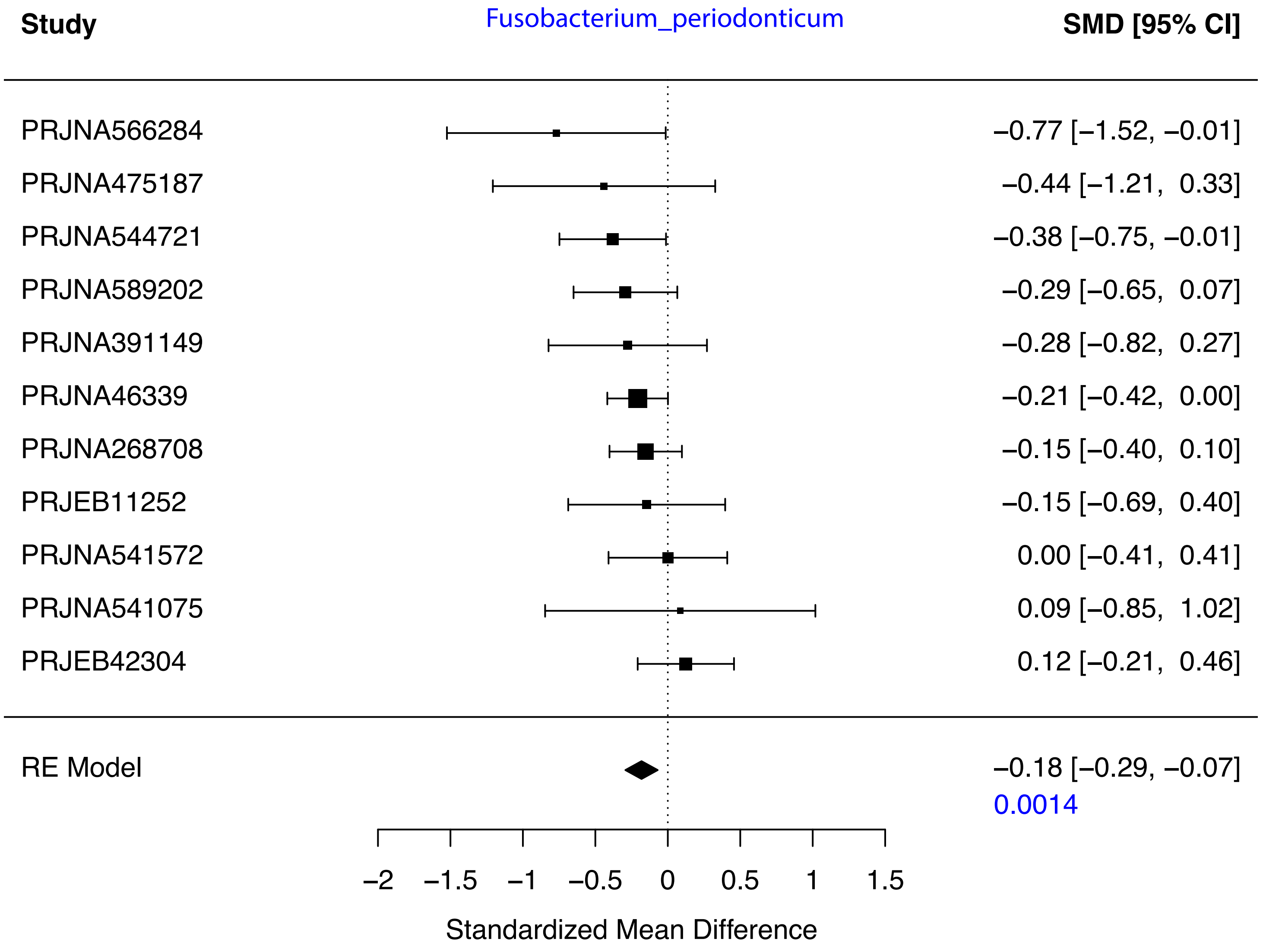
Study
Fusobacterium_periodonticum
SMD [95% CI]
PRJNA566284 −0.77 [−1.52, −0.01]
PRJNA475187 −0.44 [−1.21, 0.33]
PRJNA544721 −0.38 [−0.75, −0.01]
PRJNA589202 −0.29 [−0.65, 0.07]
PRJNA391149 −0.28 [−0.82, 0.27]
PRJNA46339 −0.21 [−0.42, 0.00]
PRJNA268708 −0.15 [−0.40, 0.10]
PRJEB11252 −0.15 [−0.69, 0.40]
PRJNA541572 0.00 [−0.41, 0.41]
PRJNA541075 0.09 [−0.85, 1.02]
PRJEB42304 0.12 [−0.21, 0.46]
RE Model −0.18 [−0.29, −0.07]
0.0014
−2
−1.5
−1
−0.5
0
0.5
1
1.5
Standardized Mean Difference

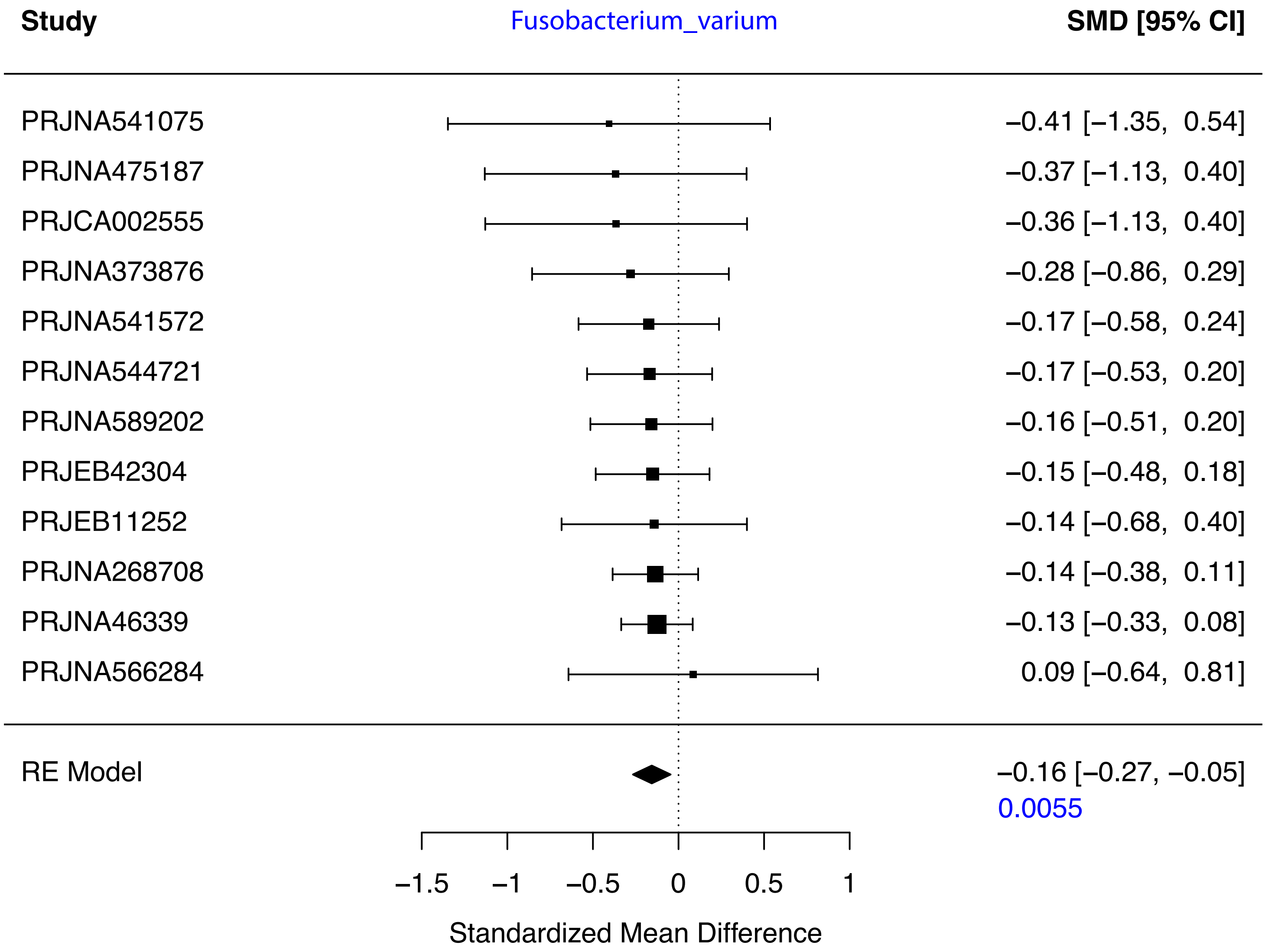
Study
Fusobacterium_varium
SMD [95% CI]
PRJNA541075
–0.41 [–1.35, 0.54]
PRJNA475187
–0.37 [–1.13, 0.40]
PRJCA002555
–0.36 [–1.13, 0.40]
PRJNA373876
–0.28 [–0.86, 0.29]
PRJNA541572
–0.17 [–0.58, 0.24]
PRJNA544721
–0.17 [–0.53, 0.20]
PRJNA589202
–0.16 [–0.51, 0.20]
PRJEB42304
–0.15 [–0.48, 0.18]
PRJEB11252
–0.14 [–0.68, 0.40]
PRJNA268708
–0.14 [–0.38, 0.11]
PRJNA46339
–0.13 [–0.33, 0.08]
PRJNA566284
0.09 [–0.64, 0.81]
RE Model
–0.16 [–0.27, –0.05]
0.0055
–1.5
–1
–0.5
0
0.5
1
Standardized Mean Difference

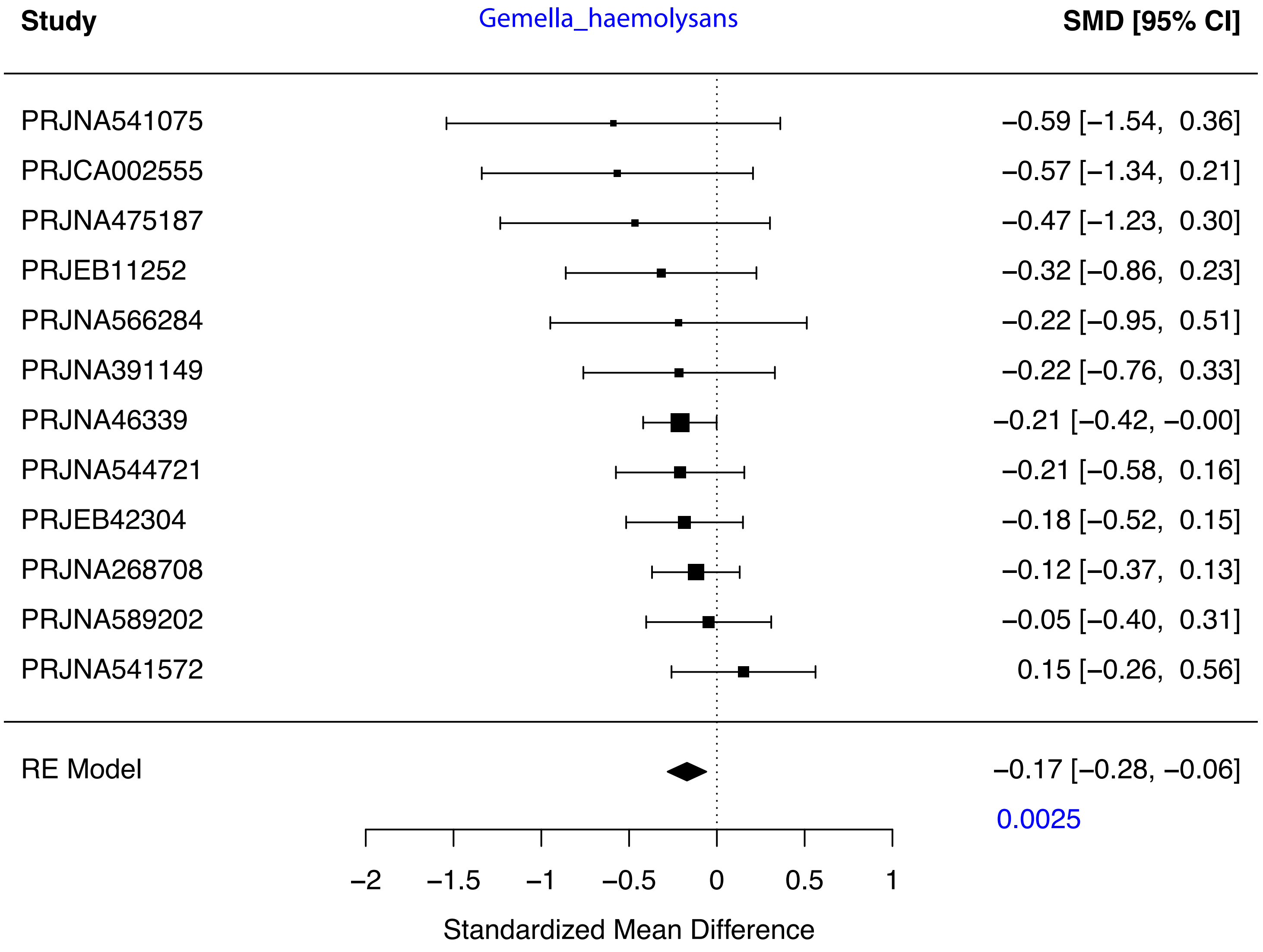
Study
Gemella_haemolysans
SMD [95% CI]
PRJNA541075 −0.59 [−1.54, 0.36]
PRJCA002555 −0.57 [−1.34, 0.21]
PRJNA475187 −0.47 [−1.23, 0.30]
PRJEB11252 −0.32 [−0.86, 0.23]
PRJNA566284 −0.22 [−0.95, 0.51]
PRJNA391149 −0.22 [−0.76, 0.33]
PRJNA46339 −0.21 [−0.42, −0.00]
PRJNA544721 −0.21 [−0.58, 0.16]
PRJEB42304 −0.18 [−0.52, 0.15]
PRJNA268708 −0.12 [−0.37, 0.13]
PRJNA589202 −0.05 [−0.40, 0.31]
PRJNA541572 0.15 [−0.26, 0.56]
RE Model −0.17 [−0.28, −0.06]
0.0025
−2
−1.5
−1
−0.5
0
0.5
1
Standardized Mean Difference

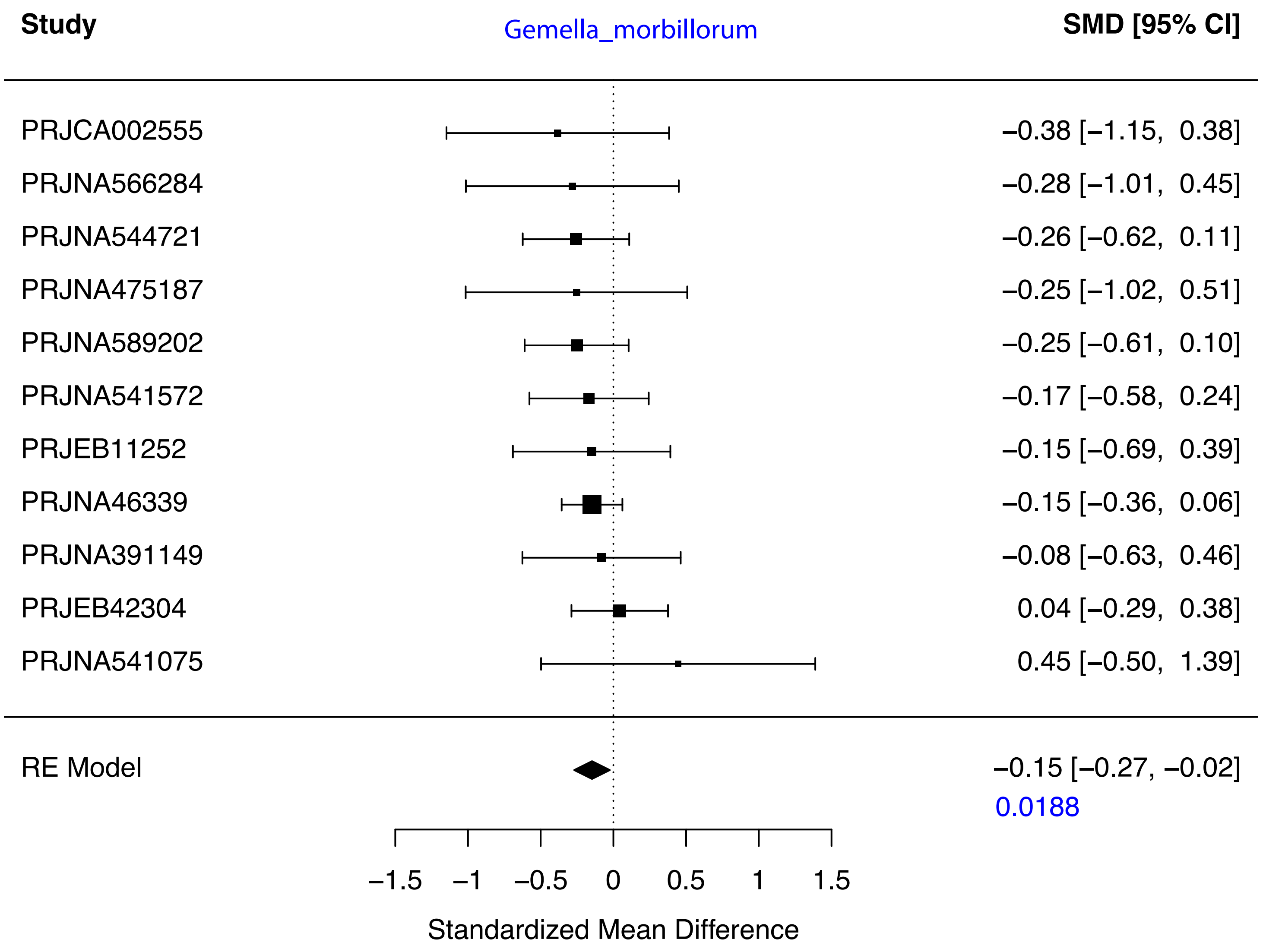
Study
Gemella_morbillorum
SMD [95% CI]
PRJCA002555
−0.38 [−1.15, 0.38]
PRJNA566284
−0.28 [−1.01, 0.45]
PRJNA544721
−0.26 [−0.62, 0.11]
PRJNA475187
−0.25 [−1.02, 0.51]
PRJNA589202
−0.25 [−0.61, 0.10]
PRJNA541572
−0.17 [−0.58, 0.24]
PRJEB11252
−0.15 [−0.69, 0.39]
PRJNA46339
−0.15 [−0.36, 0.06]
PRJNA391149
−0.08 [−0.63, 0.46]
PRJEB42304
0.04 [−0.29, 0.38]
PRJNA541075
0.45 [−0.50, 1.39]
RE Model
−0.15 [−0.27, −0.02]
0.0188
−1.5
−1
−0.5
0
0.5
1
1.5
Standardized Mean Difference

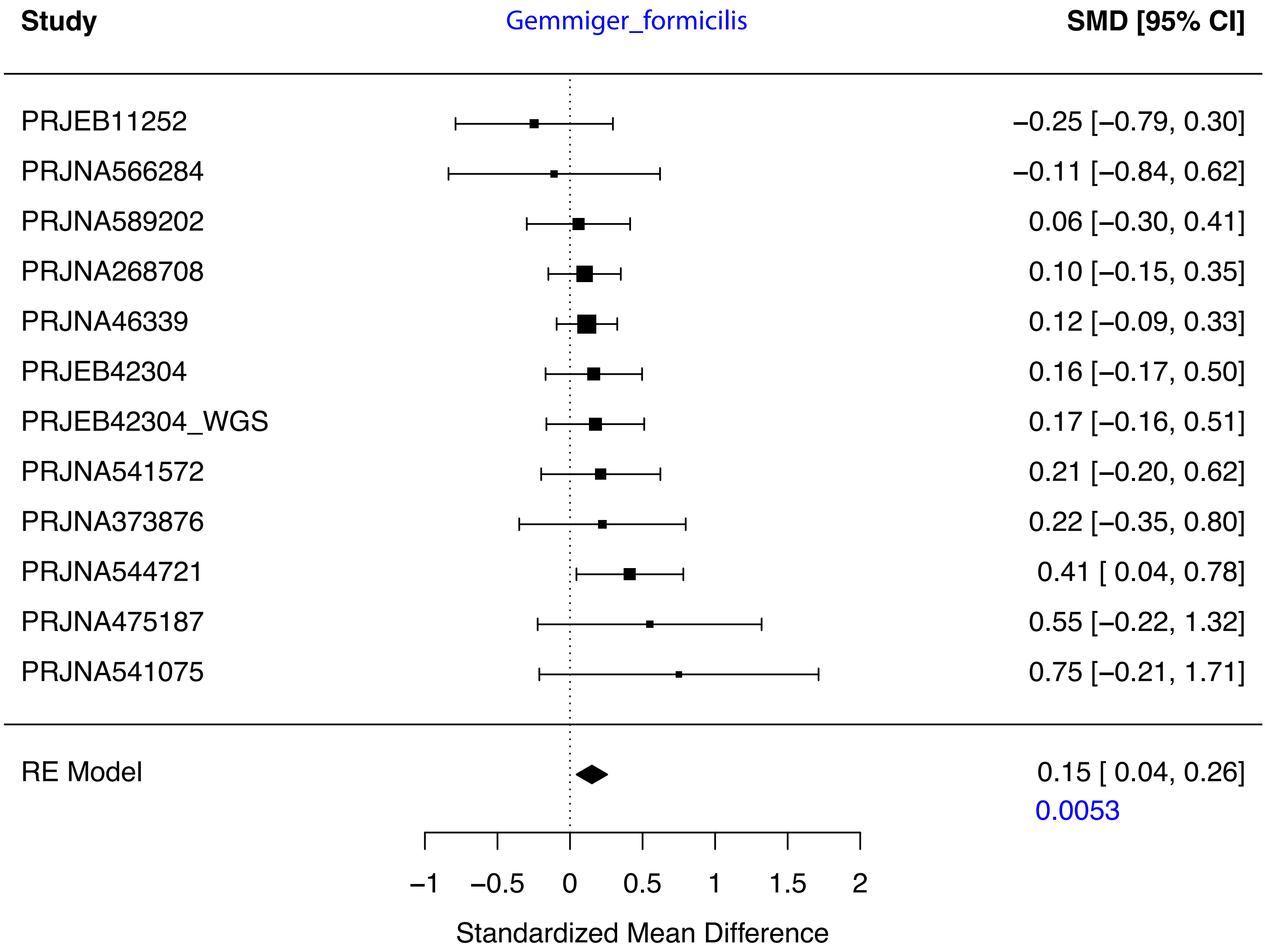
Study
Gemmiger_formicilis
SMD [95% CI]
PRJEB11252
−0.25 [−0.79, 0.30]
PRJNA566284
−0.11 [−0.84, 0.62]
PRJNA589202
0.06 [−0.30, 0.41]
PRJNA268708
0.10 [−0.15, 0.35]
PRJNA46339
0.12 [−0.09, 0.33]
PRJEB42304
0.16 [−0.17, 0.50]
PRJEB42304_WGS
0.17 [−0.16, 0.51]
PRJNA541572
0.21 [−0.20, 0.62]
PRJNA373876
0.22 [−0.35, 0.80]
PRJNA544721
0.41 [0.04, 0.78]
PRJNA475187
0.55 [−0.22, 1.32]
PRJNA541075
0.75 [−0.21, 1.71]
RE Model
0.15 [0.04, 0.26]
0.0053
−1
−0.5
0
0.5
1
1.5
2
Standardized Mean Difference

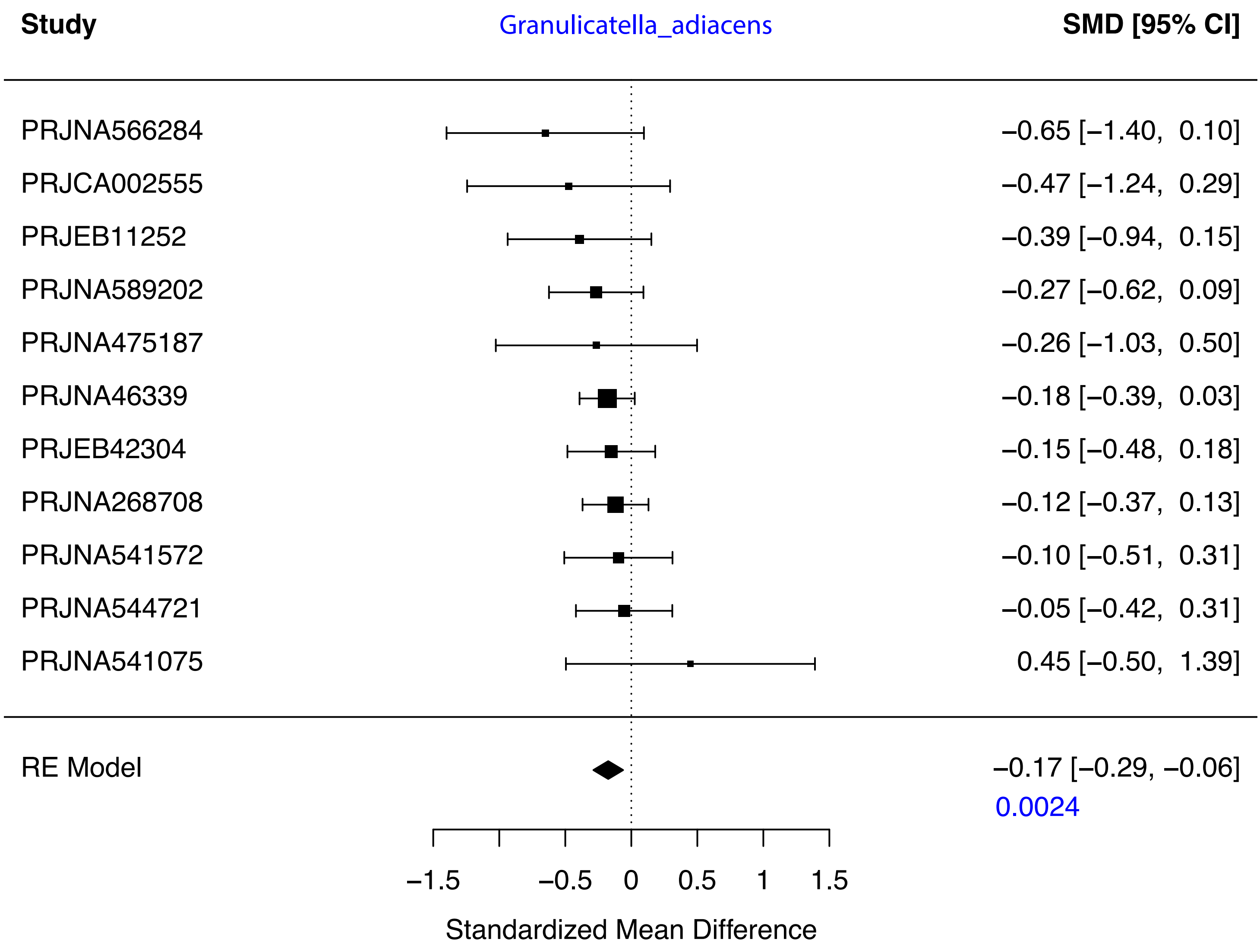

Study
Granulicatella_adiacens
SMD [95% CI]
PRJNA566284 −0.65 [−1.40, 0.10]
PRJCA002555 −0.47 [−1.24, 0.29]
PRJEB11252 −0.39 [−0.94, 0.15]
PRJNA589202 −0.27 [−0.62, 0.09]
PRJNA475187 −0.26 [−1.03, 0.50]
PRJNA46339 −0.18 [−0.39, 0.03]
PRJEB42304 −0.15 [−0.48, 0.18]
PRJNA268708 −0.12 [−0.37, 0.13]
PRJNA541572 −0.10 [−0.51, 0.31]
PRJNA544721 −0.05 [−0.42, 0.31]
PRJNA541075 0.45 [−0.50, 1.39]
RE Model −0.17 [−0.29, −0.06]
0.0024
−1.5
−0.5
0
0.5
1
1.5
Standardized Mean Difference

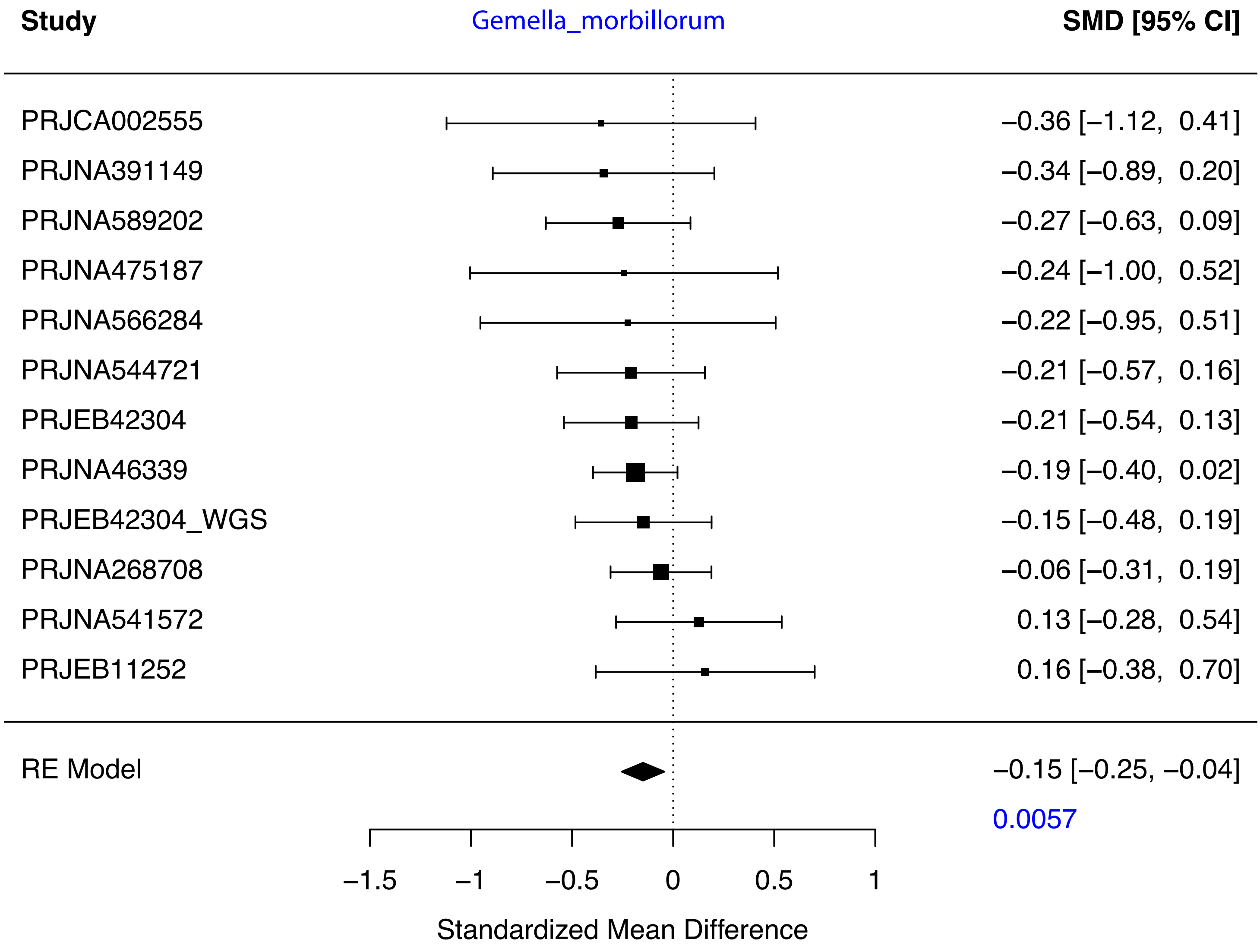
Study
Gemella_morbillorum
SMD [95% CI]
PRJCA002555 −0.36 [−1.12, 0.41]
PRJNA391149 −0.34 [−0.89, 0.20]
PRJNA589202 −0.27 [−0.63, 0.09]
PRJNA475187 −0.24 [−1.00, 0.52]
PRJNA566284 −0.22 [−0.95, 0.51]
PRJNA544721 −0.21 [−0.57, 0.16]
PRJEB42304 −0.21 [−0.54, 0.13]
PRJNA46339 −0.19 [−0.40, 0.02]
PRJEB42304_WGS −0.15 [−0.48, 0.19]
PRJNA268708 −0.06 [−0.31, 0.19]
PRJNA541572 0.13 [−0.28, 0.54]
PRJEB11252 0.16 [−0.38, 0.70]
RE Model −0.15 [−0.25, −0.04]
0.0057
−1.5
−1
−0.5
0
0.5
1
Standardized Mean Difference

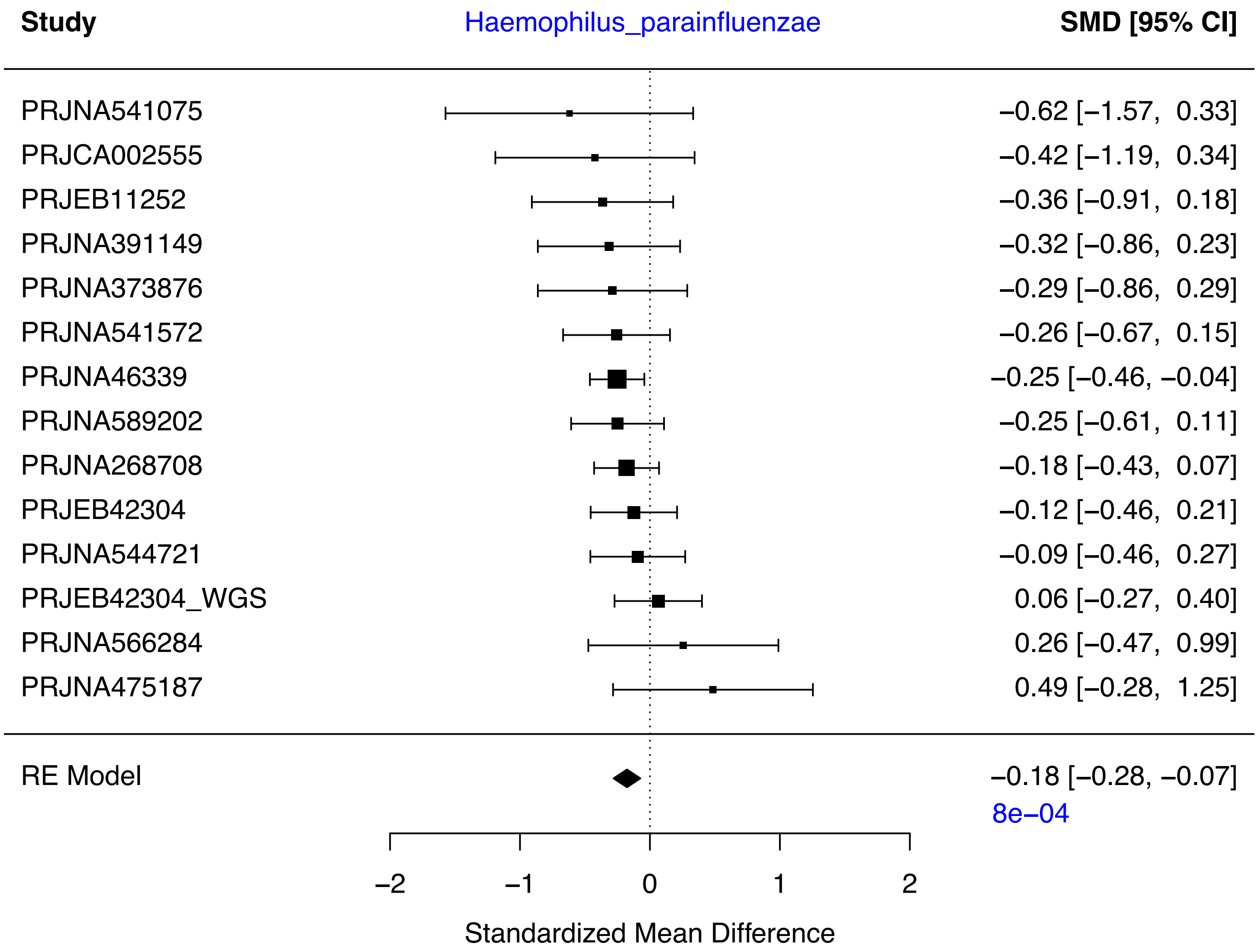
Study
Haemophilus_parainfluenzae
SMD [95% CI]
PRJNA541075 −0.62 [−1.57, 0.33]
PRJCA002555 −0.42 [−1.19, 0.34]
PRJEB11252 −0.36 [−0.91, 0.18]
PRJNA391149 −0.32 [−0.86, 0.23]
PRJNA373876 −0.29 [−0.86, 0.29]
PRJNA541572 −0.26 [−0.67, 0.15]
PRJNA46339 −0.25 [−0.46, −0.04]
PRJNA589202 −0.25 [−0.61, 0.11]
PRJNA268708 −0.18 [−0.43, 0.07]
PRJEB42304 −0.12 [−0.46, 0.21]
PRJNA544721 −0.09 [−0.46, 0.27]
PRJEB42304_WGS 0.06 [−0.27, 0.40]
PRJNA566284 0.26 [−0.47, 0.99]
PRJNA475187 0.49 [−0.28, 1.25]
RE Model −0.18 [−0.28, −0.07]
8e−04
−2
−1
0
1
2
Standardized Mean Difference

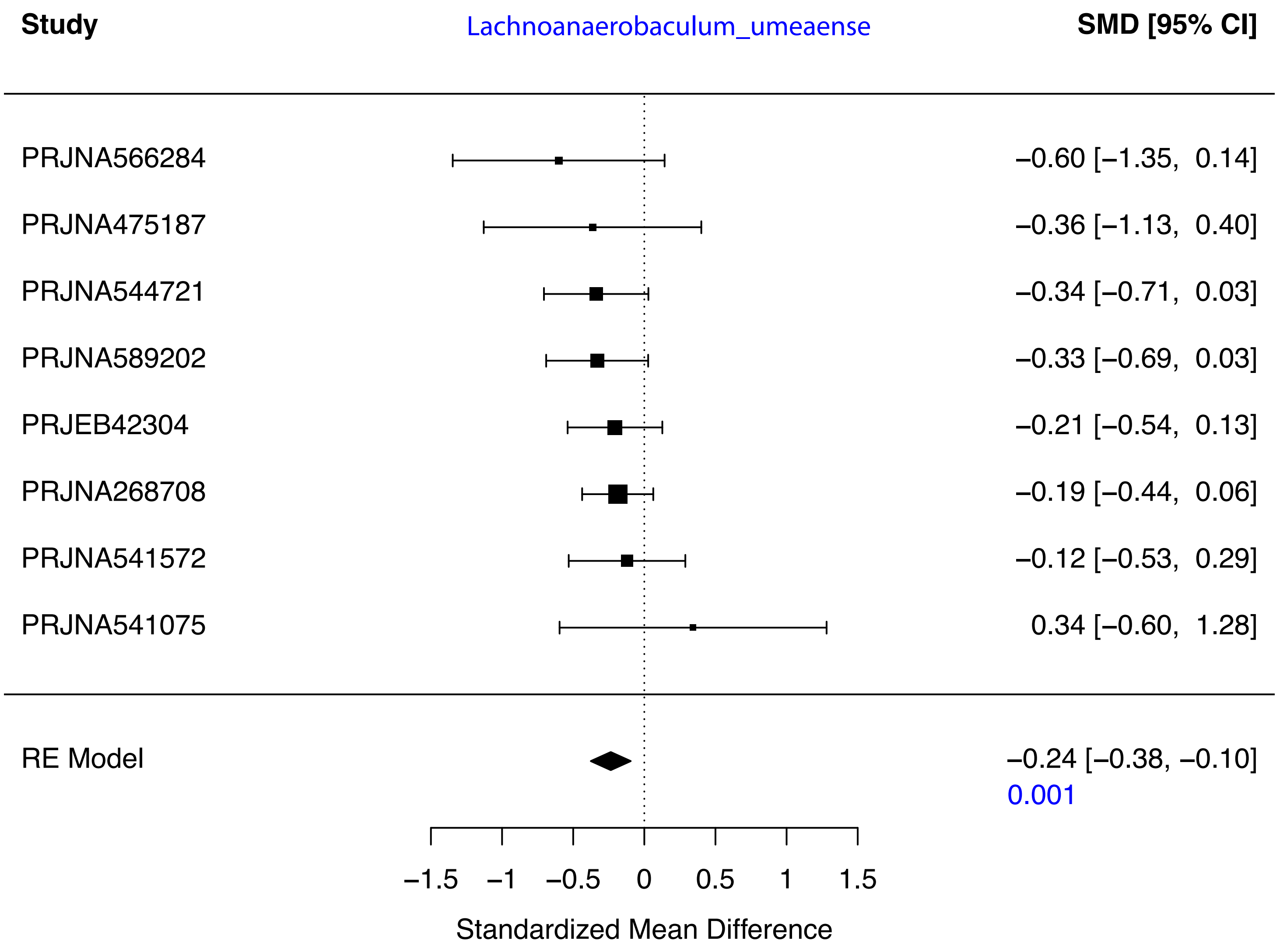
Study
Lachnoanaerobaculum_umeaense
SMD [95% CI]
PRJNA566284
−0.60 [−1.35, 0.14]
PRJNA475187
−0.36 [−1.13, 0.40]
PRJNA544721
−0.34 [−0.71, 0.03]
PRJNA589202
−0.33 [−0.69, 0.03]
PRJEB42304
−0.21 [−0.54, 0.13]
PRJNA268708
−0.19 [−0.44, 0.06]
PRJNA541572
−0.12 [−0.53, 0.29]
PRJNA541075
0.34 [−0.60, 1.28]
RE Model
−0.24 [−0.38, −0.10]
0.001
−1.5
−1
−0.5
0
0.5
1
1.5
Standardized Mean Difference

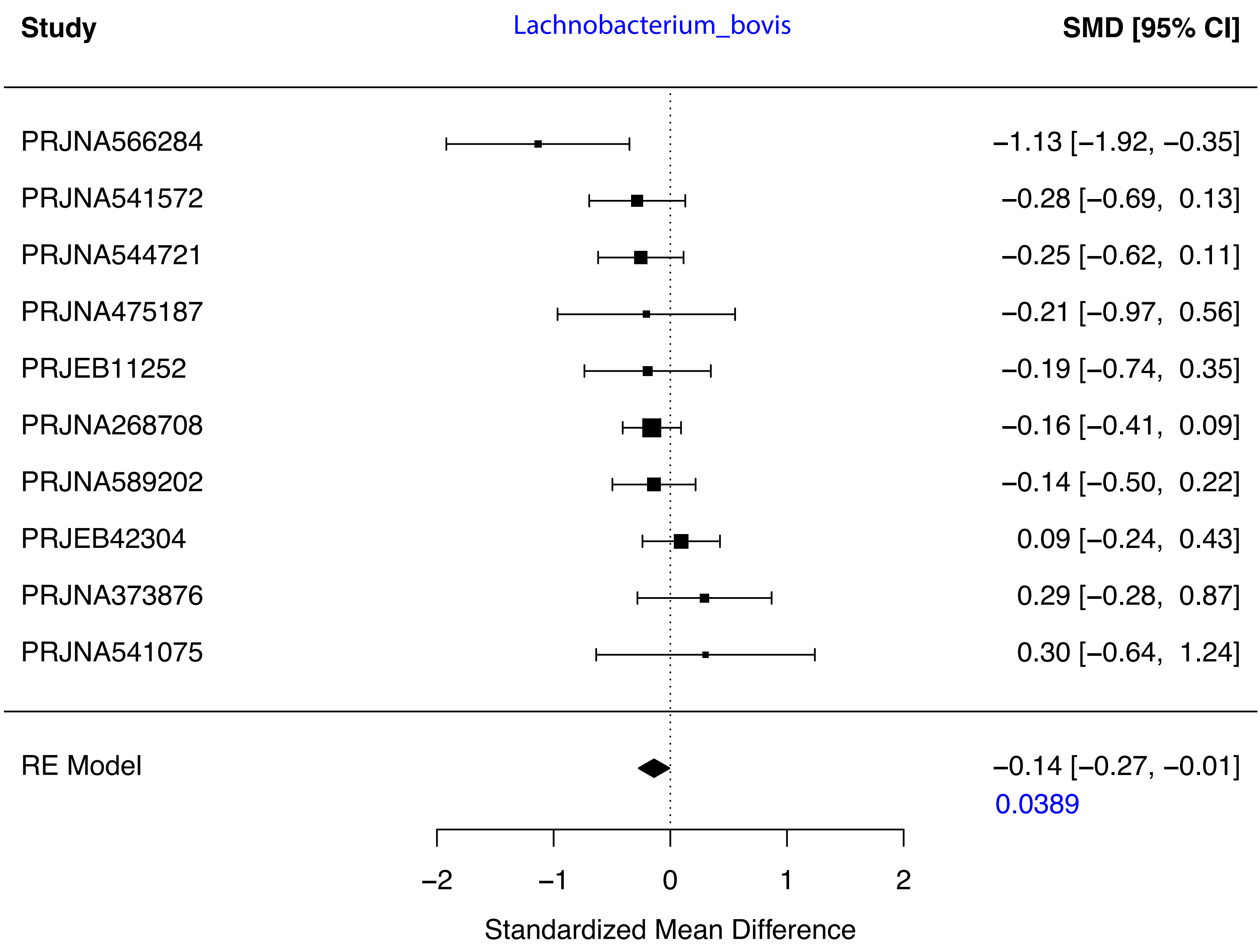
Study
Lachnobacterium_bovis
SMD [95% CI]
PRJNA566284 −1.13 [−1.92, −0.35]
PRJNA541572 −0.28 [−0.69, 0.13]
PRJNA544721 −0.25 [−0.62, 0.11]
PRJNA475187 −0.21 [−0.97, 0.56]
PRJEB11252 −0.19 [−0.74, 0.35]
PRJNA268708 −0.16 [−0.41, 0.09]
PRJNA589202 −0.14 [−0.50, 0.22]
PRJEB42304 0.09 [−0.24, 0.43]
PRJNA373876 0.29 [−0.28, 0.87]
PRJNA541075 0.30 [−0.64, 1.24]
RE Model −0.14 [−0.27, −0.01]
0.0389
−2
−1
0
1
2
Standardized Mean Difference

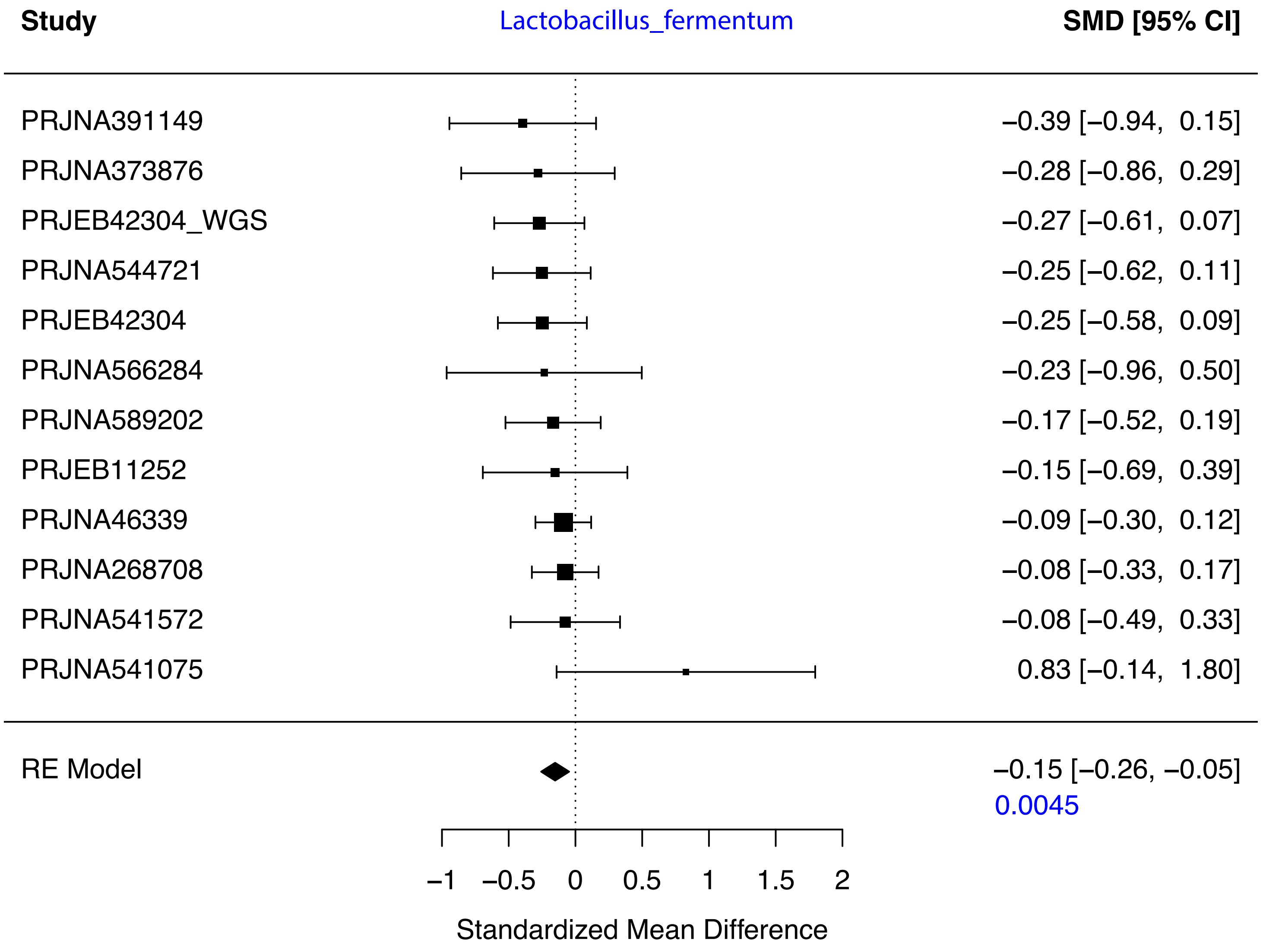
Study
Lactobacillus_fermentum
SMD [95% CI]
PRJNA391149 −0.39 [−0.94, 0.15]
PRJNA373876 −0.28 [−0.86, 0.29]
PRJEB42304_WGS −0.27 [−0.61, 0.07]
PRJNA544721 −0.25 [−0.62, 0.11]
PRJEB42304 −0.25 [−0.58, 0.09]
PRJNA566284 −0.23 [−0.96, 0.50]
PRJNA589202 −0.17 [−0.52, 0.19]
PRJEB11252 −0.15 [−0.69, 0.39]
PRJNA46339 −0.09 [−0.30, 0.12]
PRJNA268708 −0.08 [−0.33, 0.17]
PRJNA541572 −0.08 [−0.49, 0.33]
PRJNA541075 0.83 [−0.14, 1.80]
RE Model −0.15 [−0.26, −0.05]
0.0045
−1
−0.5
0
0.5
1
1.5
2
Standardized Mean Difference

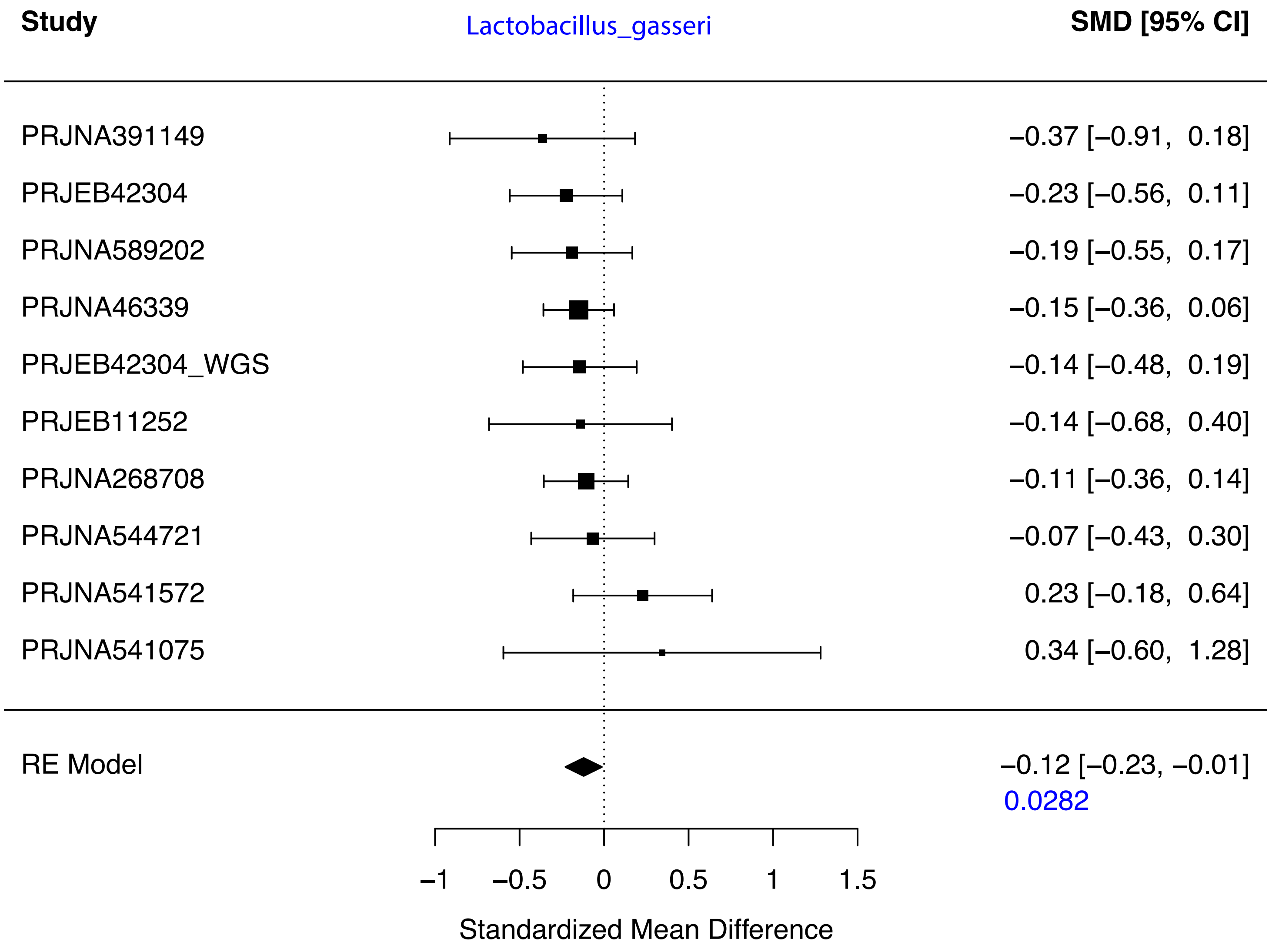
Study
Lactobacillus_gasseri
SMD [95% CI]
PRJNA391149 −0.37 [−0.91, 0.18]
PRJEB42304 −0.23 [−0.56, 0.11]
PRJNA589202 −0.19 [−0.55, 0.17]
PRJNA46339 −0.15 [−0.36, 0.06]
PRJEB42304_WGS −0.14 [−0.48, 0.19]
PRJEB11252 −0.14 [−0.68, 0.40]
PRJNA268708 −0.11 [−0.36, 0.14]
PRJNA544721 −0.07 [−0.43, 0.30]
PRJNA541572 0.23 [−0.18, 0.64]
PRJNA541075 0.34 [−0.60, 1.28]
RE Model −0.12 [−0.23, −0.01]
0.0282
−1
−0.5
0
0.5
1
1.5
Standardized Mean Difference

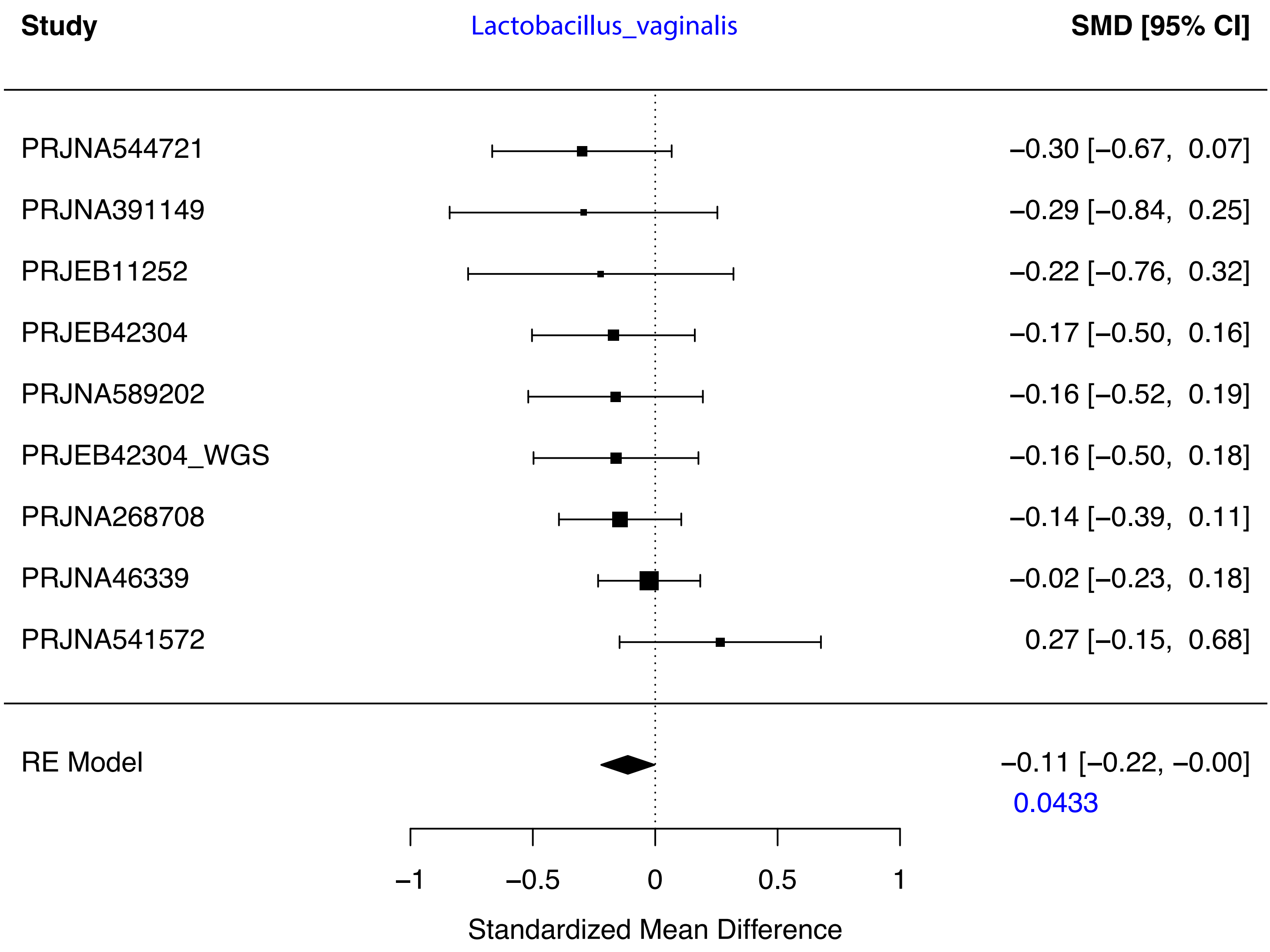
Study
Lactobacillus_vaginalis
SMD [95% CI]
PRJNA544721
−0.30 [−0.67, 0.07]
PRJNA391149
−0.29 [−0.84, 0.25]
PRJEB11252
−0.22 [−0.76, 0.32]
PRJEB42304
−0.17 [−0.50, 0.16]
PRJNA589202
−0.16 [−0.52, 0.19]
PRJEB42304_WGS
−0.16 [−0.50, 0.18]
PRJNA268708
−0.14 [−0.39, 0.11]
PRJNA46339
−0.02 [−0.23, 0.18]
PRJNA541572
0.27 [−0.15, 0.68]
RE Model
−0.11 [−0.22, −0.00]
0.0433
−1
−0.5
0
0.5
1
Standardized Mean Difference

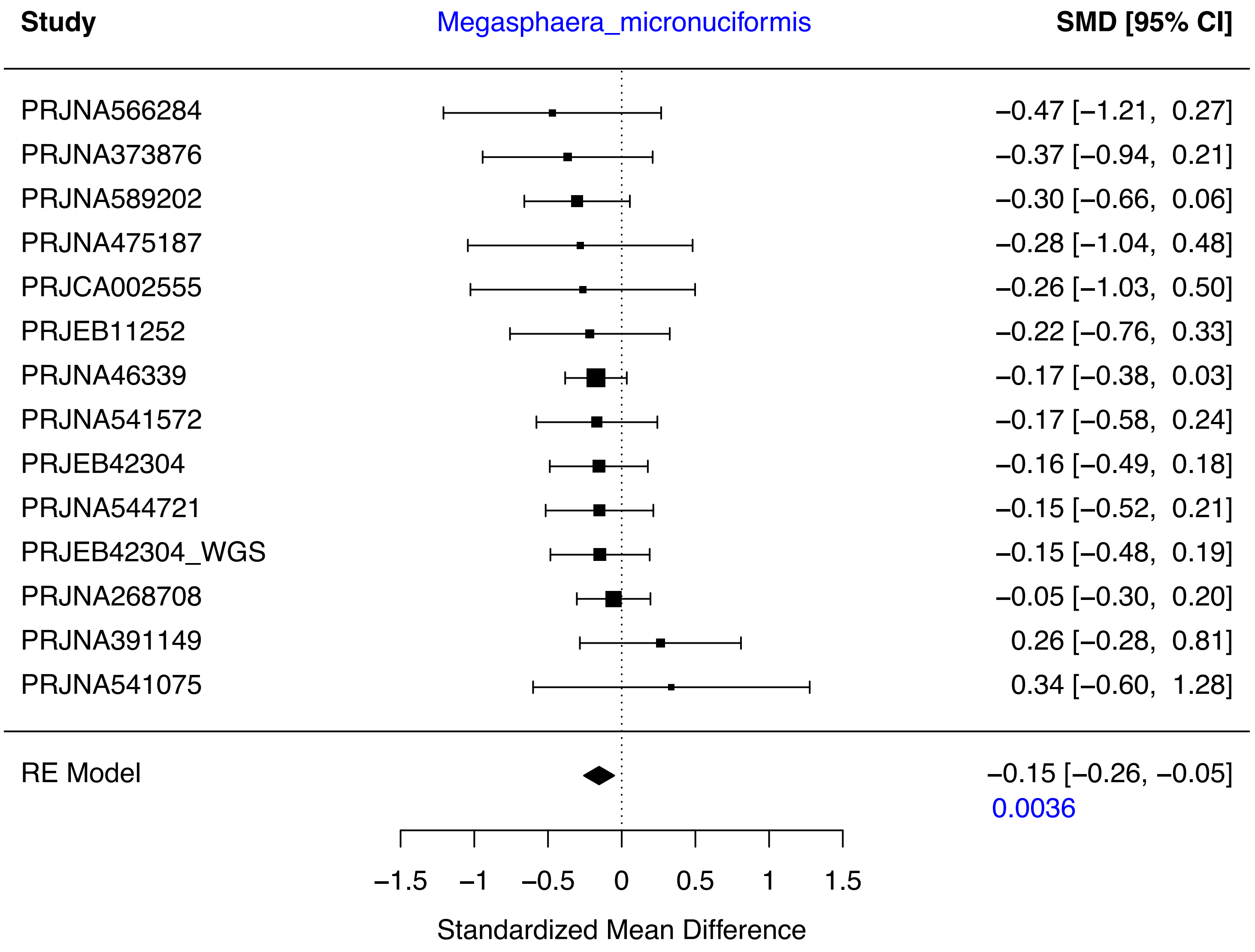
Study
Megasphaera_micronuciformis
SMD [95% CI]
PRJNA566284 −0.47 [−1.21, 0.27]
PRJNA373876 −0.37 [−0.94, 0.21]
PRJNA589202 −0.30 [−0.66, 0.06]
PRJNA475187 −0.28 [−1.04, 0.48]
PRJCA002555 −0.26 [−1.03, 0.50]
PRJEB11252 −0.22 [−0.76, 0.33]
PRJNA46339 −0.17 [−0.38, 0.03]
PRJNA541572 −0.17 [−0.58, 0.24]
PRJEB42304 −0.16 [−0.49, 0.18]
PRJNA544721 −0.15 [−0.52, 0.21]
PRJEB42304_WGS −0.15 [−0.48, 0.19]
PRJNA268708 −0.05 [−0.30, 0.20]
PRJNA391149 0.26 [−0.28, 0.81]
PRJNA541075 0.34 [−0.60, 1.28]
RE Model −0.15 [−0.26, −0.05]
0.0036
−1.5
−1
−0.5
0
0.5
1
1.5
Standardized Mean Difference

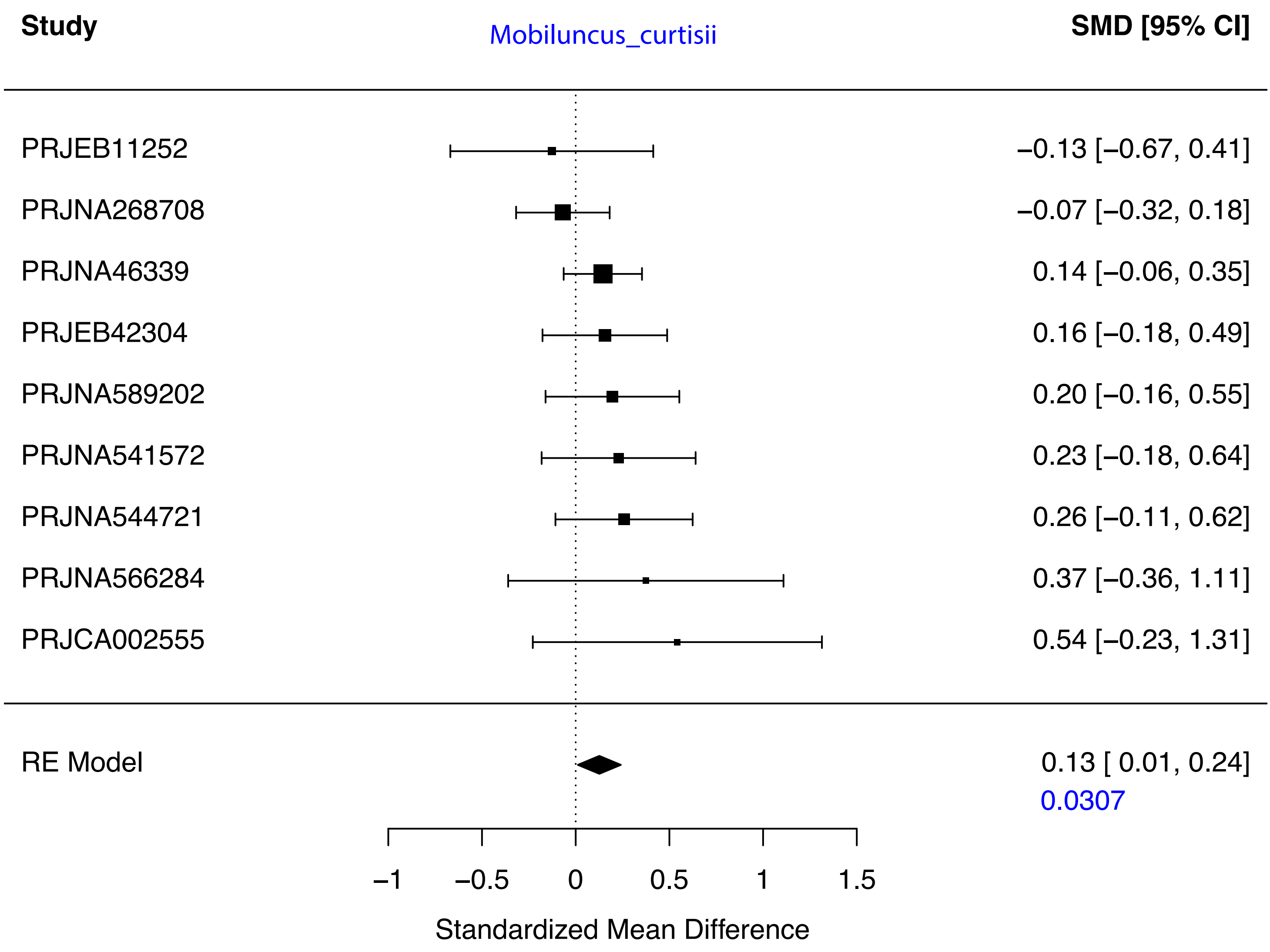
Study
Mobiluncus_curtisii
SMD [95% CI]
PRJEB11252
−0.13 [−0.67, 0.41]
PRJNA268708
−0.07 [−0.32, 0.18]
PRJNA46339
0.14 [−0.06, 0.35]
PRJEB42304
0.16 [−0.18, 0.49]
PRJNA589202
0.20 [−0.16, 0.55]
PRJNA541572
0.23 [−0.18, 0.64]
PRJNA544721
0.26 [−0.11, 0.62]
PRJNA566284
0.37 [−0.36, 1.11]
PRJCA002555
0.54 [−0.23, 1.31]
RE Model
0.13 [0.01, 0.24]
0.0307
−1
−0.5
0
0.5
1
1.5
Standardized Mean Difference

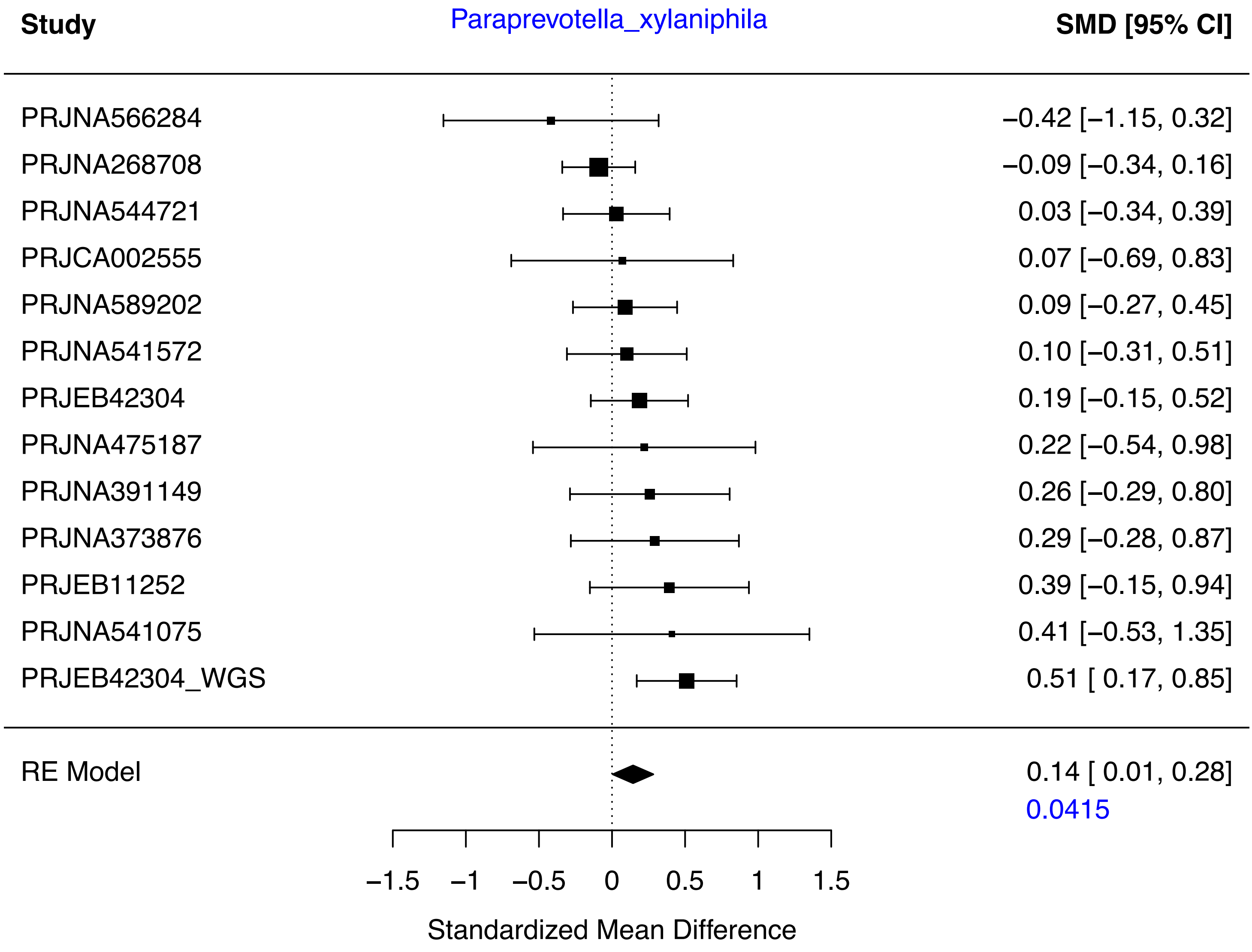
Study
Paraprevotella_xylaniphila
SMD [95% CI]
PRJNA566284 −0.42 [−1.15, 0.32]
PRJNA268708 −0.09 [−0.34, 0.16]
PRJNA544721 0.03 [−0.34, 0.39]
PRJCA002555 0.07 [−0.69, 0.83]
PRJNA589202 0.09 [−0.27, 0.45]
PRJNA541572 0.10 [−0.31, 0.51]
PRJEB42304 0.19 [−0.15, 0.52]
PRJNA475187 0.22 [−0.54, 0.98]
PRJNA391149 0.26 [−0.29, 0.80]
PRJNA373876 0.29 [−0.28, 0.87]
PRJEB11252 0.39 [−0.15, 0.94]
PRJNA541075 0.41 [−0.53, 1.35]
PRJEB42304_WGS 0.51 [0.17, 0.85]
RE Model 0.14 [0.01, 0.28]
0.0415
−1.5
−1
−0.5
0
0.5
1
1.5
Standardized Mean Difference

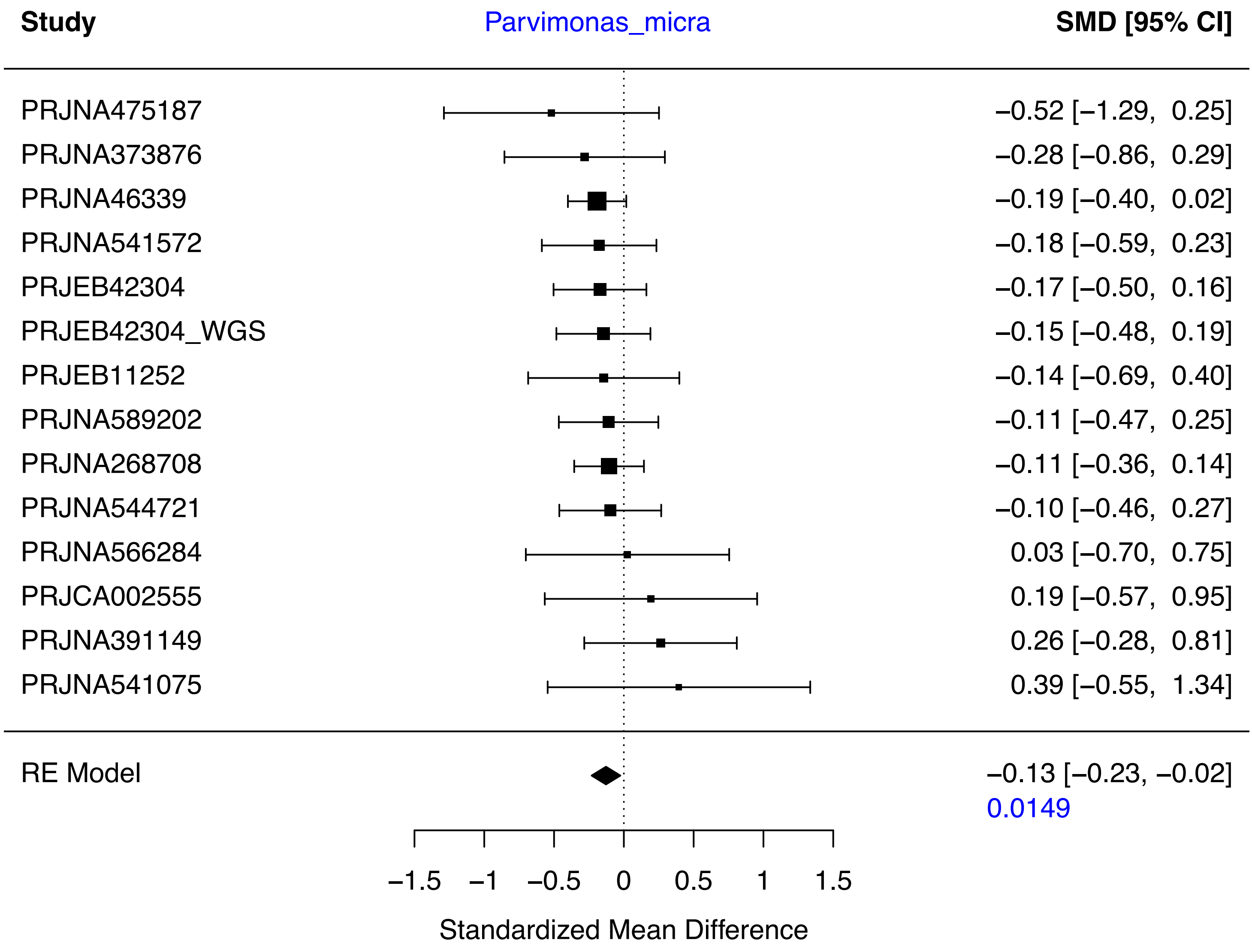
Study
Parvimonas_micra
SMD [95% CI]
PRJNA475187 −0.52 [−1.29, 0.25]
PRJNA373876 −0.28 [−0.86, 0.29]
PRJNA46339 −0.19 [−0.40, 0.02]
PRJNA541572 −0.18 [−0.59, 0.23]
PRJEB42304 −0.17 [−0.50, 0.16]
PRJEB42304_WGS −0.15 [−0.48, 0.19]
PRJEB11252 −0.14 [−0.69, 0.40]
PRJNA589202 −0.11 [−0.47, 0.25]
PRJNA268708 −0.11 [−0.36, 0.14]
PRJNA544721 −0.10 [−0.46, 0.27]
PRJNA566284 0.03 [−0.70, 0.75]
PRJCA002555 0.19 [−0.57, 0.95]
PRJNA391149 0.26 [−0.28, 0.81]
PRJNA541075 0.39 [−0.55, 1.34]
RE Model −0.13 [−0.23, −0.02]
0.0149
−1.5
−1
−0.5
0
0.5
1
1.5
Standardized Mean Difference

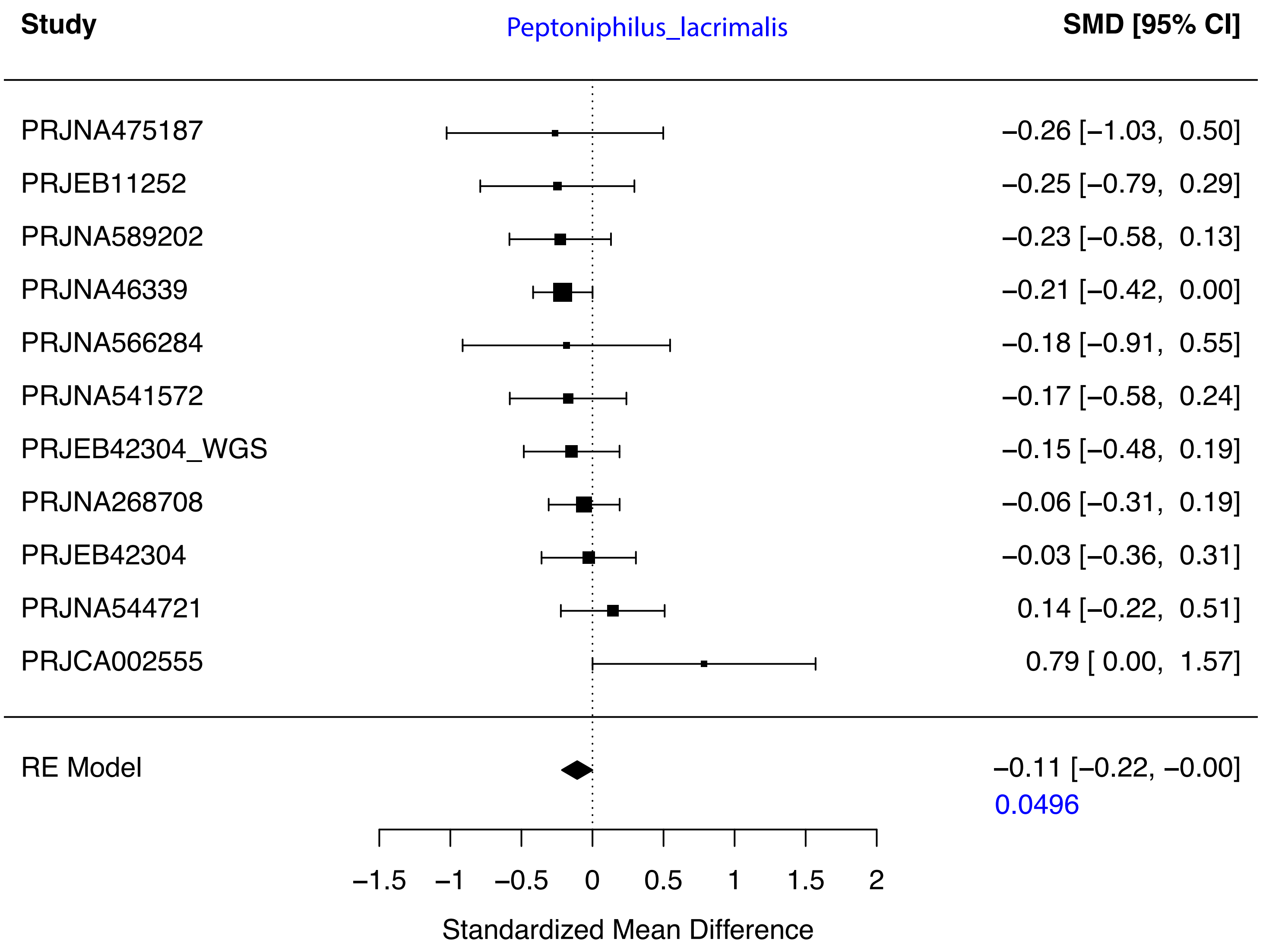
Study
Peptoniphilus_lacrimalis
SMD [95% CI]
PRJNA475187 −0.26 [−1.03, 0.50]
PRJEB11252 −0.25 [−0.79, 0.29]
PRJNA589202 −0.23 [−0.58, 0.13]
PRJNA46339 −0.21 [−0.42, 0.00]
PRJNA566284 −0.18 [−0.91, 0.55]
PRJNA541572 −0.17 [−0.58, 0.24]
PRJEB42304_WGS −0.15 [−0.48, 0.19]
PRJNA268708 −0.06 [−0.31, 0.19]
PRJEB42304 −0.03 [−0.36, 0.31]
PRJNA544721 0.14 [−0.22, 0.51]
PRJCA002555 0.79 [0.00, 1.57]
RE Model −0.11 [−0.22, −0.00]
0.0496
−1.5
−1
−0.5
0
0.5
1
1.5
2
Standardized Mean Difference

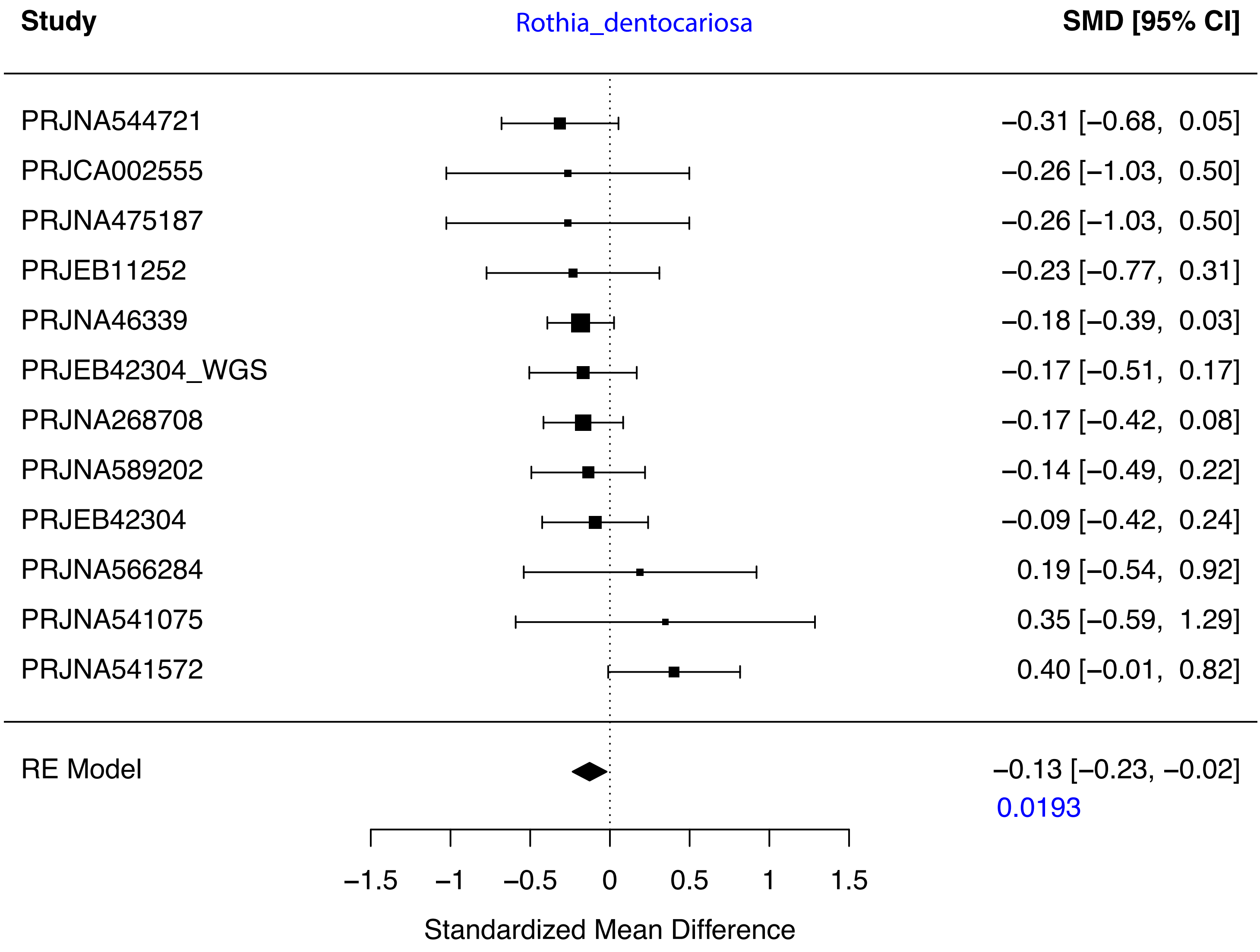

Study
Rothia_dentocariosa
SMD [95% CI]
PRJNA544721 −0.31 [−0.68, 0.05]
PRJCA002555 −0.26 [−1.03, 0.50]
PRJNA475187 −0.26 [−1.03, 0.50]
PRJEB11252 −0.23 [−0.77, 0.31]
PRJNA46339 −0.18 [−0.39, 0.03]
PRJEB42304_WGS −0.17 [−0.51, 0.17]
PRJNA268708 −0.17 [−0.42, 0.08]
PRJNA589202 −0.14 [−0.49, 0.22]
PRJEB42304 −0.09 [−0.42, 0.24]
PRJNA566284 0.19 [−0.54, 0.92]
PRJNA541075 0.35 [−0.59, 1.29]
PRJNA541572 0.40 [−0.01, 0.82]
RE Model −0.13 [−0.23, −0.02]
0.0193
−1.5
−1
−0.5
0
0.5
1
1.5
Standardized Mean Difference

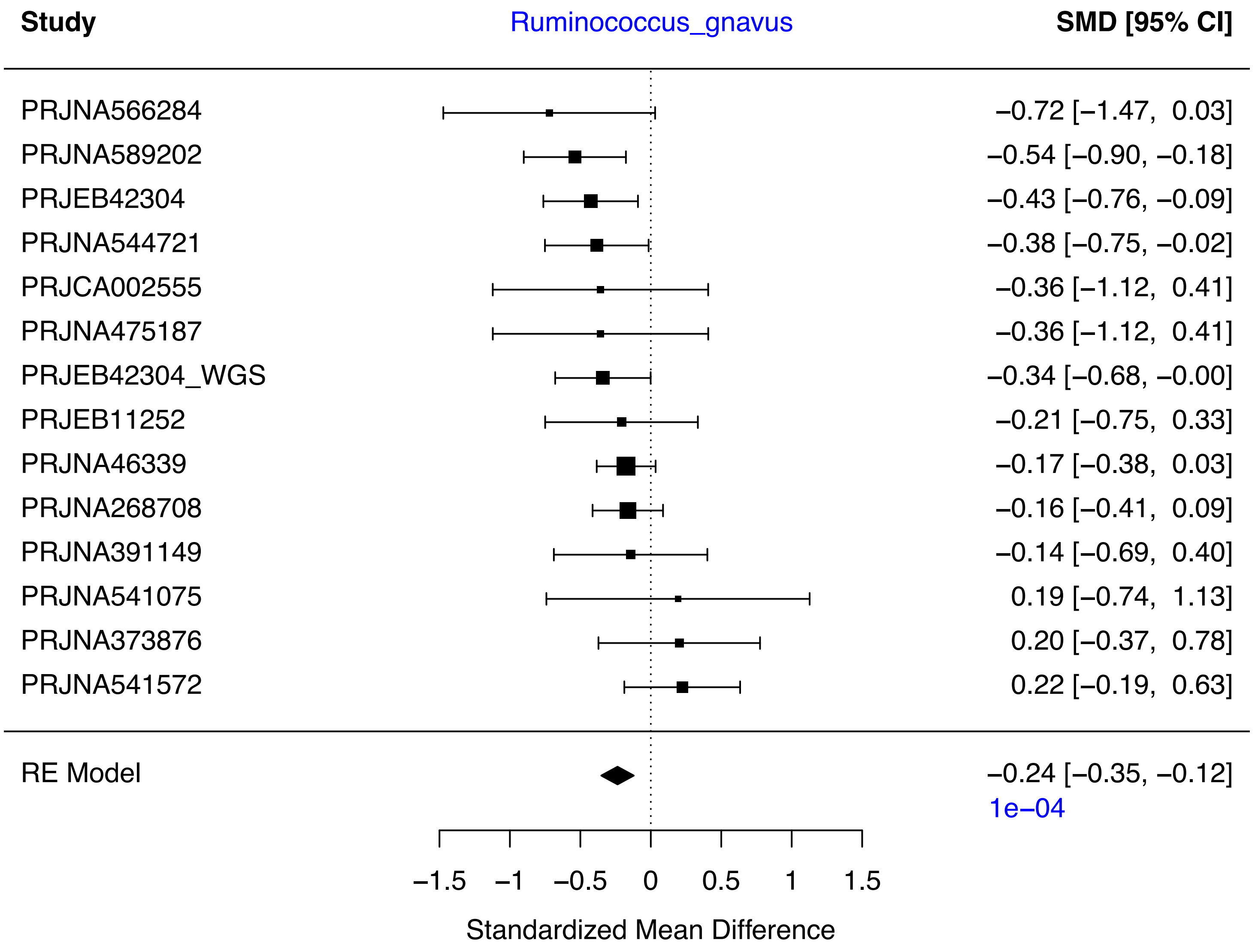
Study
Ruminococcus_gnavus
SMD [95% CI]
PRJNA566284 −0.72 [−1.47, 0.03]
PRJNA589202 −0.54 [−0.90, −0.18]
PRJEB42304 −0.43 [−0.76, −0.09]
PRJNA544721 −0.38 [−0.75, −0.02]
PRJCA002555 −0.36 [−1.12, 0.41]
PRJNA475187 −0.36 [−1.12, 0.41]
PRJEB42304_WGS −0.34 [−0.68, −0.00]
PRJEB11252 −0.21 [−0.75, 0.33]
PRJNA46339 −0.17 [−0.38, 0.03]
PRJNA268708 −0.16 [−0.41, 0.09]
PRJNA391149 −0.14 [−0.69, 0.40]
PRJNA541075 0.19 [−0.74, 1.13]
PRJNA373876 0.20 [−0.37, 0.78]
PRJNA541572 0.22 [−0.19, 0.63]
RE Model −0.24 [−0.35, −0.12]
1e−04
−1.5 −1 −0.5 0 0.5 1 1.5
Standardized Mean Difference

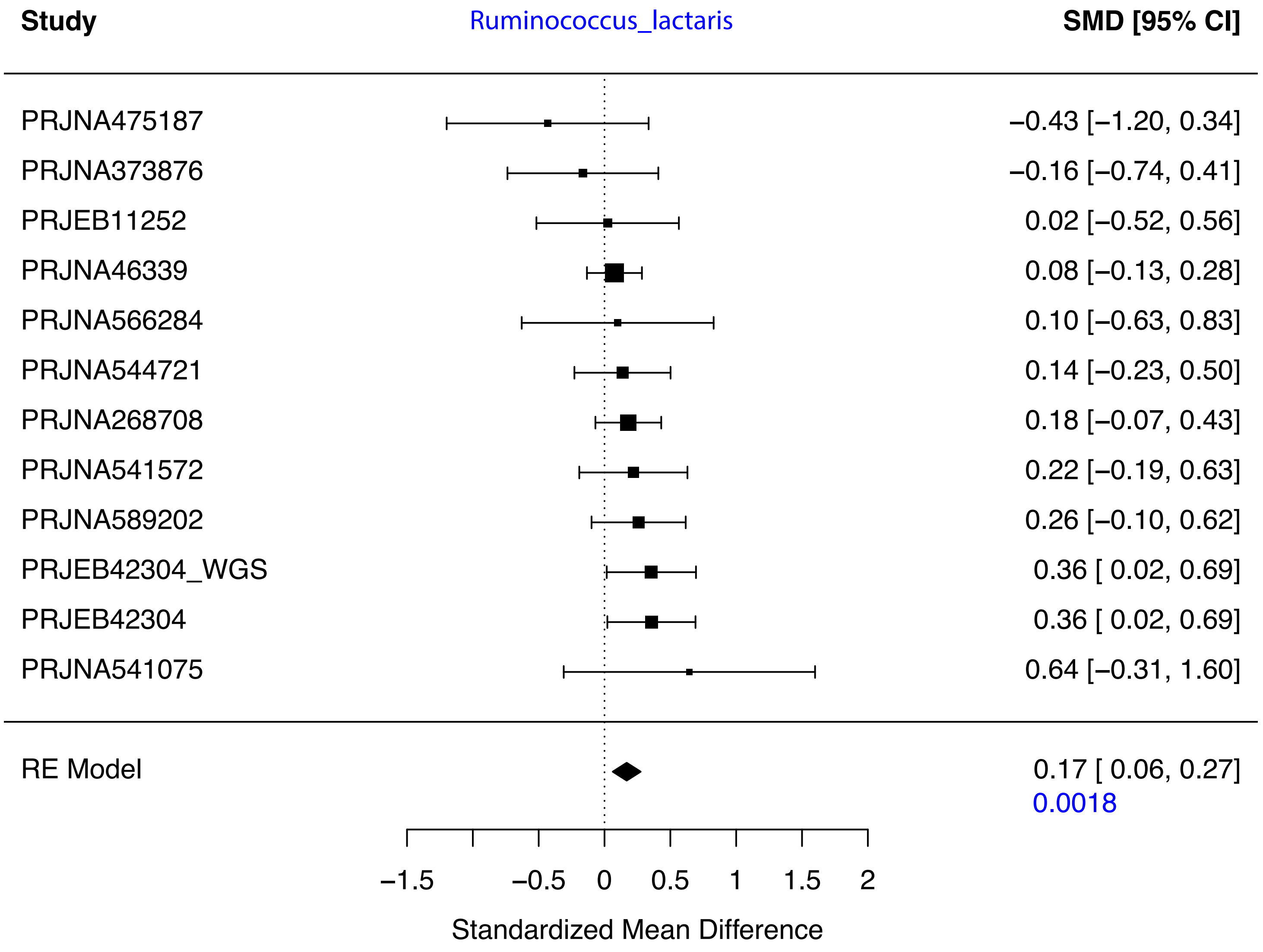
Study
Ruminococcus_lactaris
SMD [95% CI]
PRJNA475187 -0.43 [-1.20, 0.34]
PRJNA373876 -0.16 [-0.74, 0.41]
PRJEB11252 0.02 [-0.52, 0.56]
PRJNA46339 0.08 [-0.13, 0.28]
PRJNA566284 0.10 [-0.63, 0.83]
PRJNA544721 0.14 [-0.23, 0.50]
PRJNA268708 0.18 [-0.07, 0.43]
PRJNA541572 0.22 [-0.19, 0.63]
PRJNA589202 0.26 [-0.10, 0.62]
PRJEB42304_WGS 0.36 [0.02, 0.69]
PRJEB42304 0.36 [0.02, 0.69]
PRJNA541075 0.64 [-0.31, 1.60]
RE Model 0.17 [0.06, 0.27]
0.0018
-1.5
-0.5
0
0.5
1
1.5
2
Standardized Mean Difference

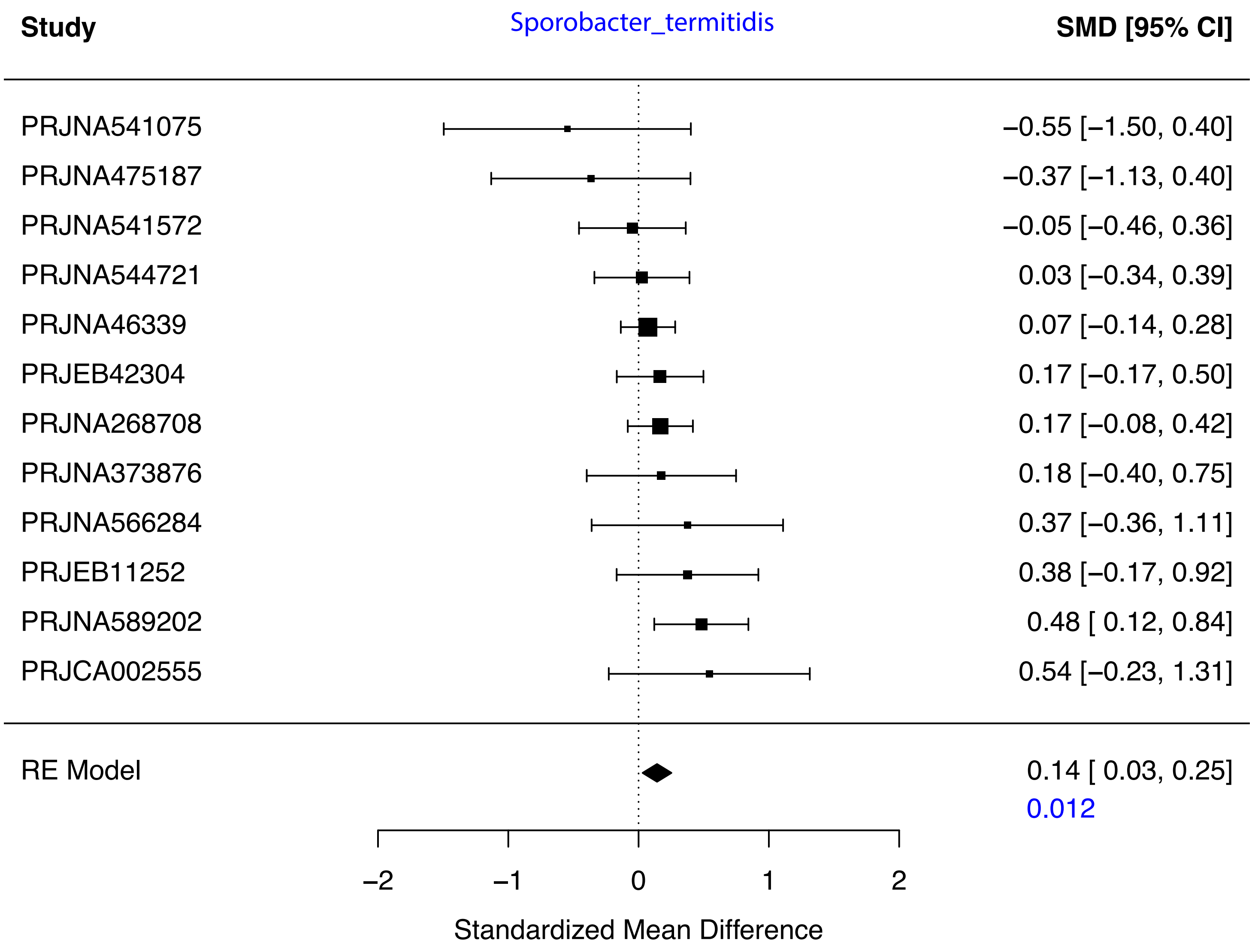
Study
Sporobacter_termitidis
SMD [95% CI]
PRJNA541075 −0.55 [−1.50, 0.40]
PRJNA475187 −0.37 [−1.13, 0.40]
PRJNA541572 −0.05 [−0.46, 0.36]
PRJNA544721 0.03 [−0.34, 0.39]
PRJNA46339 0.07 [−0.14, 0.28]
PRJEB42304 0.17 [−0.17, 0.50]
PRJNA268708 0.17 [−0.08, 0.42]
PRJNA373876 0.18 [−0.40, 0.75]
PRJNA566284 0.37 [−0.36, 1.11]
PRJEB11252 0.38 [−0.17, 0.92]
PRJNA589202 0.48 [0.12, 0.84]
PRJCA002555 0.54 [−0.23, 1.31]
RE Model 0.14 [0.03, 0.25]
0.012
−2
−1
0
1
2
Standardized Mean Difference

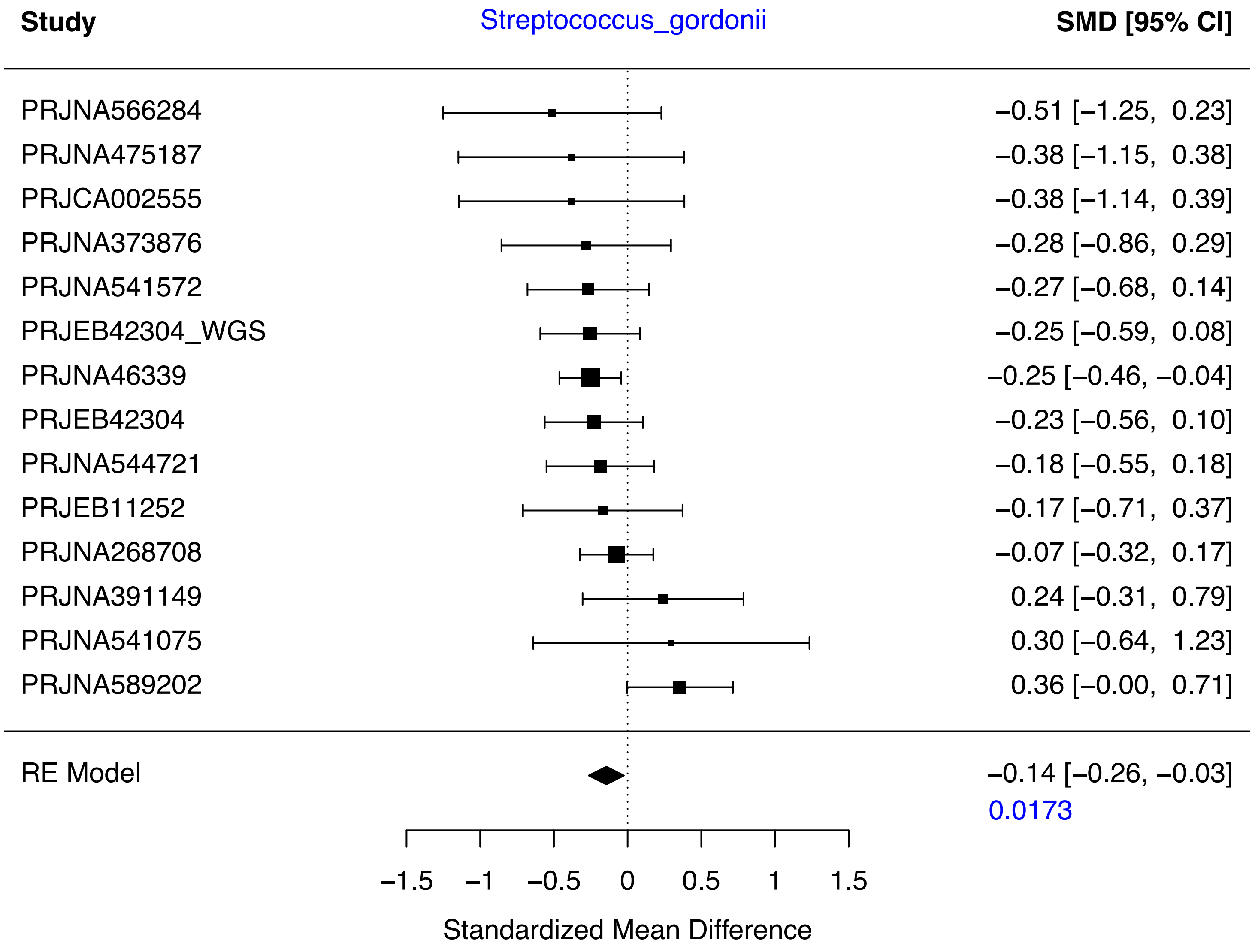
Study
Streptococcus_gordonii
SMD [95% CI]
PRJNA566284
−0.51 [−1.25, 0.23]
PRJNA475187
−0.38 [−1.15, 0.38]
PRJCA002555
−0.38 [−1.14, 0.39]
PRJNA373876
−0.28 [−0.86, 0.29]
PRJNA541572
−0.27 [−0.68, 0.14]
PRJEB42304_WGS
−0.25 [−0.59, 0.08]
PRJNA46339
−0.25 [−0.46, −0.04]
PRJEB42304
−0.23 [−0.56, 0.10]
PRJNA544721
−0.18 [−0.55, 0.18]
PRJEB11252
−0.17 [−0.71, 0.37]
PRJNA268708
−0.07 [−0.32, 0.17]
PRJNA391149
0.24 [−0.31, 0.79]
PRJNA541075
0.30 [−0.64, 1.23]
PRJNA589202
0.36 [−0.00, 0.71]
RE Model
−0.14 [−0.26, −0.03]
0.0173
−1.5
−1
−0.5
0
0.5
1
1.5
Standardized Mean Difference

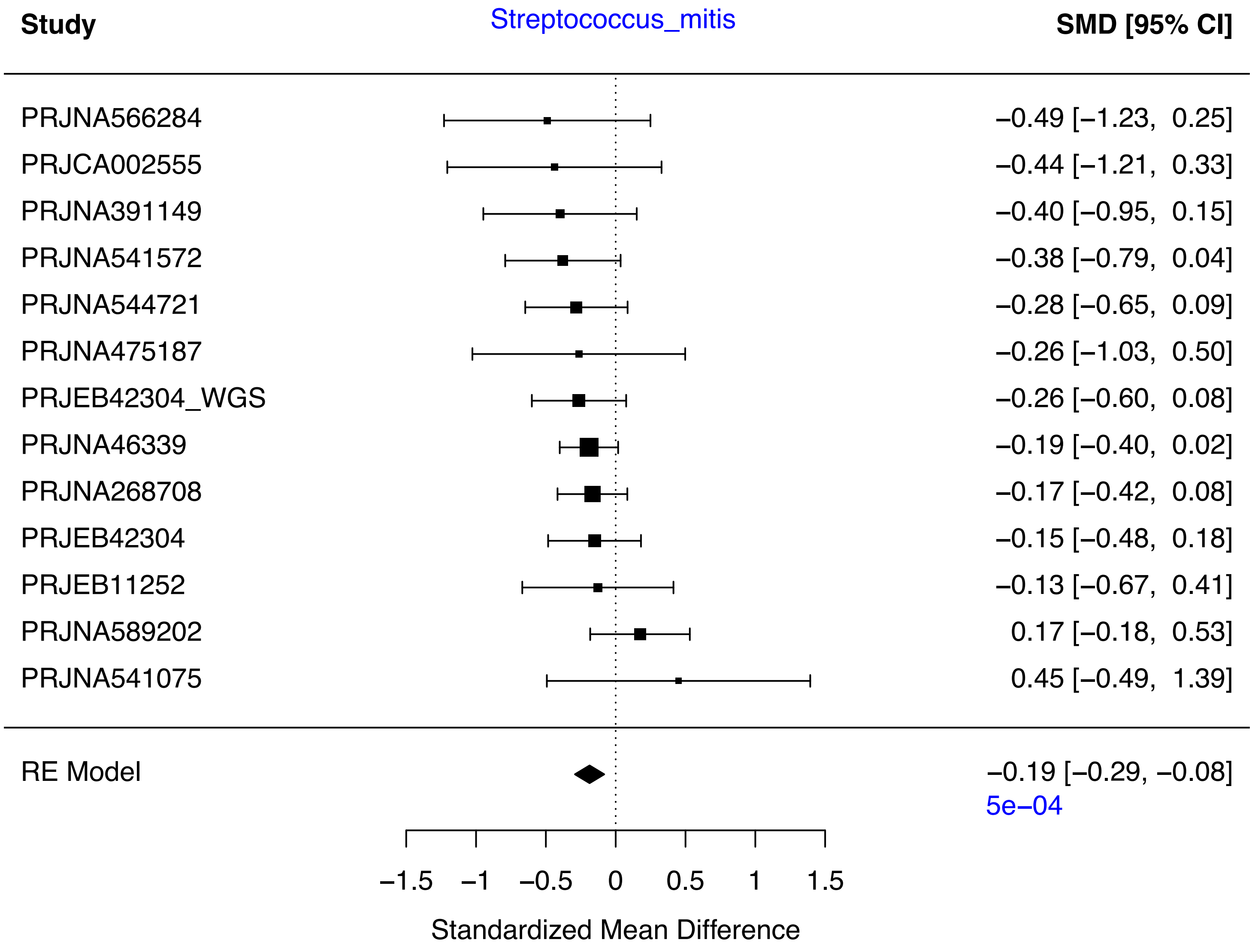
Study
Streptococcus_mitis
SMD [95% CI]
PRJNA566284 −0.49 [−1.23, 0.25]
PRJCA002555 −0.44 [−1.21, 0.33]
PRJNA391149 −0.40 [−0.95, 0.15]
PRJNA541572 −0.38 [−0.79, 0.04]
PRJNA544721 −0.28 [−0.65, 0.09]
PRJNA475187 −0.26 [−1.03, 0.50]
PRJEB42304_WGS −0.26 [−0.60, 0.08]
PRJNA46339 −0.19 [−0.40, 0.02]
PRJNA268708 −0.17 [−0.42, 0.08]
PRJEB42304 −0.15 [−0.48, 0.18]
PRJEB11252 −0.13 [−0.67, 0.41]
PRJNA589202 0.17 [−0.18, 0.53]
PRJNA541075 0.45 [−0.49, 1.39]
RE Model −0.19 [−0.29, −0.08]
5e−04
−1.5 −1 −0.5 0 0.5 1 1.5
Standardized Mean Difference

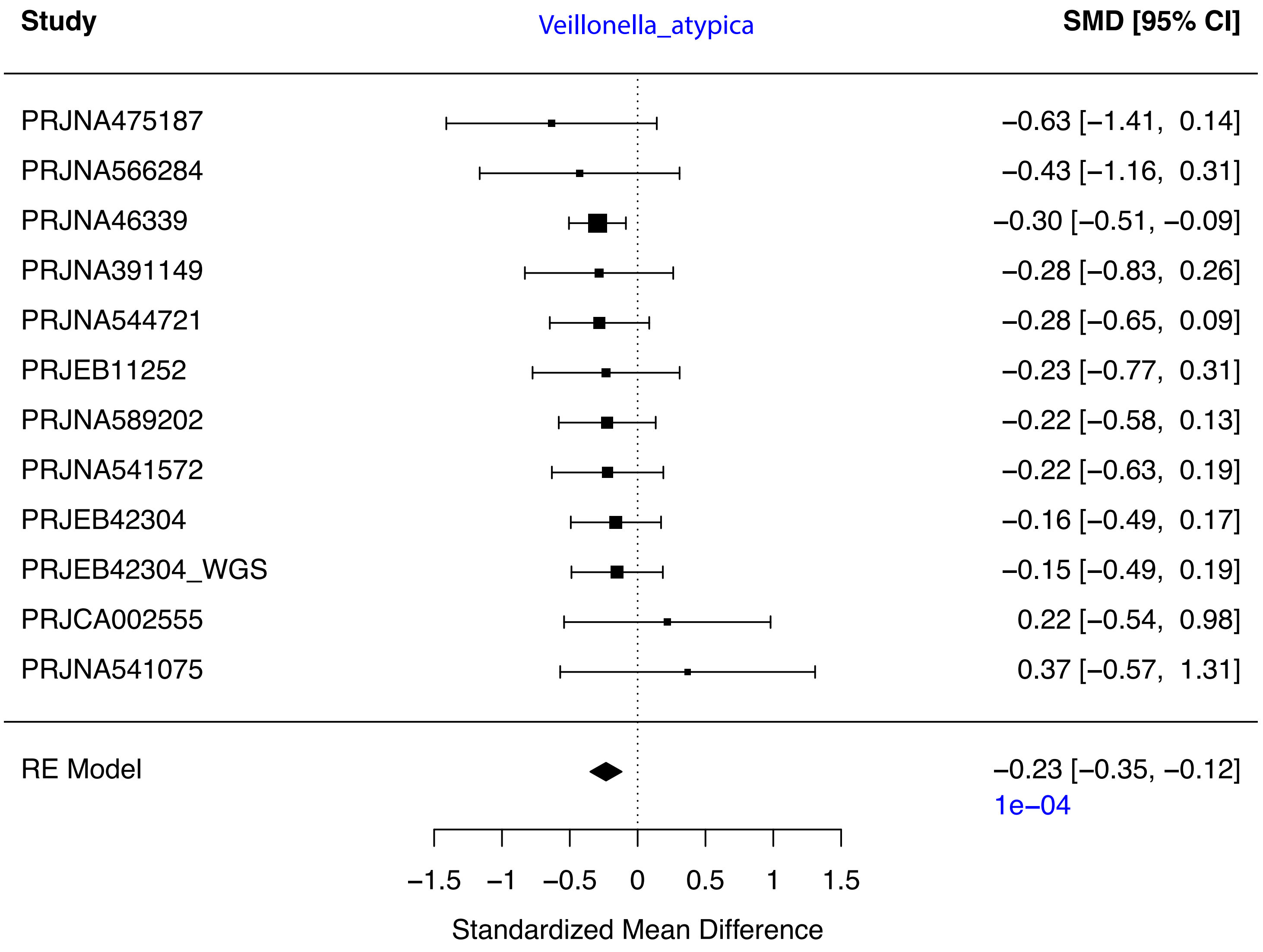
Study
Veillonella_atypica
SMD [95% CI]
PRJNA475187
PRJNA566284
PRJNA46339
PRJNA391149
PRJNA544721
PRJEB11252
PRJNA589202
PRJNA541572
PRJEB42304
PRJEB42304_WGS
PRJCA002555
PRJNA541075
−0.63 [−1.41, 0.14]
−0.43 [−1.16, 0.31]
−0.30 [−0.51, −0.09]
−0.28 [−0.83, 0.26]
−0.28 [−0.65, 0.09]
−0.23 [−0.77, 0.31]
−0.22 [−0.58, 0.13]
−0.22 [−0.63, 0.19]
−0.16 [−0.49, 0.17]
−0.15 [−0.49, 0.19]
0.22 [−0.54, 0.98]
0.37 [−0.57, 1.31]
RE Model
−0.23 [−0.35, −0.12]
1e−04
−1.5
−1
−0.5
0
0.5
1
1.5
Standardized Mean Difference

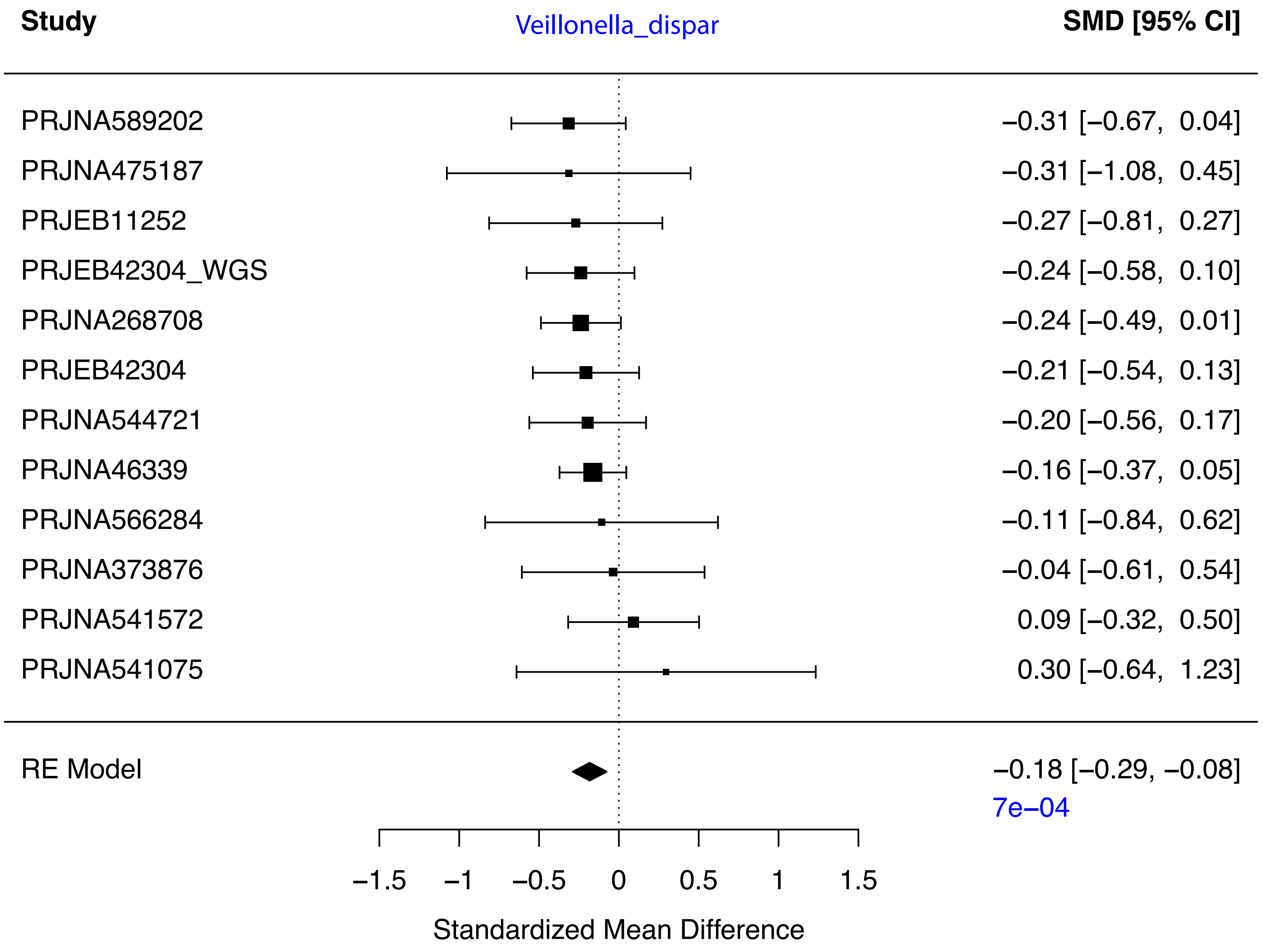
Study
Veillonella_dispar
SMD [95% CI]
PRJNA589202 −0.31 [−0.67, 0.04]
PRJNA475187 −0.31 [−1.08, 0.45]
PRJEB11252 −0.27 [−0.81, 0.27]
PRJEB42304_WGS −0.24 [−0.58, 0.10]
PRJNA268708 −0.24 [−0.49, 0.01]
PRJEB42304 −0.21 [−0.54, 0.13]
PRJNA544721 −0.20 [−0.56, 0.17]
PRJNA46339 −0.16 [−0.37, 0.05]
PRJNA566284 −0.11 [−0.84, 0.62]
PRJNA373876 −0.04 [−0.61, 0.54]
PRJNA541572 0.09 [−0.32, 0.50]
PRJNA541075 0.30 [−0.64, 1.23]
RE Model −0.18 [−0.29, −0.08]
7e−04
−1.5
−1
−0.5
0
0.5
1
1.5
Standardized Mean Difference

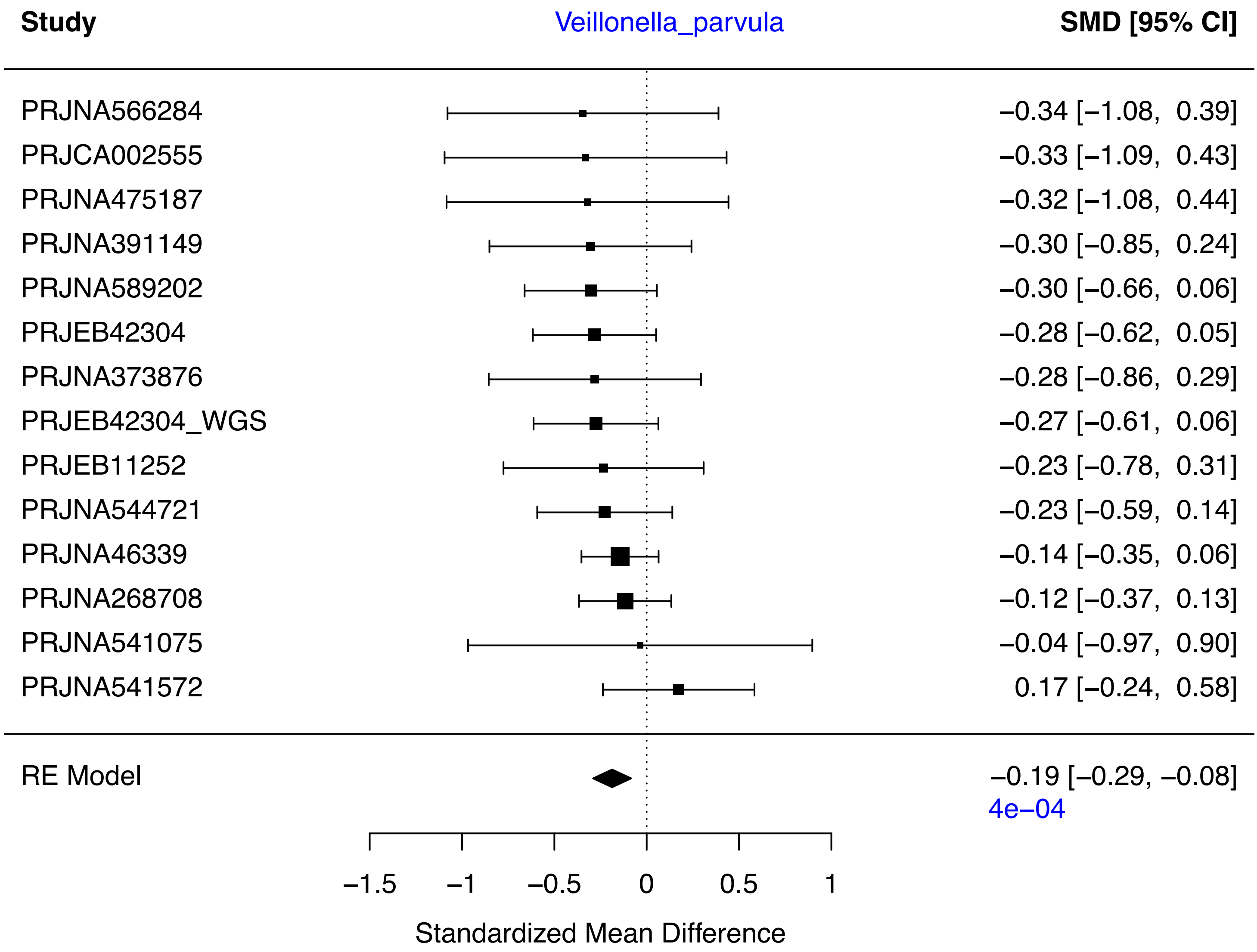
Study
Veillonella_parvula
SMD [95% CI]
PRJNA566284 −0.34 [−1.08, 0.39]
PRJCA002555 −0.33 [−1.09, 0.43]
PRJNA475187 −0.32 [−1.08, 0.44]
PRJNA391149 −0.30 [−0.85, 0.24]
PRJNA589202 −0.30 [−0.66, 0.06]
PRJEB42304 −0.28 [−0.62, 0.05]
PRJNA373876 −0.28 [−0.86, 0.29]
PRJEB42304_WGS −0.27 [−0.61, 0.06]
PRJEB11252 −0.23 [−0.78, 0.31]
PRJNA544721 −0.23 [−0.59, 0.14]
PRJNA46339 −0.14 [−0.35, 0.06]
PRJNA268708 −0.12 [−0.37, 0.13]
PRJNA541075 −0.04 [−0.97, 0.90]
PRJNA541572 0.17 [−0.24, 0.58]
RE Model −0.19 [−0.29, −0.08]
4e−04
−1.5
−1
−0.5
0
0.5
1
Standardized Mean Difference

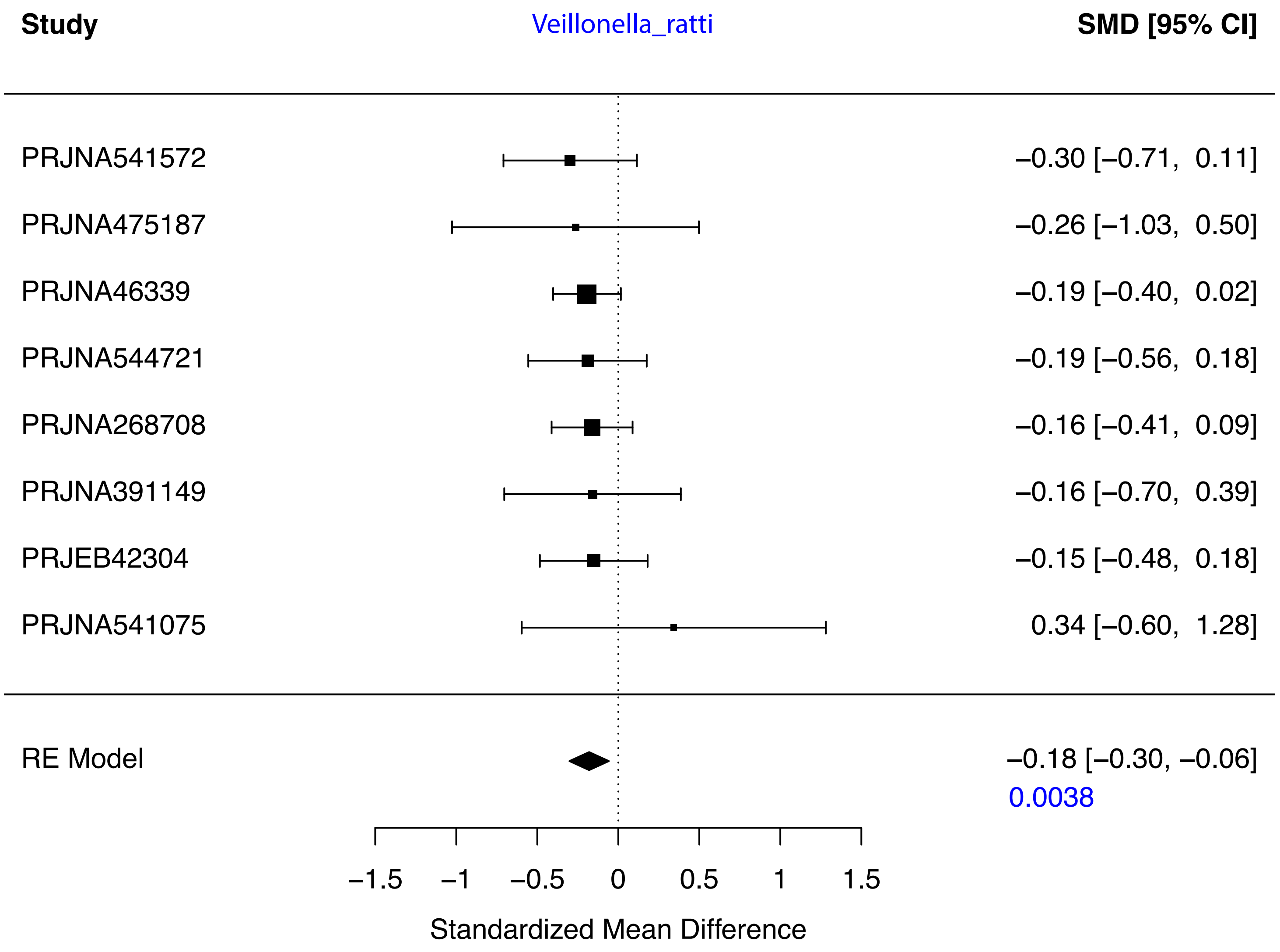
Study
Veillonella_ratti
SMD [95% CI]
PRJNA541572 −0.30 [−0.71, 0.11]
PRJNA475187 −0.26 [−1.03, 0.50]
PRJNA46339 −0.19 [−0.40, 0.02]
PRJNA544721 −0.19 [−0.56, 0.18]
PRJNA268708 −0.16 [−0.41, 0.09]
PRJNA391149 −0.16 [−0.70, 0.39]
PRJEB42304 −0.15 [−0.48, 0.18]
PRJNA541075 0.34 [−0.60, 1.28]
RE Model −0.18 [−0.30, −0.06]
0.0038
−1.5
−1
−0.5
0
0.5
1
1.5
Standardized Mean Difference
